# A Direct Arylation Approach toward Thermally Activated Delayed Fluorescence-Active Benzo[*c*][1,2,5]thiadiazole Emitters for Near-Infrared Solution-Processed OLEDs

**DOI:** 10.1021/acsaom.5c00340

**Published:** 2025-10-20

**Authors:** Sonny Brebels, Emma V. Puttock, Tom Cardeynaels, Kamile Bareikaite, Lucy A. Weatherill, Melissa Van Landeghem, Huguette Penxten, Andrew Danos, Koen Vandewal, Andrew P. Monkman, Benoît Champagne, Wouter Maes

**Affiliations:** † Institute for Materials Research (IMO-IMOMEC), 54496 Hasselt University, Martelarenlaan 42, Hasselt B-3500, Belgium; ‡ IMOMEC Division, IMEC, Wetenschapspark 1, Diepenbeek B-3590, Belgium; § Department of Physics, OEM Group, 3057 Durham University, South Road, Durham DH1 3LE, U.K.; ∥ Laboratory of Theoretical Chemistry, Theoretical and Structural Physical Chemistry Unit, Namur Institute of Structured Matter, 54501 University of Namur, Rue de Bruxelles 61, Namur B-5000, Belgium; ⊥ School of Physical and Chemical Sciences, 4617 Queen Mary University of London, 327 Mile End Road, London E1 4NS, U.K.

**Keywords:** OLEDs, near-infrared, thermally activated delayed fluorescence, direct arylation, solution-processed

## Abstract

An isomeric emitter (**2TPA-iCNBT**) is designed and synthesized, displaying enhanced thermally activated delayed fluorescence (TADF) properties as compared to the reference near-infrared (NIR) emitter TPACNBz (hereafter referred to as **2TPA-CNBT**). Its modified benzo­[*c*]­[1,2,5]­thiadiazole-4,7-dicarbonitrile (**iCNBT**) acceptor (A) core positions the two triphenylamine (TPA) donor (D) units adjacently, thereby increasing the D–A torsion angle. Synthesis is realized through the use of an unexploited direct arylation strategy, which, besides offering the desired materials in an efficient and straightforward way, can also yield monofunctionalized emitters (**1TPA-CNBT** and **1TPA-iCNBT**). In total, four emitters are synthesized, characterized, and subsequently compared in terms of their spectroscopic and device properties. Density functional theory is applied to simulate their relative molecular geometry and the arrangement of their (emissive) excited states. Steady-state and time-resolved emission spectroscopy reveal strongly contrasting TADF properties, with **2TPA-iCNBT** exhibiting the largest increase in the photoluminescence quantum yield on removal of oxygen (from 27 to 55%), and the fastest TADF emission kinetics in doped films (*k*_RISC_ ∼ 10^5^ s^–1^). In solution-processed organic light-emitting diodes, decent maximum external quantum efficiency (EQE) values are obtained for **2TPA-iCNBT** (2.49%), **1TPA-CNBT** (2.91%), and **1TPA-iCNBT** (2.76%), in clear contrast to **2TPA-CNBT** (1.16%), highlighting the decisive role of the D–A substitution pattern (and the number of D groups) on the performance of NIR-TADF emitters. Furthermore, **2TPA-iCNBT** is shown to maintain the highest EQE at larger current densities (EQE = 1.98% at 10 mA cm^–2^) within the investigated series, a consequence of its standout TADF behavior.

## Graphical abstract

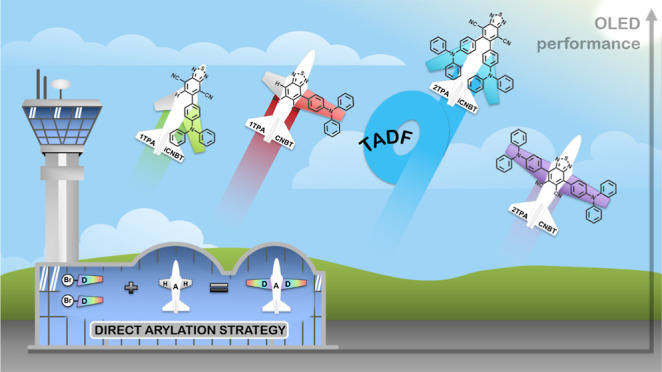

## 1 Introduction

The development of organic chromophores with efficient emission in the near-infrared region (NIR; 650–1400 nm)^1^ holds significant relevance for emerging applications such as fingerprinting and security, extended (in)­visible light communication, spectroscopy, biosensors, and photodynamic therapy.^2–10^ The desired NIR wavelength of emission can be achieved by designing chromophores with large π-conjugated systems and/or pronounced charge-transfer (CT) character through the use of strong electron-donating and -accepting moieties.^11^ Unfortunately, shifting emission deeper into the NIR is often accompanied by a reduced luminescence due to the “energy gap law”, in which the low energy of the excited singlet state increases the nonradiative coupling to the vibrational overtones of the ground state, thus yielding a reduced emission efficiency.^12,13^ While this can be counteracted by improving the rigidity of π-conjugated systems (which dampens vibrational modes),^14^ such rigidity often leads to the formation of planar structures which are highly susceptible to undesired molecular stacking. In most cases, this gives rise to aggregation-induced quenching, again resulting in a significant loss of the fluorescence efficiency,^15^ as well as solubility issues during synthesis and purification.

The limited performance of deep-red and NIR emitters is further exacerbated by poor exciton utilization in conventional fluorescence-based electroluminescent devices, which limits their internal quantum efficiency (IQE) to 25%. To overcome this hurdle, much research attention has been focused on the highly promising triplet harvesting mechanism of thermally activated delayed fluorescence (TADF).^16–19^ In TADF, excitons from the nonemissive triplet state(s) are upconverted to emissive singlet states via reverse intersystem crossing (RISC), unlocking potential IQEs of up to 100% from injected charges. This spin-flip is promoted by the available thermal energy of the environment, and becomes active when the singlet–triplet energy gap (Δ*E*_ST_) is minimized.^20,21^ In CT type emitters, TADF behavior can be induced by introducing a twist between electron-donor (D) and electron-acceptor (A) units, which limits the wave function overlap of their respective localized HOMO (highest occupied) and LUMO (lowest unoccupied) frontier molecular orbitals. In this way, a sufficiently small Δ*E*_ST_ (typically ≤0.2 eV) can be obtained.^22,23^ Nonetheless, some degree of orbital overlap is necessary to maintain electronic communication between the D and A units and to permit high emission quantum yields.^24^ The small energy gap between states of differing excitonic character ultimately supports efficient RISC, promoting rapid upconversion from the triplet state. This circumvents competitive triplet quenching mechanism in devices, leading to both higher efficiencies and improved stability with reduced efficiency roll-off.^25^

Further structural fine-tuning to maximize the performance of candidate TADF emitters is often explored through the design of different regio-isomers, involving the relocation, addition, or removal of (auxiliary) donor units and/or acceptor groups (e.g. nitriles, fluorines, pyridines).^26–31^ For red/NIR-emissive fluorophores containing acceptor moieties with multiple attachment positions available, one strategy entails going from T- to Y-shaped molecules, where the close proximity of the two donor units in the latter is expected to benefit the overall TADF properties.^32–36^ This is linked to an increased dihedral angle between the D and A unit(s) due to steric interactions (tuning the HOMO/LUMO overlap), and a better interplay between the relevant singlet and triplet states. For instance, Wang et al. highlighted the beneficial (co)­linear orientation of the D and A units in the oTPA-DPPz emitter, resulting in a larger (but still sufficiently small) frontier molecular orbital overlap, which greatly improved the transition probability—as well as the photoluminescence quantum yield (PLQY)—of the emissive CT state.^33^ Later, Xie et al. demonstrated how isomeric regulation between ortho- (Y) and para-(T) linkages could effectively switch “on” the TADF mechanism in IQ-oTPA, with the ortho-isomer exhibiting a significantly smaller Δ*E*_ST_ (and thus efficient TADF), whereas the para-isomer showed only conventional fluorescence.^34^ More recently, Liu et al. revealed the presence of an additional, close-lying locally excited (LE) triplet state in the Y-shaped, acenaphtho­[1,2-*b*]­quinoxaline-3,4-dicarbonitrile-containing LB-Y emitter, and its relevance in improving the TADF process giving rise to faster RISC. The rigid and sterically hindered nature of Y-shaped emitters was also suggested to suppress the nonradiative decay channels that can arise from intramolecular vibrations and rotations, leading to a higher PLQY.^35^ A small overview with a few reported examples of T and Y-shaped emitters is given in Figure 1.

**Figure 1:**
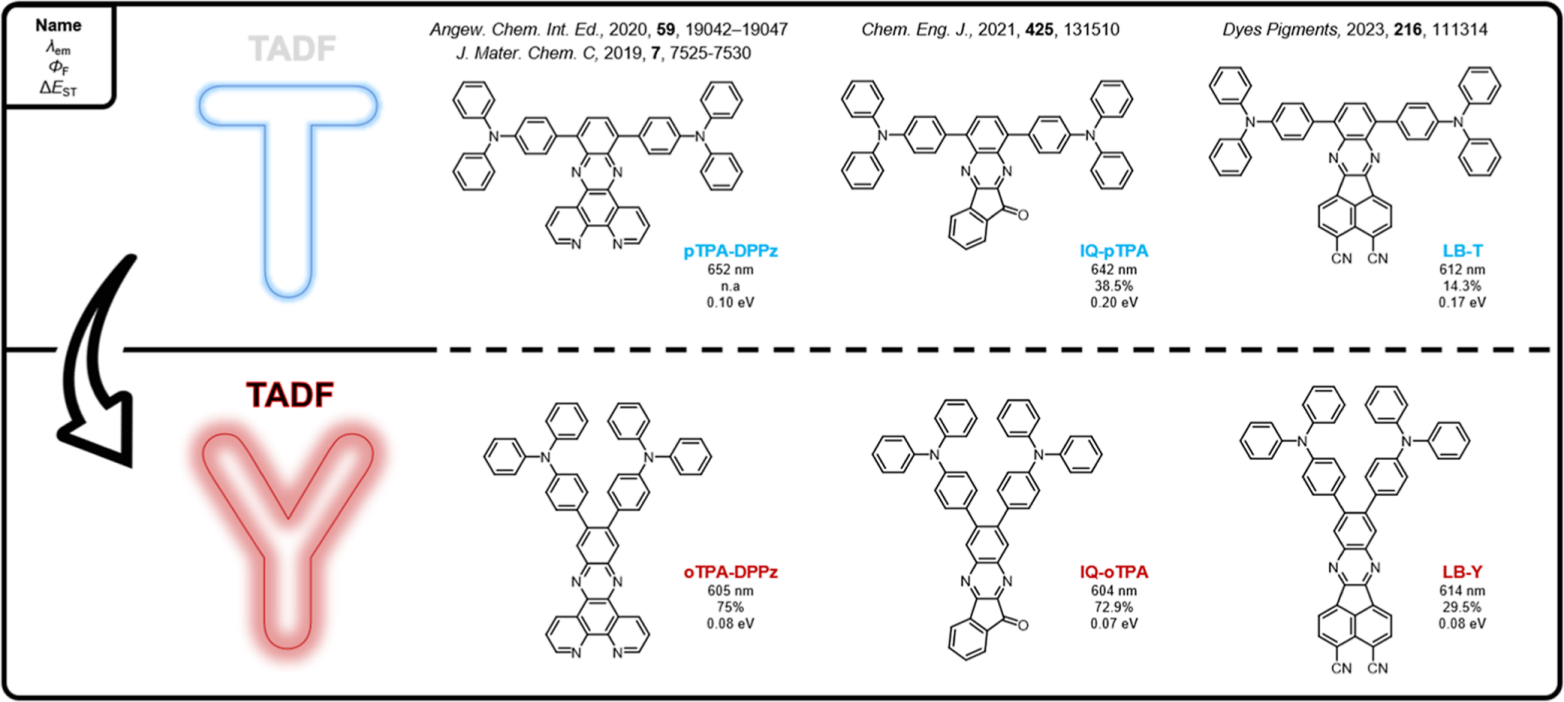
Examples and comparison of reported red T- and Y-shaped TADF emitters. The main emission characteristics (emission wavelength maximum, fluorescence quantum yield, and singlet–triplet energy gap) in doped films are given.

In this study, the TADF performance of the previously reported T-shaped emitter 4,7-bis­(4-(diphenylamino)­phenyl)-benzo­[*c*]­[1,2,5]­thiadiazole-5,6-dicarbonitrile (**2TPA-CNBT**)^37^ was compared to the novel Y-shaped analog, 5,6-bis­(4-(diphenylamino)­phenyl)­benzo­[*c*]­[1,2,5]­thiadiazole-4,7-dicarbonitrile (**2TPA-iCNBT**, Figure 2). These compounds were readily synthesized from commercially available starting products, utilizing a direct arylation strategy as a straightforward method to access the core positions of (i)­CNBT. Additionally, this method also allows efficient preparation of the single-donor functionalized fluorophores **1TPA-CNBT** and **1TPA-iCNBT**, enabling comparison of D–A and D–A–D architectures, a topic of similar broad interest (Figure 2).^38–41^ To support deeper insight into both individual properties and comparative trends, all compounds were investigated by time-dependent density functional theory (TDDFT) as well as steady-state and time-resolved spectroscopy in solution and doped films, revealing surprising differences in TADF behavior and overall performance. In solution-processed organic light-emitting diode (OLED) devices, the **2TPA-iCNBT** isomer was found to maintain the highest external quantum efficiency (EQE) at higher current densities, correlating to its pronounced delayed fluorescence and short delayed lifetime as measured in 4,4′-bis­(*N*-carbazolyl)-1,1′-biphenyl (CBP) films. These results thus showcase our further understanding of isomeric effects and their impact on TADF properties and OLED performance.

**Figure 2:**
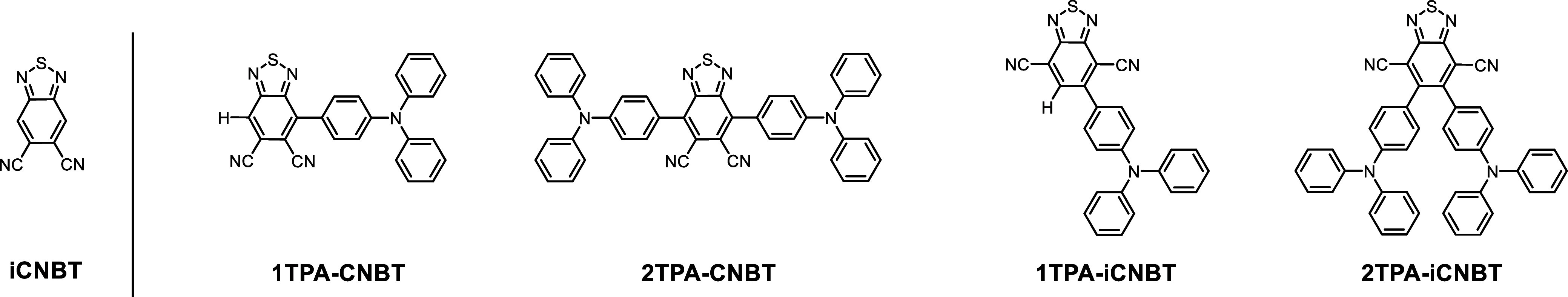
Structural overview of the **(i)­CNBT** template and the explored emitters **1TPA-CNBT**, **2TPA-CNBT**, **1TPA-iCNBT**, and **2TPA-iCNBT**.

## 2 Results and Discussion

### 2.1 Synthesis

A two-step synthetic pathway was previously reported for **2TPA-CNBT**, starting from 4,7-dibromo-5,6-difluorobenzo-[*c*]­[1,2,5]­thiadiazole (Scheme 1).^37^ In the first step, the benzo­[*c*]­[1,2,5]­thiadiazole core is coupled with a borylated triphenylamine donor unit using a Suzuki–Miyaura cross-coupling reaction. This is followed by a substitution of the fluorine groups with nitriles, using potassium cyanide, yielding the final compound. Unfortunately, several attempts to synthesize **2TPA-CNBT** (**3**) through this method proved largely unsuccessful (Scheme 1). Moreover, such a pathway would not be available for **1TPA-iCNBT** (**10**) and **2TPA-iCNBT** (**11**), since the required (halogenated) precursor is neither commercially available nor easily accessible. Hence, another pathway was developed, employing the direct arylation strategy of Zhang et al.,^42^ thereby expanding its scope to include potentially interesting TADF scaffolds. This allowed all four emitters to be obtained in a quick, elegant, and efficient way from cheap and commercially available compounds. Full details on the synthetic procedures and structural characterization data are provided in the Supporting Information.

**Scheme 1:**
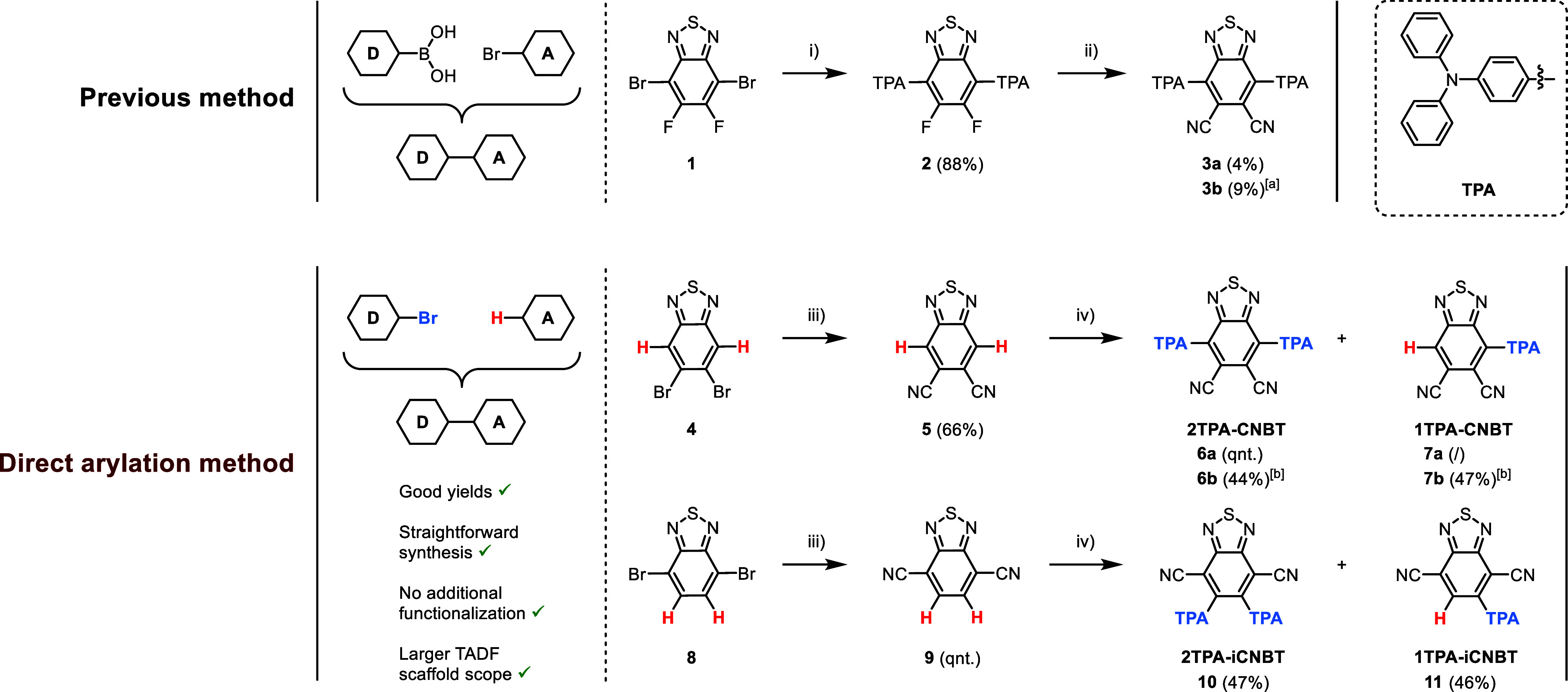
Synthesis Protocol for **2TPA-CNBT** (**3** and **6**), **1TPA-CNBT** (**7**), **2TPA-iCNBT** (**10**), and **1TPA-iCNBT** (**11**): (i) (4-(Diphenylamino)­phenyl)­boronic Acid, Pd­(PPh_3_)_4_, K_2_CO_3_, Toluene, Ethanol, Water, Reflux, 48 h; (ii) KCN, 18-Crown-6, THF, Reflux, 24 h; (iii) CuCN, NMP, 170 °C, 2 h; (iv) 4-Bromo-*N*,*N*-diphenylaniline, Pd­(OAc)_2_, PtBu_2_Me·HBF_4_, Pivalic Acid, K_2_CO_3_, Toluene, Reflux, 24 h; [a] Second Addition of KCN and 18-Crown-6, and Left to Stir for an additional 48 h; [b] 1.1 equiv of 4-Bromo-*N*,*N*-diphenylaniline instead of 2.2

### 2.2 Computational Analysis and (TD)­DFT Calculations

Density functional theory (DFT) calculations (M06/6-311G­[d]) were performed to determine the optimized molecular geometries of **2TPA-CNBT**, **1TPA-CNBT**, **2TPA-iCNBT**, and **1TPA-iCNBT**. Subsequently, two singlet (S_1_, S_2_) and three triplet (T_1,_ T_2,_ T_3_) vertical excited state energies (from S_0_) were obtained using time-dependent DFT (TDDFT) calculations using a modified LC-BLYP (ω = 0.17 bohr^–1^) exchange–correlation (XC) functional.^43^ These calculations were performed under the Tamm–Dancoff approximation^44^ (TDA) and the polarizable continuum model (PCM) in methylcyclohexane to simulate a nonpolar environment.^45^ The Gaussian16 package was utilized to perform all calculations.^46^ The orbital spatial distributions were obtained from single-point calculations using the same LC-BLYP(17)/6-311G­[d] method. The CT character of the involved states was investigated through electron density difference mapping (EDDM), which shows the changing electron density in a molecule upon a transition from the ground state to a specific excited state. These CT characters are described by the amount of electrons transferred during the transition (*q*_CT_), the distance over which the electronic charge is transferred (*d*_CT_), and the related change in dipole moment (Δμ), calculated as described by Le Bahers and co-workers.^47^ The distance over which the electronic charge is transferred is also calculated using the Earth Mover’s method (^EM^ *d*_CT_) as reported by Fraiponts et al.,^48^ to correct for the highly symmetric nature of **2TPA-CNBT**. To enable this, electron density was approximated by partial atomic charges using an electrostatic potential (CHelpG) charge model.^49^ Changing electron densities in both the donor and acceptor unit are indicative of a CT excited state, while confined changes in the same units (similar molecular distribution) point to an LE state instead. For the dominant excited states, a natural transition orbital (NTO) analysis was also carried out, providing the dominant pairs of particle and hole NTO’s.^50^ Subsequently, to complementary address the LE/CT characters of the transitions, the overlaps between the NTO pairs have been calculated, following the method proposed by Peach and co-workers.^51^ The NTO overlap calculations were performed with the Multiwfn program^52^ and were represented with ChemCraft.^53^ All calculations on the excited states employed the nonequilibrium solvation scheme.^54^ Furthermore, the spin–orbit coupling (SOC) matrix elements were evaluated using the PySOC program using the same XC functional, basis set, and PCM treatment as described above.^55^

Optimization of the molecular geometries shows relatively similar orientations (and dihedral angles) between the uncrowded TPA and (i)­CNBT subunits in **1TPA-CNBT**, **2TPA-CNBT**, and **1TPA-iCNBT** (θ = 40–45°; Figure 3). A noteworthy exception is **2TPA-iCNBT**, where the close proximity of the two TPA units induces an additional steric interaction, resulting in an increase of the dihedral angle to 58°. Meanwhile, very similar molecular orbital distributions are observed in all four materials for the HOMO (localized on the TPA unit) and LUMO/LUMO + 1 (mainly localized on the acceptor). Superimposing the HOMO and LUMO distributions shows a rather limited overlap—which appears to be smallest in **2TPA-iCNBT**—thereby exemplifying the guidelines of typical TADF molecular design. Subsequent TDDFT calculations, EDDMs, and NTO’s were employed to assess the properties (i.e. CT nature and relative energy) of the relevant singlet (S_*x*_) and triplet (T_*x*_) excited states (Table S1, Table S2, and Figure S1–S5) Meanwhile, the strength of the spin–orbit coupling could be derived from the calculated SOC matrix elements. A comprehensive excited state energy diagram is shown in Figure 4, and further tabulated in the Supporting Information (Table S2).

**Figure 3:**
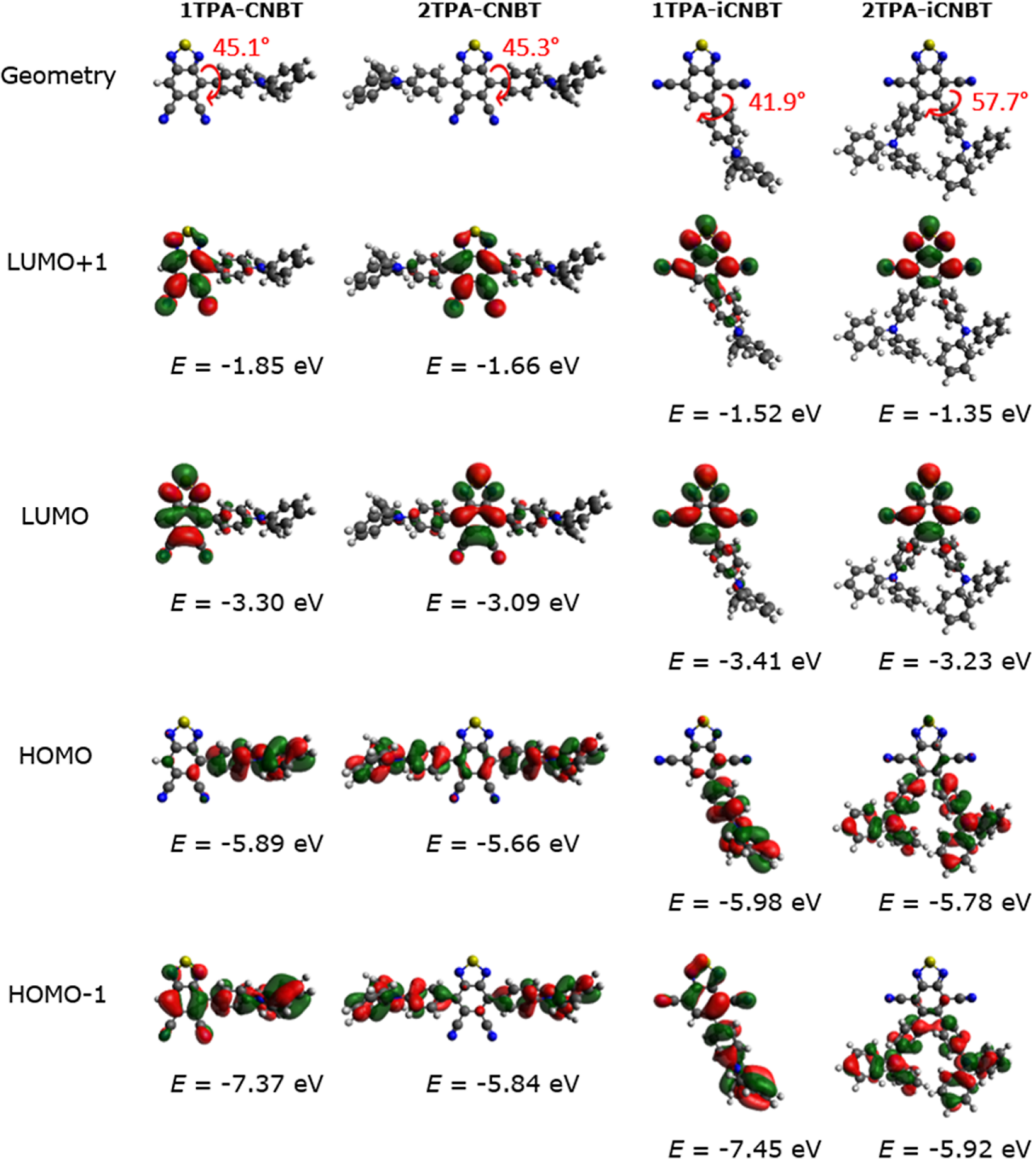
Optimized molecular geometries, energies, and orbital spatial distributions (LUMO + 1, LUMO, HOMO, and HOMO – 1) for **1TPA-CNBT**, **2TPA-CNBT**, **1TPA-iCNBT**, and **2TPA-iCNBT**. Values of dihedral angles are given in red. Isocontour values of 0.02 (au) were used for all orbitals.

**Figure 4:**
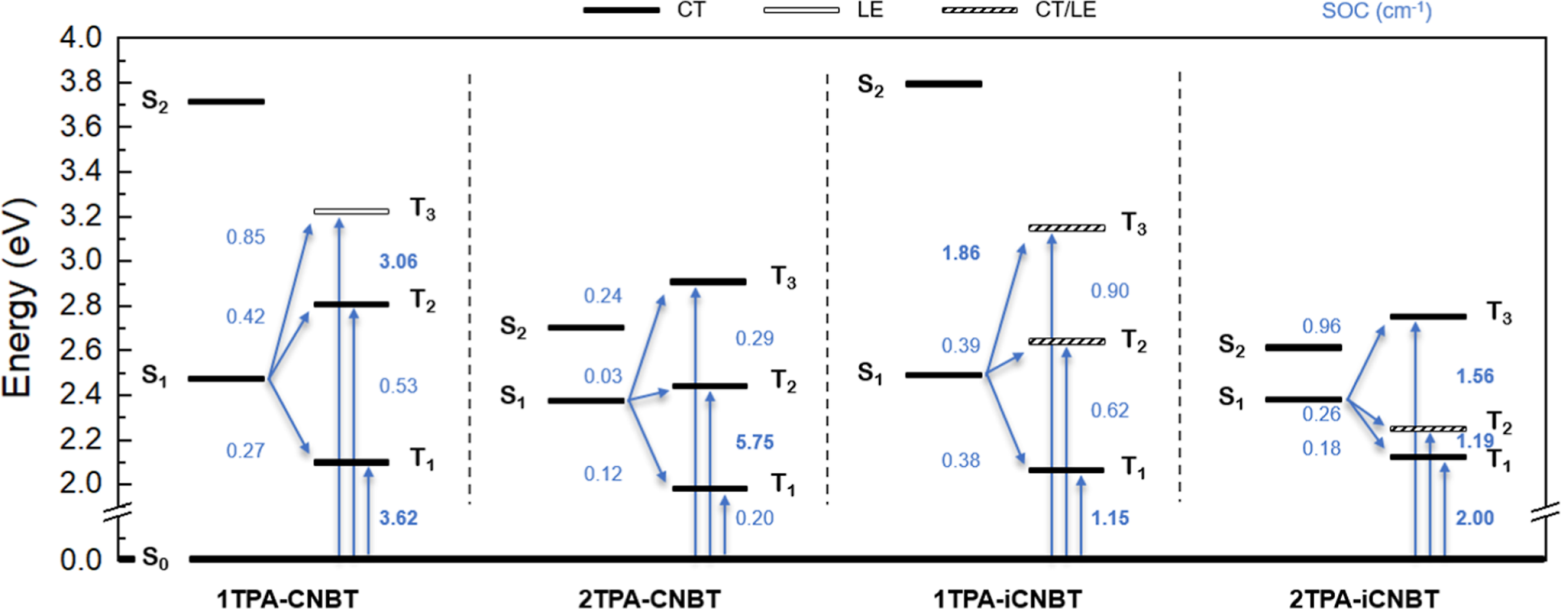
Schematic overview of the excited state energy levels (in eV) of all four emitters with their corresponding CT (black), LE (white), and mixed CT/LE (dashed) character as obtained through TDDFT calculations. Relevant S_0_–T_*x*_ and S_1_–T_*x*_ (*x* = 1–3) couplings and SOC values are shown in blue (SOC values > 1 cm^–1^ in bold).

For each of the four emitters, the S_1_ state is mainly characterized by a HOMO to LUMO transition, indicating a clear CT nature (Table S1). Although this might be less obvious for the S_1_ state of **2TPA-CNBT** (which shows a reduced dipole moment as a result of its diametrically oriented TPA units), its CT character becomes apparent when the ^EM^ *d*_CT_ and associated EDDM/NTO plots are considered (Figures S1–S5). For S_2_, a dominant HOMO – 1 to LUMO transition contributes the most toward the CT nature of each emitter. The main difference here is found in the stabilization of S_2_ in **2TPA-CNBT** and **2TPA-iCNBT** compared to **1TPA-CNBT** and **1TPA-iCNBT**, which partly depends on the varying orbital distributions of the HOMO – 1. Importantly, the presence of an identical donor pair in the (symmetric) **2TPA-CNBT** and **2TPA-iCNBT** structures results in nearly degenerate HOMO/HOMO – 1 energy levels, pushing their S_2_ state much closer to S_1_ when compared to the singlet states of **1TPA-CNBT** and **1TPA-iCNBT**. At the same time, the relative position of the donor unit(s) does not seem to influence the S_1_ energy significantly. This is in contrast with the behavior of the higher-lying triplet states, which show a larger dependency on variations in the molecular structure. Increasing the number and/or changing the position of the donor units causes a downward trend in the energy level of T_2_ (and T_3_), even pushing it below the S_1_ state in **2TPA-iCNBT**. For T_1_, a clear correlation between its varying energy and any structural changes is less obvious. While rather consistent for **1TPA-CNBT** and **1TPA-iCNBT**, the energy of T_1_ appears to vary more significantly among **2TPA-CNBT** and **2TPA-iCNBT**, seemingly depending on the relative proximity and orientation of their TPA donor units. Naturally, this behavior has a profound effect on the resulting Δ*E*_ST_ and on TADF performance. Comparing the different emitters, the theoretical Δ*E*_ST_ decreases from 0.44 eV in **1TPA-iCNBT** and 0.40 eV in **2TPA-CNBT**, down to 0.36 eV in **1TPA-CNBT** and finally 0.26 eV in **2TPA-iCNBT** (Table S2). Consideration should also be given to the role of spin–orbit coupling. According to the El-Sayed rule, spin–orbit coupling more readily enables (R)­ISC when it occurs between states which have different natures (e.g. ^1^CT and ^3^LE).^56,57^ Additionally, the RISC is further enhanced through vibronic coupling (VC) of the T_1_ state with other close-lying triplet states.^14,58,59^ Although relatively larger estimated SOC values (between S_1_ and T_1,_ T_2_ and T_3_) are derived for **1TPA-iCNBT** and **1TPA-CNBT**, the absence of LE or hybrid (CT/LE) triplet states below or close to S_1_ effectively limits RISC, resulting in weaker TADF. Meanwhile, an even worse delayed fluorescence contribution is expected for **2TPA-CNBT**, owing to the poor SOC between its excited states. However, a small SOC value for the ISC (S_1_ to T_1_) and the transition to the ground state (T_1_ to S_0_) does suggest a more suppressed triplet formation in **2TPA-CNBT**. Finally, **2TPA-iCNBT** shows promising potential as the computational investigation reveals a relatively narrow Δ*E*_ST_, a hybrid triplet state (T_2_) in close proximity of both S_1_ and T_1_, and improved SOC values compared to **2TPA-CNBT**. This would enable upconversion from T_1_ (CT)/T_2_ (CT/LE) to S_1_ (CT) through a combination of SOC and VC, possibly resulting in a good overall TADF performance.^58,60^

### 2.3 Photophysical Characterization

Steady-state absorption and emission spectra were measured in solution and doped films to experimentally probe the excited state properties. In toluene solution, very similar high-energy absorption bands are observed around 320 nm, attributed to the π–π* transition of the TPA moiety (Figure 5a).^37^ Meanwhile, CT absorption bands are observed around 515 or 535 nm, for **1TPA-(i)­CNBT** and **2TPA-(i)­CNBT**, respectively. The red-shift of this band in the disubstituted compounds logically results from the larger conjugated system, which lowers the energy of the excited states. The relative intensity of the CT bands varies significantly, showing the strongest CT absorption for **2TPA-CNBT**. This is in agreement with its calculated oscillator strength, which is nearly double that of the other emitters (Figures S6 and S7, and Table S2).

**Figure 5:**
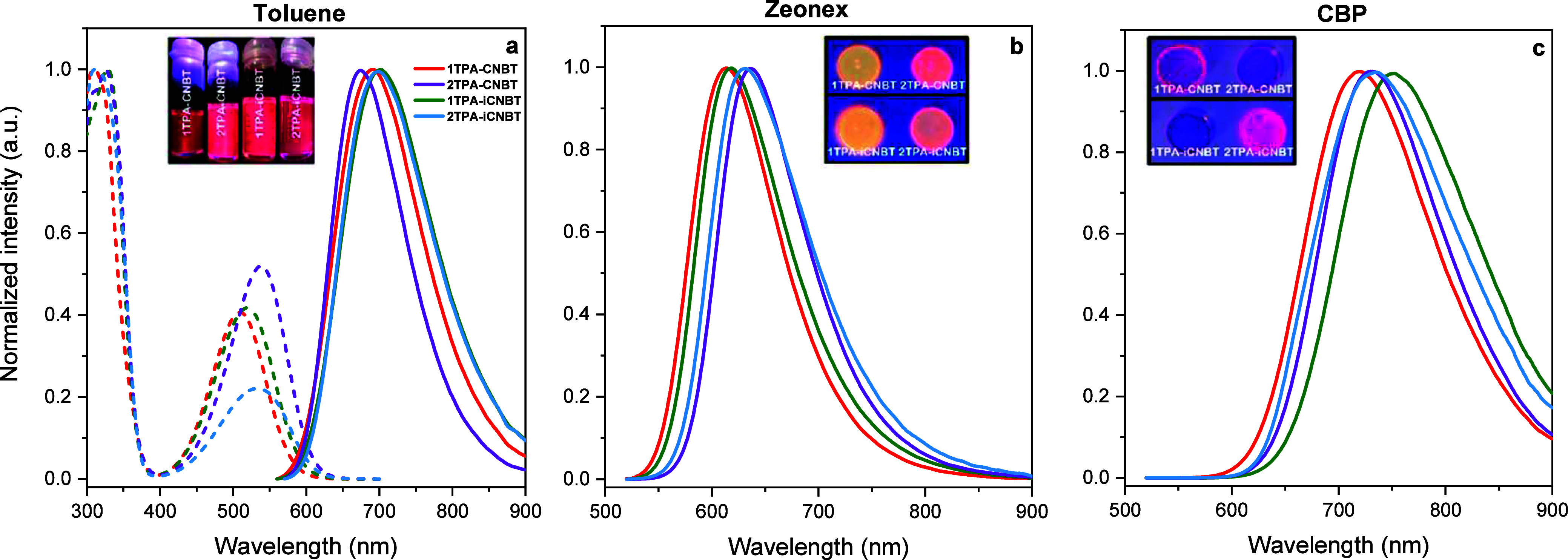
(a) Normalized steady-state absorption (dashed lines) and emission spectra (solid lines) for all four emitters in 10^–5^ M toluene solution (λ_exc_ = 550 nm) and normalized emission spectra for all four emitters in (b) 1 w/w % zeonex and (c) 10 w/w % CBP (λ_exc_ = 500 nm). Pictures show **1TPA-CNBT**, **2TPA-CNBT**, **1TPA-iCNBT**, and **2TPA-iCNBT** in solution and doped films under a 365 nm UV light source.

Meanwhile, the emission spectra in toluene are similar for each emitter, with exception of the slightly blue-shifted emission peak of **2TPA-CNBT** (Figure 5a). This is likely due to its overall weaker dipole moment (change) in the excited state—owing to its more centrosymmetric nature—resulting in weaker solvent stabilization of the emissive S_1_ state (Table S2). The CT nature of the emissive singlet state was further investigated through an analysis of the solvatochromic behavior in solvents with different polarities (Figure S9 and Table S3). The increased broadness and significantly red-shifted emission in the more polar solvents confirms the CT character of S_1_, as suggested by the TDDFT calculations.

Next, the photoluminescence quantum yields (PLQYs) were determined in toluene under inert (Φ_F__inert_) and ambient (Φ_F__air_) atmosphere (Table 1). Contrary to the excellent PLQY of **2TPA-CNBT** (Φ_F__air_ = 0.78), only moderate values were obtained for **1TPA-CNBT** (Φ_F__air_ = 0.27), **1TPA-iCNBT** (Φ_F__air_ = 0.30), and **2TPA-iCNBT** (Φ_F__air_ = 0.24). The intense fluorescence of **2TPA-CNBT** likely arises due to the limited triplet formation (vide infra), as indicated by the low SOC values between S_1_ and T_1_ (weak ISC). Surprisingly, no significant changes in the PLQY were observed for any of the emitters under an inert atmosphere, indicating subdued TADF behavior in toluene solution. While seemingly disagreeing with the previous observations of Kumsampao et al. (Φ_F__inert_ ≈ 1 for **2TPA-CNBT** in degassed toluene solution),^37^ poor TADF in solution might be expected, since the calculated Δ*E*_ST_ values are relatively large.

**Table 1:** Spectroscopic and Electrochemical Properties of the Four Emitters

compound	λ_abs,toluene_ (nm)^a^	λ_em,toluene_ (nm)^b^	λ_em,zeonex_ (nm)^c^	λ_em,CBP_ (nm)^c^	Φ_F,toluene_ ^d^ air/inert	Φ_F,zeonex_ ^e^ air/inert	Φ_Δ_ ^f^	HOMO/LUMO (eV)^g^	Δ*E*_ST,zeonex_ (eV)^h^
**1TPA-CNBT**	511	690	613	719	0.27/0.29	0.37/0.48	∼0.45	–5.58/–3.81	^i^
**2TPA-CNBT**	539	674	636	730	0.78/0.82	0.49/0.50	∼0.15	–5.58/–4.00	0.116
**1TPA-iCNBT**	516	701	618	752	0.30/0.33	0.57/0.63	∼0.45	–5.57/–3.80	0.143
**2TPA-iCNBT**	534	698	631	734	0.24/0.28	0.27/0.55	∼0.65	–5.54/–3.99	0.104

a CT band absorption maxima in toluene solution.

b Fluorescence emission maxima in 10^–5^ M toluene solution (λ_exc_ = 550 nm).

c Fluorescence emission maxima in zeonex (1 w/w %) and CBP (10 w/w %) film (λ_exc_ = 500 nm).

d Photoluminescence quantum yields in toluene solution under air and inert atmosphere, determined relatively vs Nile blue (Φ_F_ = 0.27, λ_exc_ = 550 nm in ethanol).

e Absolute photoluminescence quantum yields in zeonex determined using an integrating sphere under air and inert atmosphere at RT (λ_exc_ = 500 nm).

f Singlet oxygen quantum yields in toluene solution estimated vs Rose Bengal (Φ_Δ_ = 0.86, λ_exc_ = 525 nm in ethanol) by monitoring the absorbance of 1,3-diphenylisobenzofuran at 414 nm under emission from a secondary light source.

g Determined from cyclic voltammetry (Figure S10).

h The 80 ms delay-time spectra of **2TPA-CNBT**, **1TPA-iCNBT**, and **2TPA-iCNBT** contain multiple emission bands, therefore Δ*E*_ST_ was estimated from the difference between the peak wavelength maxima of the steady-state fluorescence band at RT and the 80 ms delay-time phosphorescence band at 80 K (Figure S11).

i Could not be unequivocally determined due to the nature of the 80 ms delay-time spectrum.

To further investigate the population of triplet states in (toluene) solution, the singlet oxygen quantum yield (Φ_Δ_) was measured under ambient conditions. An inverse trend was found for Φ_Δ_ versus Φ_F_, with **2TPA-CNBT** (Φ_Δ_ ≈ 0.15) exhibiting a relatively low singlet oxygen quantum yield against the higher values of **1TPA-CNBT** (Φ_Δ_ ≈ 0.45), **1TPA-iCNBT** (Φ_Δ_ ≈ 0.45), and especially **2TPA-iCNBT** (Φ_Δ_ ≈ 0.65). Weak ISC and subsequent limited triplet formation in **2TPA-CNBT** follows from the poor SOC between its S_1_ and T_1_ state, which has noticeably improved in **1TPA-CNBT** and **1TPA-iCNBT**. On the other hand, the ISC of **2TPA-iCNBT** is expected to be the strongest due to the presence of a hybrid triplet state, despite its moderate (<1.0) SOC value. As such, the higher singlet oxygen quantum yield of **2TPA-iCNBT** is indicative of the effective formation of (longer-lived) triplet states in solution.

Next, doped films of zeonex (1 w/w %) and CBP (10 w/w %) were prepared to investigate the photophysical behavior of the emitters in different solid-state hosts. Zeonex (a nonpolar polymer host) is often used to characterize the intrinsic emission behavior in film, without the influence of strong aggregation or secondary host effects (e.g. CT state stabilization and charge transport). Zeonex also has a low polarizability (comparable to methylcyclohexane),^61^ thus introducing only a marginal shift with respect to the gas-phase/isolated molecule emission. **2TPA-CNBT** and **2TPA-iCNBT** show slight bathochromic shifts in their zeonex emission compared to **1TPA-CNBT** and **1TPA-iCNBT** (Figure 5b), relating to their larger conjugated system and reduced singlet energies (Figure 4). At the same time, the PLQY and TADF contribution (based on the difference between Φ_F__air_ and Φ_F__inert_) steadily improve by moving from toluene to zeonex, with the notable exception of **2TPA-CNBT** (Table 1). **1TPA-iCNBT** shows an overall better performance than **1TPA-CNBT**, giving rise to a higher quantum yield under both ambient (Φ_F__air_ = 0.57 vs 0.37) and inert (Φ_F__inert_ = 0.63 vs 0.48) conditions. Surprisingly, similar quantum yields are achieved for **2TPA-iCNBT** (Φ_F__inert_ = 0.55) and **2TPA-CNBT** (Φ_F__inert_ = 0.50) under an inert atmosphere, despite their inherent differences in triplet harvesting. For **2TPA-CNBT**, this can be attributed to the high intrinsic PLQY, which is somewhat maintained in zeonex (Φ_F__air_ = 0.49). Meanwhile, the lower efficiency of **2TPA-iCNBT** under air (Φ_F__air_ = 0.27) is compensated by the stronger TADF properties, resulting in a doubling of the PLQY on removal of triplet-quenching oxygen. In CBP (a moderately nonpolar small-molecule host), a significant red-shift in the emission of all four emitters is observed (to >700 nm), owing to the stronger stabilization of the emissive CT state (Figure 5c). Interestingly, **1TPA-iCNBT** shows the longest photoluminescence wavelength, strongly contrasting the behavior of its isomeric counterpart **1TPA-CNBT**. However, this may also be attributed to a subtle difference in their aggregation behavior in conjunction with their higher concentrations in the CBP host, affecting the solid-state ordering and/or film quality of both emitters.

Time-resolved emission spectroscopy (TRES) experiments were carried out at room temperature (RT) and 80 K to investigate the delayed fluorescence behavior in doped films (Figures 6, 7, and S12–S14). TRES contour plots of the normalized emission spectra illustrate how the emission changes with respect to wavelength across different time scales, allowing the characterization of prompt (10^–9^–10^–7^ s) and delayed (10^–7^–10^–2^ s) fluorescence components. At RT, phosphorescence (10^–3^–10^3^ s) is typically outperformed by nonradiative decay (e.g. molecular vibrations) and therefore too weak to be observed. Hence, measurements were also performed at 80 K to limit vibrational energy losses and suppress TADF emission, enabling us to determine the experimental T_1_ energy and Δ*E*_ST_ (Table 1, Figure S11 and S14).

**Figure 6:**
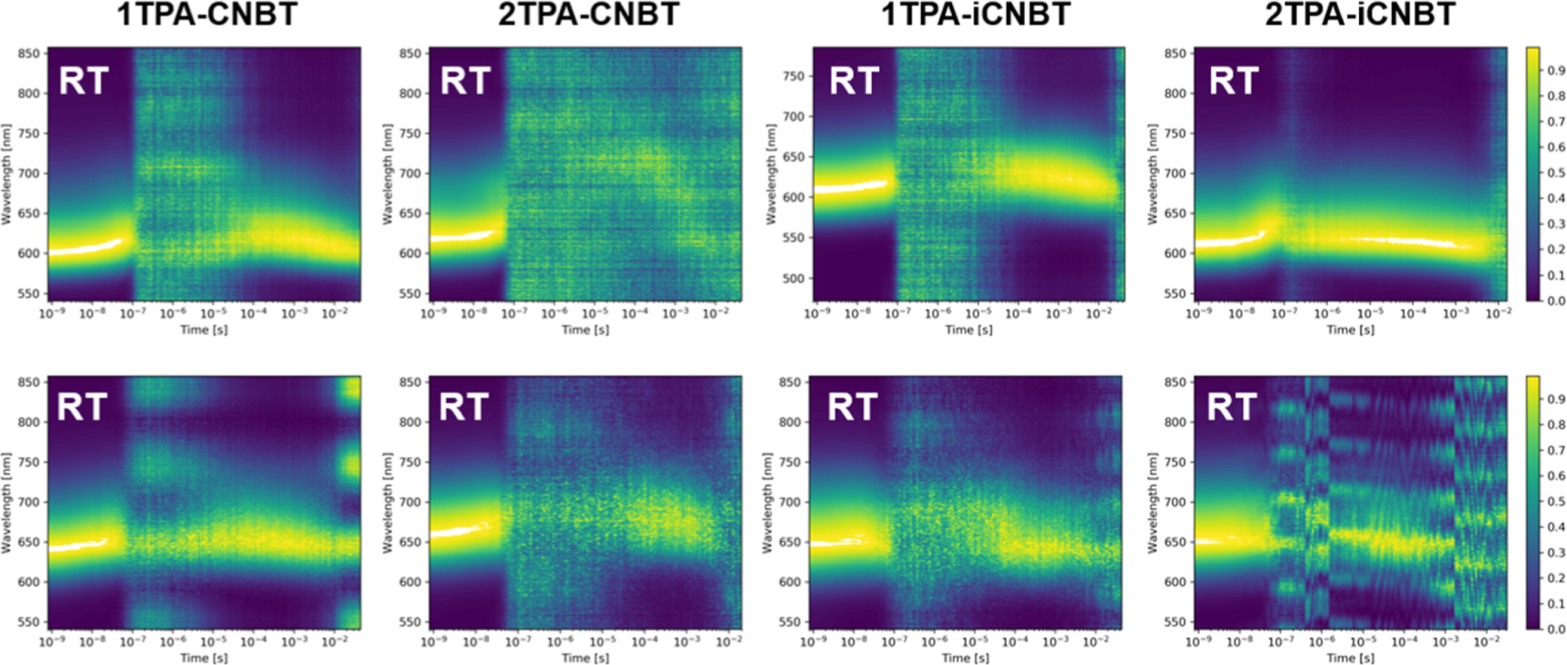
Normalized time-resolved emission spectra (contour plots) for all four isomers in 1 w/w % zeonex films (top row) and 10 w/w % CBP films (bottom row) at RT (λ_exc_ = 355 nm).

Prompt fluorescence with a similar intensity and lifetime (up to 100 ns) can be observed for each of the four emitters at RT in zeonex. A small red-shift can be seen toward the end of this time window, which is characteristic of slower decay from lower-energy singlet states of molecules in the film, which likely results from slightly altered dihedral angles between the donor and acceptor units. This shift intensifies at 80 K (Figure S14), as the rotational freedom of the TPA unit is further restricted.^62,63^ At RT, delayed fluorescence can be clearly observed (at the same wavelength as the prompt emission) for **1TPA-CNBT**, **1TPA-iCNBT**, and especially **2TPA-iCNBT**. The latter maintains a relatively strong delayed emission for most of the measurement (linked to its small Δ*E*_ST_ value), while the signal drops below the detection limit of the instrument during the early microsecond time window for **1TPA-CNBT** and **1TPA-iCNBT**. Meanwhile, **2TPA-CNBT** exhibits a delayed fluorescence contribution that barely rises above the instrument baseline at RT in zeonex. This TADF behavior aligns well with the improvement in PLQY under inert conditions, as previously discussed.

TRES measurements were similarly performed for doped CBP films at RT (Figure 6). While the captured delayed fluorescence intensity is weaker, likely due to the reduced thickness of the CBP films, there is a clearer progression in the quantity and kinetics of delayed emission for each material (Figure 7), with **2TPA-iCNBT** being the standout candidate emitter. To derive the kinetic parameters for **2TPA-iCNBT**, its decays were fitted using a kinetic model (Figure S15),^64^ which gave rate constants of 3.7 (2.8) × 10^7^ s^–1^, 5.4 (3.9) × 10^6^ s^–1^, and 0.45 (1.1) × 10^5^ s^–1^ for the rates of fluorescence, ISC, and RISC in zeonex (and CBP), respectively.

**Figure 7:**
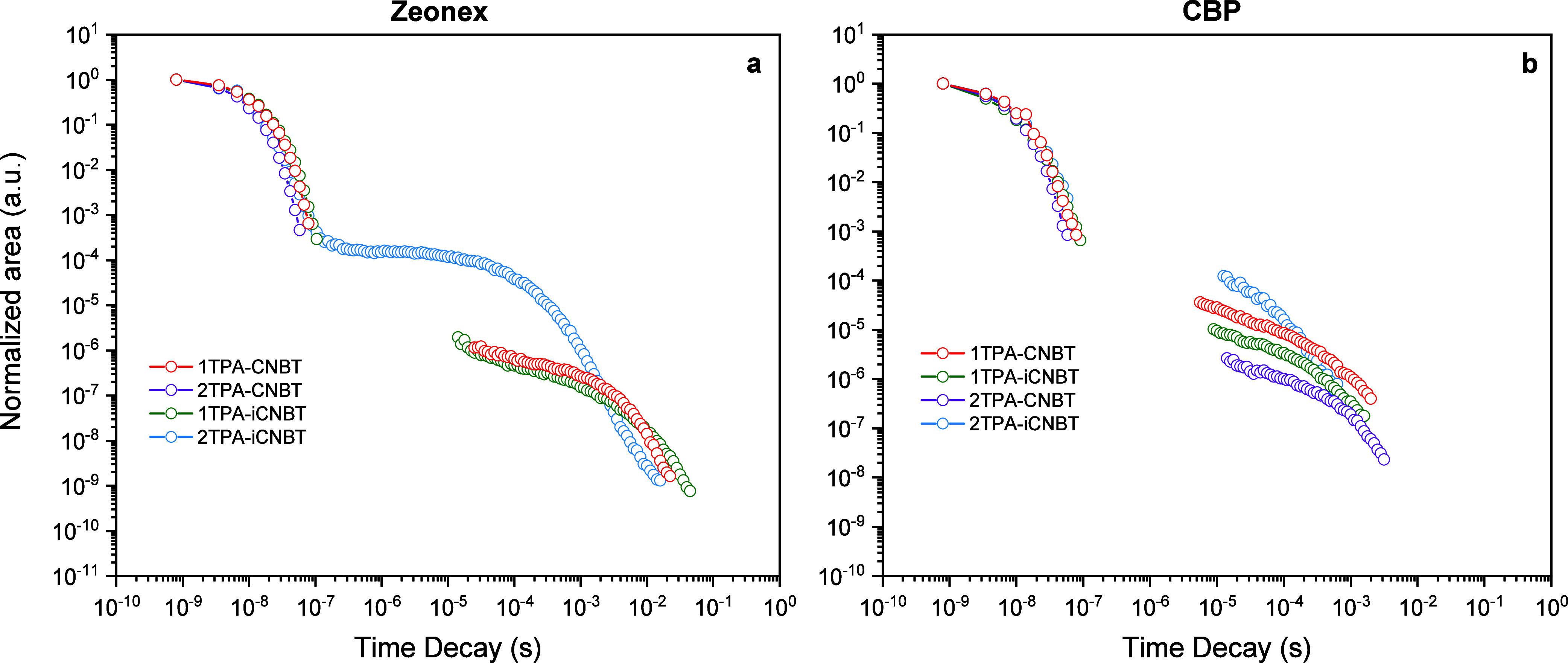
Total emission decay (calculated via the integrated area under the emission curve) for **1TPA-CNBT**, **2TPA-CNBT**, **1TPA-iCNBT**, and **2TPA-iCNBT** at RT in (a) 1 w/w % zeonex and (b) 10 w/w % CBP films. Data points with signal below the noise baseline (normalized to “flat” vertical traces in contour plots) have been omitted from the kinetics decay figures.

Despite the general similarities in the molecular structure and identical donor fragments, widely different emission behavior was established for each of the four emitters. **2TPA-CNBT** seemingly acts as a typical fluorescent dye, showcasing strong inherent fluorescence in solution and film but a limited capacity for TADF, in contrast with the reported literature.^37^ Interestingly, **1TPA-CNBT** demonstrates an overall better performance in film, exhibiting the second best TADF performance while maintaining a similar solid-state PLQY to **2TPA-CNBT**. Continuing, **2TPA-iCNBT** shows the largest increase in PLQY under an inert atmosphere and maintains the strongest delayed fluorescence intensity and fastest decays, clearly standing out as the best TADF emitter among the investigated compounds. This improved performance is likely supported by the close proximity of a hybrid ^3^LE/CT state which contributes to the VC (T_2_ in Figure 4), thereby promoting the RISC process.^59^ As for the CNBT-based emitters, the removal of one donor greatly impacts the capacity for TADF in terms of the Δ*E*_ST_ and delayed fluorescence intensity, as observed for **1TPA-iCNBT**.

### 2.4 OLED Device Fabrication and Characterization

Encouraged by the emissive decays and TADF properties recorded in CBP, solution-processed OLED devices of the four emitters were prepared. Following initial characterization and optimization efforts (Figures S16, S17, and Table S5), notable improvement in the device performance was achieved using a stack consisting of ITO/PEDOT:PSS/emitter:CBP (10 w/w %)/TmPyPB (60 nm)/LiF (1 nm)/Al (100 nm) as shown in Figure 8. Water-soluble poly­(3,4-ethylenedioxythiophene)/polystyrenesulfonate (PEDOT/PSS) was selected as a common hole-injection layer for solution-processed devices, since it exhibits good resistance to the organic solvent used during the following deposition of the emissive layer.^65^ In this case, the doped emissive layer was deposited by spin-coating (2500 rpm, 1 min) from toluene solution and subsequently annealed at 70 °C for 15 min. Finally, an electron-transporting/hole-blocking layer of 1,3,5-tri­[(3-pyridyl)-phen-3-yl]­benzene (TmPyPB), an electron-injection layer of LiF, and the Al cathode were deposited by thermal evaporation under vacuum. The electroluminescence (EL) spectra, EQE curves, and luminance versus voltage plots are shown in Figure 8 and the data are summarized in Table 2.

**Figure 8:**
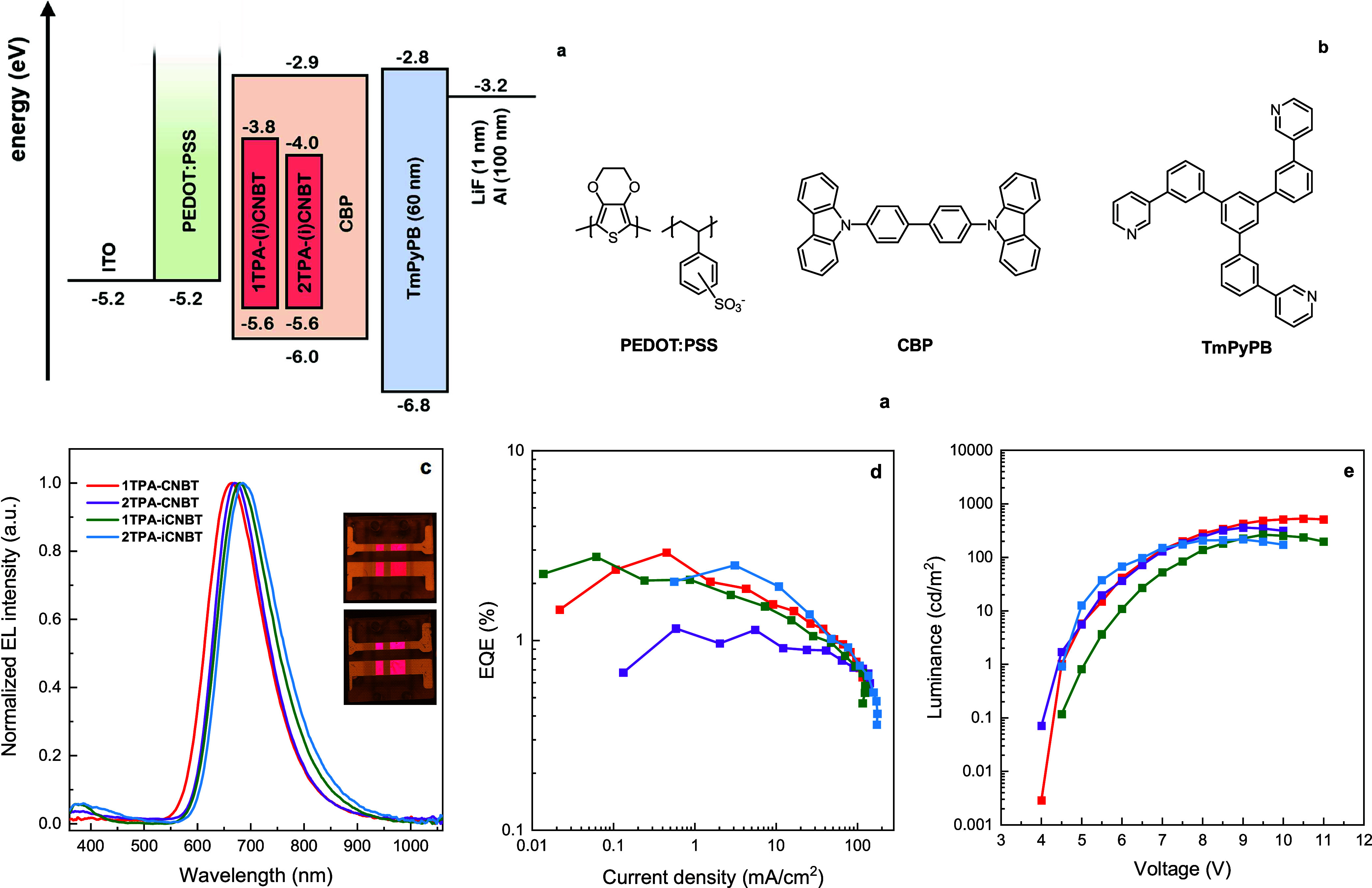
(a) Energy level diagram of the materials employed in the devices. (b) Molecular structures of the materials used in the devices. (c) EL spectra with photographs of the **1TPA-iCNBT** (top) and **2TPA-iCNBT** (bottom) solution-processed OLED devices. (d) EQE-current density curves, and (e) luminance versus voltage plots of the four doped (10 w/w % in CBP) SP-OLED devices.

**Table 2:** EL Characteristics of the Doped (10 w/w % in CBP) Solution-Processed OLEDs Based on the Four Compounds

device	λ_El,max_ (nm)^a^	EQE_max_ (%)^b^	EQE_10_ (%)^c^	*V*_on_ (V)^d^	*L*_max_ (cd·m^–2^)^e^
**1TPA-CNBT**	664	2.91	1.51	4.5	531 (10.5 V)
**2TPA-CNBT**	671	1.16	0.96	4.5	361 (9 V)
**1TPA-iCNBT**	679	2.76	1.41	4.5	265 (9.5 V)
**2TPA-iCNBT**	684	2.49	1.98	4.5	216 (9 V)

a Maximum electroluminescence peak.

b Maximum external quantum efficiency at 4.5–5 V.

c External quantum efficiency at a current density of 10 mA·cm^–2^.

d Turn-on voltage.

e Maximum luminance obtained at a specified voltage (given in parentheses).

Each of the devices displayed deep red and some NIR emission, with an EL peak centered between 664 and 684 nm. Interestingly, the EL spectra are notably blue-shifted compared to the PL spectra in CBP (Figure 5), which may arise from the difference in drying time and thickness between the spin-coated (rapidly dried) and drop-casted (slowly dried) films, respectively. Furthermore, a minor EL signal is found around 400 nm for all devices, which is attributed to the self-emission of the CBP host^66^ and/or TmPyPB.^67^ The generally low luminance values of the devices arise from the considerable portion of emission falling outside the human visible range (>700 nm) which is therefore not contributing to this metric, although the concurrent emission of significant visible light means that alternative reporting in radiosity units is of similarly limited usefulness.

Devices based on **1TPA-CNBT** exhibited the highest EQE_max_ (2.91%), closely followed by **1TPA-iCNBT** (2.76%), and **2TPA-iCNBT** (2.49%). In contrast, despite its intrinsically high PLQY (which is likely responsible for its comparably high luminance), **2TPA-CNBT** showed the worst EQE_max_ in these solution-processed OLEDs (1.16%). While this seemingly contradicts its reported OLED performance in evaporated devices, it is important to consider the generally inferior performance of solution-processed devices, and that maximum reported EQEs often correspond to the lowest-luminance measurements near the turn-on voltage (i.e. those measurements with the lowest signal-to-noise ratios).^68^ Nonetheless, the best overall EQE at moderate current densities (∼10 mA) within this series was maintained by **2TPA-iCNBT**, consistent with its higher delayed emission intensity and faster TADF kinetics in the optical measurements of CBP films. For the other emitters which exhibit slower RISC, the accumulation of triplet excitons more rapidly enables multiexciton quenching processes, leading to a more severe loss of efficiency as the device is driven with higher currents. While there is plenty of room (and need) for further improvements—especially in terms of the NIR emission wavelength range, EQE, and stability—these results contribute to the growing interest in solution-processed NIR-TADF OLEDs, where reported examples remain relatively scarce (Table S6).^10,69,70^

## 3 Conclusions

The influence of the relative position and number of triphenylamine donor units on the (delayed) emission properties of **TPA-(i)­CNBT** was investigated, revealing considerable changes in the emission wavelength, photoluminescence quantum yield, delayed fluorescence rate, TADF efficiency, and solution-processed OLED performance. **2TPA-CNBT**, chosen as the reference compound, showed modest TADF performance. In comparison, the Y-shaped emitter **2TPA-iCNBT** exhibited superior TADF behavior in the solid state, likely owing to its larger dihedral angle between the closely spaced donor units, and the introduction of the **iCNBT** acceptor core which generates close-lying hybrid CT/LE triplet states. At the same time, the TADF behavior changed drastically when one of the donor units was removed. **1TPA-iCNBT** showed a largely reduced TADF performance (and a higher, less oxygen-sensitive PLQY) compared to **2TPA-iCNBT**, which is attributed to a reduced dihedral angle, a larger Δ*E*_ST_, and the absence of any apparent LE or hybrid triplet states. Meanwhile, a rather unexpected but significant TADF contribution was found for **1TPA-CNBT**, in clear opposition to **2TPA-CNBT**. Despite exhibiting a similar calculated dihedral angle and simulated Δ*E*_ST_ to **2TPA-CNBT**, the removal of one donor unit greatly improves the SOC between the S_1_ and T_1_ excited states of **1TPA-CNBT**, yielding a comparable performance to **1TPA-iCNBT**. All four emitters were prepared in high yields using a direct arylation strategy, enabling the development of previously unattainable TADF scaffolds. In the end, deep-red to near-infrared solution-processed doped OLEDs of **1TPA-CNBT**, **1TPA-iCNBT**, and **2TPA-iCNBT** gave rise to modest EQE_max_ values of 2.91, 2.76, and 2.49%, respectively, with the latter maintaining the highest efficiency at higher driving currents (>10 mA cm^–2^) and lowest roll-off within the series. These results demonstrate the importance of investigating varying structural motifs, substitution positions, and especially the role of auxiliary donor units in the development of new TADF emitters, ensuring proper exploration of the underlying design rules and the discovery of more performant isomeric variants.

## Supplementary Material



## Notes

Synthetic procedures, (TD)­DFT calculations, simulated spectra, analyses of the electronic excitations, additional (time-resolved) absorption and emission spectra, cyclic voltammetry data, device optimization overview, and structural characterization (NMR and MALDI-ToF mass spectrometry) (PDF)
