# Supplementary material for: A Direct Arylation Approach toward Thermally Activated Delayed Fluorescence-Active Benzo[c][1,2,5]thiadiazole Emitters for Near-Infrared Solution-Processed OLEDs
Source: ACS Appl Opt Mater. 2025 Oct 20;3(11):2583–94. doi: 10.1021/acsaom.5c00340 (PMC12671064; doi:10.1021/acsaom.5c00340)
Supplement: Supplementary file 1 [file ot5c00340_si_001.pdf]

Supporting information

A Direct Arylation Approach toward Thermally Activated Delayed Fluorescence-Active Benzo[*c*][1,2,5]thiadiazole Emitters for Near-Infrared Solution-Processed OLEDs

Sonny Brebels,*[a,b] Emma V. Puttock,[c] Tom Cardeynaels,[a,b,d] Kamile Bareikaite,[c] Lucy A. Weatherill,[c] Melissa Van Landeghem,[a,b] Huguette Penxten,[a,b] Andrew Danos,*[c, e] Koen Vandewal,[a,b] Andrew P. Monkman,[c] Benoît Champagne[d] and Wouter Maes*[a,b]

[a] Hasselt University, Institute for Materials Research (IMO-IMOMEC), Martelarenlaan 42, B-3500 Hasselt, Belgium; E-mail: sonny.brebels@uhasselt.be, wouter.maes@uhasselt.be
[b] IMOMEC Division, IMEC, Wetenschapspark 1, B-3590 Diepenbeek, Belgium
[c] Durham University, Department of Physics, OEM group, South Road, Durham DH1 3LE, United Kingdom
[d] University of Namur, Laboratory of Theoretical Chemistry, Theoretical and Structural Physical Chemistry Unit, Namur Institute of Structured Matter, Rue de Bruxelles 61, B-5000 Namur, Belgium
[e] Queen Mary University of London, School of Physical and Chemical Sciences, 327 Mile End Road, London E1 4NS, United Kingdom; E-mail: a.danos@qmul.ac.uk

Table of contents

1. Materials and methods S2
2. Materials synthesis S3
3. TDDFT calculations S5
4. Absolute absorption spectra and singlet oxygen quantum yields in toluene S11
5. Solution state absorption and emission spectra for solvatochromism determination S12
6. Cyclic voltammetry and HOMO/LUMO determination S13
7. Energy gap determination from steady-state fluorescence and phosphorescence spectra S14
8. Normalized time-resolved emission spectra S15
9. Normalized time-resolved emission contour plots and kinetic fitting of the emission decay S17
10. Device optimization S18
11. Comparative electroluminescence performance S20
12. NMR & MALDI-ToF mass spectra S21
13. Coordinates of optimized geometries S32
14. References S36

1. Materials and methods

All commercially available reagents were obtained from Acros Organics, Alfa Aesar, BLD Pharmatech, Fluorochem, J&K Scientific, Sigma-Aldrich, STREM Chemicals, TCI Europe, or VWR Chemicals and were used without further purification. Solvents were obtained from Fisher Scientific, Sigma-Aldrich, or VWR Chemicals and were used without further purification. Dry solvents were obtained from an MBraun solvent purification system (MB SPS-800) equipped with alumina columns. Preparative (recycling) size exclusion chromatography (SEC) was performed on a JAI LC-9110 NEXT system equipped with JAIGEL 1H and 2H columns (eluent chloroform, flow rate 3.5 mL min^{-1}) or a JAI LaboACE LC-7080 Plus system with JAIGEL 2HR and 2.5HR columns (eluent chloroform, flow rate 10 mL min^{-1}). Proton, carbon, and fluorine nuclear magnetic resonance (^{1}H, ^{13}C, and ^{19}F NMR) spectra were obtained on a Jeol spectrometer operating at 400 MHz for ^{1}H (100 MHz for ^{13}C, 375 MHz for ^{19}F). Chemical shifts (δ) are given in ppm relative to $CDCl_3$ (δ = 7.26 ppm for ^{1}H NMR, δ = 77.16 ppm for ^{13}C NMR), DMSO-d_6 (δ = 2.50 ppm for ^{1}H NMR, δ = 39.52 ppm for ^{13}C NMR) or $CFCl_3/CDCl_3$ (δ = 0 ppm for ^{19}F). Coupling constants are given in Hz. Matrix-assisted laser desorption/ionization - time-of-flight (MALDI-ToF) mass spectra were recorded on a Bruker Daltonics Ultraflex II Tof/Tof. To prepare the MALDI samples, 1 µL of the matrix solution (16 mg mL^{-1} *trans*-2-[3-(4-*tert*-butylphenyl)-2-methyl-2-propenylidene]malononitrile or DTCB in chloroform) was spotted onto an MTP Anchorchip 600/384 MALDI plate. The spot was allowed to dry and 1 µL of the analyte solution (0.5 mg mL^{-1} in chloroform) was spotted on top of the matrix.

Electronic absorption spectra of the small molecule chromophores in solution were recorded on a Varian Cary 5000 UV-Vis-NIR spectrophotometer from Agilent Technologies. Corrected steady-state excitation and emission spectra in solution were recorded on an Edinburgh FLS-1000 spectrofluorometer equipped with a 450 W Xe lamp as light source, with the excitation wavelength (λ_{exc}) depending on the fluorescence quantum yield standard used. Corrected steady-state excitation and emission spectra of thin films were recorded on a Horiba-Jobin Yvon Fluorolog-3 spectrofluorometer equipped with a 450 W Xe lamp as the light source, with an excitation wavelength (λ_{exc}) depending on the fluorescence quantum yield standard used. Freshly prepared samples in 1 cm quartz cells were used to perform all UV-Vis-NIR absorption and emission measurements. The fluorescence measurements were done under a right-angle arrangement. The standard uncertainty (square root of the variance) on the absorption and emission maxima is approximately 1 nm. Spectroscopic measurements under normal atmosphere were done in non-degassed spectroscopic grade solvents at 20 °C. Inert atmosphere was created by three consecutive freeze-pump-thaw cycles.

For the determination of the relative fluorescence quantum yields (Φ_F) in toluene, dilute solutions with an absorbance around 0.1 at the excitation wavelength were used. Nile blue (λ_{exc} = 550 nm, Φ_F = 0.27 in ethanol) was used as a standard to determine the fluorescence quantum yields [1]. The fluorescence quantum yield of the tested compound (Φ_x) was calculated using **Equation S1**, in which Φ_{st} is the fluorescence quantum yield of the standard, F_x and F_{st} are the integrated fluorescence of the test compound and the standard, A_x and A_{st} are the absorbance of the test compound and the standard at the excitation wavelength, and n_x and n_{st} are the refractive indices of the solvents in which the test compound and the standard were dissolved, respectively.

$$\Phi_x = \Phi_{st}\frac{F_x A_{st} n_x^2}{F_{st} A_x n_{st}^2} \quad \textbf{(S1)}$$

1,3-Diphenylisobenzofuran (1,3-DPBF) was used as a singlet oxygen ($^1O_2$) scavenger to determine the singlet oxygen quantum yields (Φ_Δ). The $^1O_2$ production was monitored by following the absorbance of 1,3-DPBF at 414 nm upon excitation of the respective chromophore at 525 nm using a single LED325W2 from Thorlabs (λ_{exc} = 525±5 nm, fwhm = 11 nm, P = 1.7 mW). To determine Φ_Δ, a relative method was used according to **Equation S2**. Here, '*x*' and '*st*' represent the sample and the standard, while Φ, A, m, and n represent the singlet oxygen quantum yield, the absorbance at the excitation wavelength (λ_{exc} = 525 nm), the slope of the decrease in absorbance of 1,3-DPBF over time, and the refractive index of the solvent used for the measurement, respectively. Optically matched solutions with an absorbance around 0.6 at 414 nm and 0.3 at 325 nm were used. Rose Bengal was used as the standard (Φ_Δ = 0.86 in spectrograde ethanol) [2] The solutions were continuously stirred during all measurements using a Cimarec magnetic stirrer.

$\Phi_x = \Phi_{st}$ [illegible] (**S2**)

Zeonex films were prepared via drop-casting using a mixture of the emitter and host (zeonex) in toluene at 1 w/w%. The initial solution concentrations consisted of 100 mg mL^{-1} of zeonex and 1 mg mL^{-1} of emissive material. 4,4′-Bis(*N*-carbazolyl)-1,1′-biphenyl (CBP) films were prepared via drop-casting using a mixture of the emitter and host (CBP) in toluene at 10 w/w%. The initial solution concentrations consisted of 10 mg mL^{-1} of CBP and 1 mg mL^{-1} of emissive material. The films were drop-casted onto a quartz substrate at 50 °C to facilitate evaporation of the solvent. Time-resolved photoluminescence spectra and decays of the thin film samples were recorded using a nanosecond gated spectrograph-coupled iCCD (Stanford) using an Nd:YAG laser emitting at 355 nm (EKSPLA) under vacuum. Photoluminescence quantum yields in film were determined using a calibrated integrating sphere (Horiba Quanta-Phi) fibre-coupled to a spectrofluorometer (Horiba Fluorolog) as excitation source (450 nm) and detection system. For the measurements in inert atmosphere, the sphere was flushed with a stream of dry nitrogen for at least 30 min to prevent oxygen quenching of triplets.

Cyclic voltammetry (CV) was carried out in argon-purged acetonitrile at room temperature with an Autolab potentiostat (PGSTAT30 from Metrohm) controlled with GPES software (version 4.9) for data collection and analysis. Tetrabutylammonium hexafluorophosphate ($TBAPF_6$) (0.1 M) was used as the supporting electrolyte. A one-compartment microcell designed for three-electrode configuration was fitted with a platinum wire working electrode, a platinum wire counter electrode, and an $Ag/AgNO_3$ reference electrode (Ag wire immersed in electrolyte containing 0.01 M $AgNO_3$). A film of the analyte was deposited on the working electrode by casting it from a chlorobenzene solution. Voltammograms were recorded at a scan rate of 100 mV s^{-1} under a constant flow of argon, allowing degassing and blanketing of the electrolyte before and during analysis. 5 scans per film were recorded and the highest occupied/lowest unoccupied molecular orbital (HOMO/LUMO) energy levels were estimated from the averaged onset potentials of the 3rd, 4th, and 5th scan. The onset potential was determined from the intersection of two tangents drawn at the rising and background current of the cyclic voltammogram. Ferrocene was used as an external standard. For the conversion of potentials (in V) obtained from the electrochemical measurements to frontier molecular orbital energies (in eV), the formal potential of the Fc/Fc^+ redox couple was scaled to the vacuum level with a value of –4.98 eV, considering that 0.0 V on the ferrocene scale corresponds to 0.31 V versus SCE (0.55 V versus SHE) and that 0.0 V versus SHE is equivalent to –4.44 eV (**Equation S3**) [3, 4].

$$\text{HOMO/LUMO (eV)} = -4.98\ (\text{eV}) - E_{onset}\ (\text{analyte})\ \text{vs}\ Ag/AgNO_3\ (\text{V}) + E_f\ (Fc/Fc^+)\ \text{vs}\ Ag/AgNO_3\ (\text{V}) \qquad \textbf{(S3)}$$

OLEDs were fabricated on patterned indium-tin-oxide-coated glass (VisionTek Systems) with a sheet resistance of 15 Ω sq^{-1}. After sonicating in acetone and isopropanol, substrates were oxygen-plasma-cleaned and the hole injection layer (PEDOT:PSS) and emitting layer were spin-coated. PEDOT:PSS was filtered with a 0.45 µm PES syringe filter, spin-coated at 1000 rpm for 10 s, then at 4000 rpm for 60 s, and annealed at 120 °C for 15 mins. The compounds for the emissive layer and the CBP host were dissolved in toluene (1 and 10 mg/mL, respectively, to achieve a total 10 wt% concentration of the mixture), filtered using a 0.45 µm PTFE syringe filter, spin-coated on the PEDOT:PSS layer at 2500 rpm for 60 s, and annealed at 70 °C for 15 mins. The substrates were then loaded into a Kurt J. Lesker Super Spectros deposition chamber, where the electron transport/hole blocking layer (TmPyPB) and cathode (LiF/Al) were thermally evaporated at pressures below 10^{-7} mbar. Freshly evaporated devices were one by one transferred into a calibrated 10-inch integrating sphere (Labsphere), and their electrical properties were measured using a source meter (Keithley 2400). Emission spectra were simultaneously measured using a calibrated fiber-coupled QePro spectrometer (Ocean Optics). Devices were evaluated at 298 K and under air atmosphere.

2. Materials synthesis

4,4'-(5,6-difluorobenzo[c][1,2,5]thiadiazole-4,7-diyl)bis(N,N-diphenylaniline) ***(2)***

To a stirred mixture of (4-(diphenylamino)phenyl)boronic acid (272 mg, 0.94 mmol), 4,7-dibromo-5,6-difluorobenzo[*c*][1,2,5]thiadiazole (132 mg, 0.40 mmol) and $Pd(PPh_3)_4$ (23 mg, 0.02 mmol) in dry toluene (55 mL) and anhydrous ethanol (25 mL), an aqueous K_2CO_3 solution (2 M, 20 mL) was added under argon. The reaction mixture was heated to reflux and stirred for 48 h. After cooling to room temperature, the solvent was removed under vacuum. After extracting with dichloromethane, washing with brine solution, drying over anhydrous $MgSO_4$, and filtration of the drying

agent, the solvent was removed under reduced pressure. The crude product was purified by column chromatography on silica gel (eluent: petroleum ether/dichloromethane = 6:4, v/v) to give **2** as an orange solid (234 mg, 88%). ^{1}H NMR (400 MHz, Chloroform-*d*) δ 7.72 (d, *J* = 8.8 Hz, 4H), 7.35 – 7.27 (m, 8H), 7.23 – 7.16 (m, 12H), 7.13 – 7.04 (m, 4H). ^{13}C NMR (100 MHz, Chloroform-*d,* ^{19}F decoupled) δ 150.7, 150.4, 148.6, 147.3, 131.4, 129.6, 125.4, 123.8, 123.3, 121.9, 117.9. ^{19}F NMR (375 MHz, Chloroform-*d*) δ -133.63. MALDI-ToF MS (*m*/*z*): Calcd. for $C_{42}H_{28}F_2N_4S$ $[M]^+$: *m*/*z* 658.20 (100%), found: 658.23.

*4,7-bis(4-(diphenylamino)phenyl)benzo[c][1,2,5]thiadiazole-5,6-dicarbonitrile (**3**)*

General procedure 1: To a mixture of **2** (100 mg, 0.15 mmol), KCN (60 mg, 0.91 mmol) and 18-crown-6 (4 mg, 0.015 mmol), flushed with argon for 5 min, dry THF (10 mL) was added. The mixture was refluxed for 24 h. After cooling to room temperature, the solvent was removed under vacuum. After extracting with dichloromethane, washing with water, drying over anhydrous Na_2SO_4, and filtration of the drying agent, the solvent was removed under reduced pressure. The crude product was purified by column chromatography on silica gel (eluent: petroleum ether/dichloromethane = 7:3 to 5:5, v/v) to give **3a** as a black solid (4 mg, 4%).

Altered procedure 1b: Similar to general procedure 1, with a reaction time of 48 h. Since conversion remained low, an additional amount of KCN (60 mg, 0.91 mmol) and 18-crown-6 (4 mg, 0.015 mmol) was added and the mixture was left to reflux for another 48 h. Performing the same work-up as before yielded **3b** as a black solid (10 mg, 9%). ^{1}H NMR (400 MHz, Chloroform-*d*) δ 7.68 (d, *J* = 8.3 Hz, 4H), 7.36 – 7.28 (m, 8H), 7.24 – 7.19 (m, 8H), 7.20 – 7.09 (m, 8H). MALDI-ToF MS (*m*/*z*): Calcd. for $C_{44}H_{28}N_6S$ $[M]^+$: *m*/*z* 672.21 (100%), found: 672.44.

*benzo[c][1,2,5]thiadiazole-5,6-dicarbonitrile (**5**)*

General procedure 2: A solution of 5,6-dibromobenzo[*c*][1,2,5]thiadiazole (1.47 g, 5 mmol) in dry NMP (25 mL) was heated to 100 °C, after which CuCN (1.60 g, 18 mmol) was added. The mixture was further heated to 170 °C and left to react for 2 h. After cooling to room temperature, the reaction was quenched by the addition of ammonia solution (15 v/v%). After extracting with dichloromethane (3x) and ethyl acetate (1x), washing with water, drying over anhydrous $MgSO_4$, and filtration of the drying agent, the solvent was removed under reduced pressure. The crude product was purified by column chromatography on silica gel (eluent: dichloromethane) to give **5** as a white solid (0.62 g, 66%). ^{1}H NMR (400 MHz, Chloroform-*d*) δ 8.60 (s, 2H). ^{13}C NMR (100 MHz, Chloroform-*d*) δ 159.4, 135.2, 120.0, 119.4.

*benzo[c][1,2,5]thiadiazole-4,7-dicarbonitrile (**9**)*

Synthesis according to general procedure 2: 4,7-dibromobenzo[*c*][1,2,5]thiadiazole (2.90 g, 10 mmol), NMP (50 mL), CuCN (3.20 g, 36 mmol); light brown solid (1.90 g, quantitative). ^{1}H NMR (400 MHz, Chloroform-*d*) δ 8.12 (s, 2H). ^{13}C NMR (100 MHz, Chloroform-*d*) δ 152.5, 134.5, 113.9, 110.8.

*4,7-bis(4-(diphenylamino)phenyl)benzo[c][1,2,5]thiadiazole-5,6-dicarbonitrile (**2TPA-CNBT**, **6**) and 4-(4-(diphenylamino)-phenyl)benzo[c][1,2,5]thiadiazole-5,6-dicarbonitrile (**1TPA-CNBT**, **7**)*

General procedure 3: A stirred mixture of **5** (0.093 g, 0.5 mmol), 4-bromo-*N*,*N*-diphenylaniline (0.357 g, 1.1 mmol), $Pd(OAc)_2$ (0.011 g, 0.05 mmol), P*t*Bu_2Me.HBF_4 (0.025 g, 0.1 mmol), K_2CO_3 (0.207 g, 1.5 mmol), and pivalic acid (0.051 g, 0.5 mmol) in dry toluene (3 mL) was refluxed under argon for 24 h. After cooling to room temperature, the solvent was removed under reduced pressure. The crude product was purified by column chromatography on silica gel (eluent: petroleum ether/dichloromethane = 5:5, v/v) to give **6a** as a purple solid (338 mg, quantitative).

Altered procedure 3b: Synthesis according to general procedure 3 with a reduced number of equivalents of 4-bromo-*N*,*N*-diphenylaniline. **5** (0.093 g, 0.5 mmol), 4-bromo-*N*,*N*-diphenylaniline (0.178 g, 0.55 mmol), $Pd(OAc)_2$ (0.006 g, 0.025 mmol), P*t*Bu_2Me.HBF_4 (0.012 g, 0.05 mmol), K_2CO_3 (0.104 g, 0.75 mmol), pivalic acid (0.026 g, 0.25 mmol), dry toluene (3 mL); purple solid (**6b**, 150 mg, 45%) and red solid (**7b**, 100 mg, 47%) isolated. Compounds **6** and **7** were further purified by preparative (recycling) size exclusion chromatography.

Compound **6**: ^{1}H NMR (400 MHz, Chloroform-*d*) δ 7.68 (d, *J* = 8.8 Hz, 4H), 7.40 – 7.27 (m, 8H), 7.24 – 7.21 (m, 8H), 7.20 – 7.09 (m, 8H). ^{13}C NMR (100 MHz, Chloroform-*d*) δ 154.6, 150.2, 146.7, 140.6, 131.6, 129.7, 126.1, 124.7, 124.6, 120.4, 116.3, 111.6. MALDI-ToF MS (*m*/*z*): Calcd. for $C_{44}H_{28}N_6S$ $[M]^+$: *m*/*z* 672.21 (100%), found: 672.21. Mp: 322-323 °C (323 °C reported) [5].

Compound **7**: ^{1}H NMR (400 MHz, Chloroform-*d*) δ 8.43 (s, 1H), 7.68 (d, *J* = 8.8 Hz, 2H), 7.41 – 7.27 (m, 4H), 7.24 – 7.21 (m, 4H), 7.19 – 7.09 (m, 4H). ^{13}C NMR (100 MHz, Chloroform-*d*) δ 154.4, 154.3, 150.6, 146.6, 143.4, 131.7, 129.8, 127.0,

126.2, 124.8, 124.1, 120.2, 115.73, 115.69, 115.6, 109.6. MALDI-ToF MS (*m/z*): Calcd. for $C_{26}H_{15}N_5S$ $[M]^+$: *m/z* 429.10 (100%), found: 429.15. Mp: 237-238 °C.

5,6-bis(4-(diphenylamino)phenyl)benzo[c][1,2,5]thiadiazole-4,7-dicarbonitrile ***(2TPA-iCNBT*, 10)** *and 5-(4-(diphenyl-amino)phenyl)benzo[c][1,2,5]thiadiazole-4,7-dicarbonitrile* ***(1TPA-iCNBT*, 11)**

Synthesis according to general procedure 3: **9** (0.093 g, 0.5 mmol), 4-bromo-*N*,*N*-diphenylaniline (0.357 g, 1.1 mmol), $Pd(OAc)_2$ (0.011 g, 0.05 mmol), $PtBu_2Me.HBF_4$ (0.025 g, 0.1 mmol), K_2CO_3 (0.207 g, 1.5 mmol), pivalic acid (0.051 g, 0.50 mmol), dry toluene (3 mL); purple solid (**10**, 159 mg, 47%) and red solid (**11**, 99 mg, 46%) isolated. Compounds **10** and **11** were further purified by preparative (recycling) size exclusion chromatography.

Compound **10**: ^{1}H NMR (400 MHz, Chloroform-*d*) δ 7.33 – 7.26 (m, 8H), 7.17 – 7.04 (m, 12H), 6.98 (s, 8H). ^{13}C NMR (100 MHz, Chloroform-*d*) δ 152.4, 150.5, 149.1, 146.9, 131.2, 129.7, 127.5, 125.6, 124.2, 121.0, 114.6, 108.5. MALDI-ToF MS (*m/z*): Calcd. for $C_{44}H_{28}N_6S$ $[M]^+$: *m/z* 672.21 (100%), found: 672.39. Mp: 275-277 °C.

Compound **11**: ^{1}H NMR (400 MHz, Chloroform-*d*) δ 8.23 (s, 1H), 7.57 (d, *J* = 8.8 Hz, 2H), 7.43 – 7.26 (m, 4H), 7.23 – 7.10 (m, 8H). ^{13}C NMR (100 MHz, Chloroform-*d*) δ 154.4, 150.8, 150.6, 148.1, 146.5, 137.7, 130.2, 129.8, 126.5, 126.0, 124.9, 121.1, 114.9, 114.2, 109.6, 105.3. MALDI-ToF MS (*m/z*): Calcd. for $C_{26}H_{15}N_5S$ $[M]^+$: *m/z* 429.10 (100%), found: 429.11. Mp: 197-200 °C.

3. TDDFT calculations

Table S1. Nature of the various transitions (H = HOMO, L = LUMO), charge-transfer distance (d_{CT}), and change in dipole moment ($\Delta\mu$, excited state dipole – ground state dipole) accompanying the $S_0 \rightarrow S_x$ (x = 1,2) and $S_0 \rightarrow T_x$ (x = 1,2,3) transitions in methylcyclohexane, as determined with TDDFT-TDA and a modified LC-BLYP (ω = 0.17 bohr^{-1}) exchange correlation functional. Values for the Earth Mover's charge-transfer distance ($^{EM}d_{CT}$, given in italics) are calculated via the method of Fraiponts *et al.* [6]

Compound	$S_0 \rightarrow S_1$				$S_0 \rightarrow S_2$				$S_0 \rightarrow T_1$				$S_0 \rightarrow T_2$				$S_0 \rightarrow T_3$		
	Nature	d_{CT} (Å)[a]	$\Delta\mu$ (D)[b]	q_{CT}[c]	Nature	d_{CT} (Å)[a]	$\Delta\mu$ (D)[b]	q_{CT}[c	Nature	d_{CT} (Å)[a]	$\Delta\mu$ (D)[b]	q_{CT}[c]	Nature	d_{CT} (Å)[a]	$\Delta\mu$ (D)[b]	q_{CT}[c]	Nature	d_{CT} (Å)[a]	$\Delta\mu$ (D)[b]
**1-TPA-CNBT**	H→L (89%)	4.18 *(5.20)*	19.01	0.95	H→L+1 (65%) H→L+2 (7%)	4.13 *(5.15)*	15.22	0.77	H→L (67%) H-1→L (19%)	3.77 *(5.27)*	13.77	0.76	H-1→L (26%) H-7→L (18%) H→L (13%) H-3→L (13%)	3.25 *(4.83)*	6.89	0.44	H-6→L (43%) H-7→L (29%)	1.19 *(2.05)*	2.26
**2-TPA-CNBT**	H→L (88%)	0.60 *(4.97)*	2.50	0.87	H-1→L (89%)	0.50 *(4.55)*	2.23	0.93	H→L (67%) H-2→L (25%)	0.77 *(4.90)*	2.40	0.65	H-1→L (75%)	0.32 *(4.44)*	1.40	0.90	H-2→L (36%) H-13→L (19%) H→L (15%)	0.53 *(4.89)*	1.25
**1-TPA-iCNBT**	H→L (91%)	4.67 *(5.84)*	21.23	0.95	H-1→L (47%) H-2→L (17%) H-5→L (10%)	3.42 *(4.63)*	6.79	0.41	H→L (66%) H-1→L (21%)	4.28 *(5.69)*	14.97	0.73	H-5→L (28%) H-1→L (17%) H→L (14%) H-6→L (13%)	2.79 *(3.71)*	5.35	0.40	H-7→L (33%) H→L+2 (22%) H-5→L (10%)	1.83 *(2.94)*	4.00
**2-TPA-iCNBT**	H→L (90%)	3.76 *(4.25)*	17.16	0.95	H-1→L (91%)	3.96 *(4.44)*	19.49	1.03	H→L (80%)	3.63 *(4.26)*	16.13	0.93	H-1→L (45%) H-3→L (34%)	3.34 *(3.39)*	8.39	0.52	H-1→L (39%) H-3→L (20%) H-6→L (15%)	3.68 *(4.60)*	11.33

[a] Distance over which charge is transferred between the indicated states upon excitation. [b] Dipole moment change upon excitation. [c] Amount of charge transferred.

Table S2. TDDFT results for the two vertical singlet excitation energies and the corresponding oscillator strengths (*f*), and the three vertical triplet excitation energies as determined with TDDFT-TDA and a modified LC-BLYP (ω = 0.17 bohr^{-1}) exchange correlation functional, and the calculated singlet-triplet and triplet-triplet energy gaps between the specified (first) singlet and triplet excited states.

Compound	S_1 (eV) (nm)	f_{S_1}	S_2 (eV) (nm)	f_{S_2}	T_1 (eV)	T_2 (eV)	T_3 (eV)	$\Delta E_{S_1-T_1}$ (eV)	$\Delta E_{S_1-T_2}$ (eV)[a]	$\Delta E_{T_1-T_2}$ (eV)
**1-TPA-CNBT**	2.47 (501)	0.321	3.71 (334)	0.093	2.11	2.81	3.22	0.36	- 0.34	0.70
**2-TPA-CNBT**	2.38 (522)	0.683	2.71 (458)	0	1.98	2.44	2.91	0.40	- 0.06	0.46
**1-TPA-iCNBT**	2.49 (498)	0.396	3.79 (327)	0.337	2.05	2.63	3.14	0.44	- 0.14	0.58
**2-TPA-iCNBT**	2.38 (521)	0.346	2.61 (474)	0.032	2.12	2.25	2.75	0.26	+ 0.13	0.13

[a] Smallest calculated singlet-triplet energy gap between the first excited singlet state and the second excited triplet state.

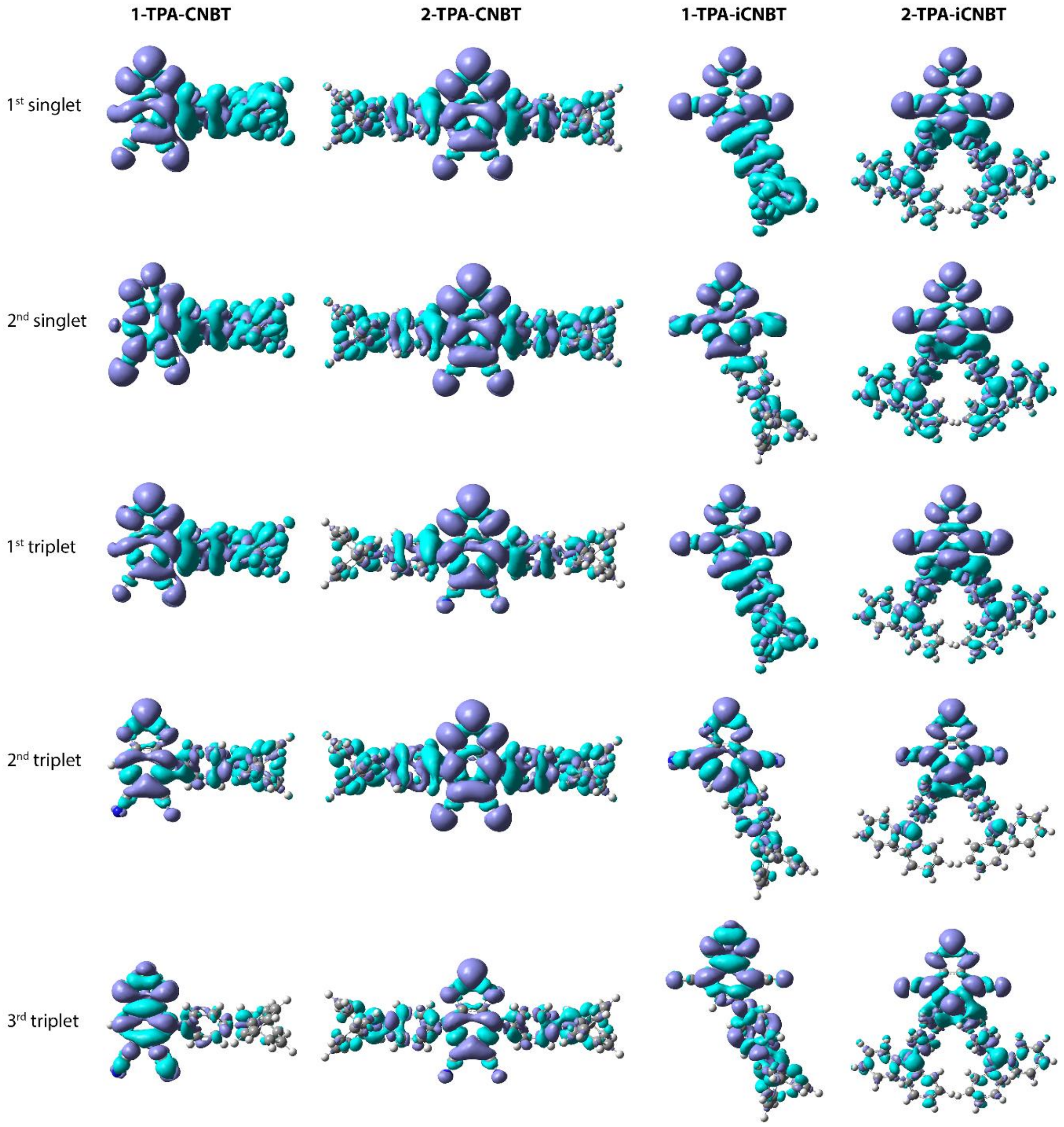

Figure S1: Ground – excited state electron density differences for the singlet and triplet excited states of **1TPA-CNBT**, **2TPA-CNBT**, **1TPA-iCNBT**, and **2TPA-iCNBT**. Purple areas indicate increased electron density, while cyan areas point to decrease electron density (isosurface value = 0.0004 a.u. for all densities).

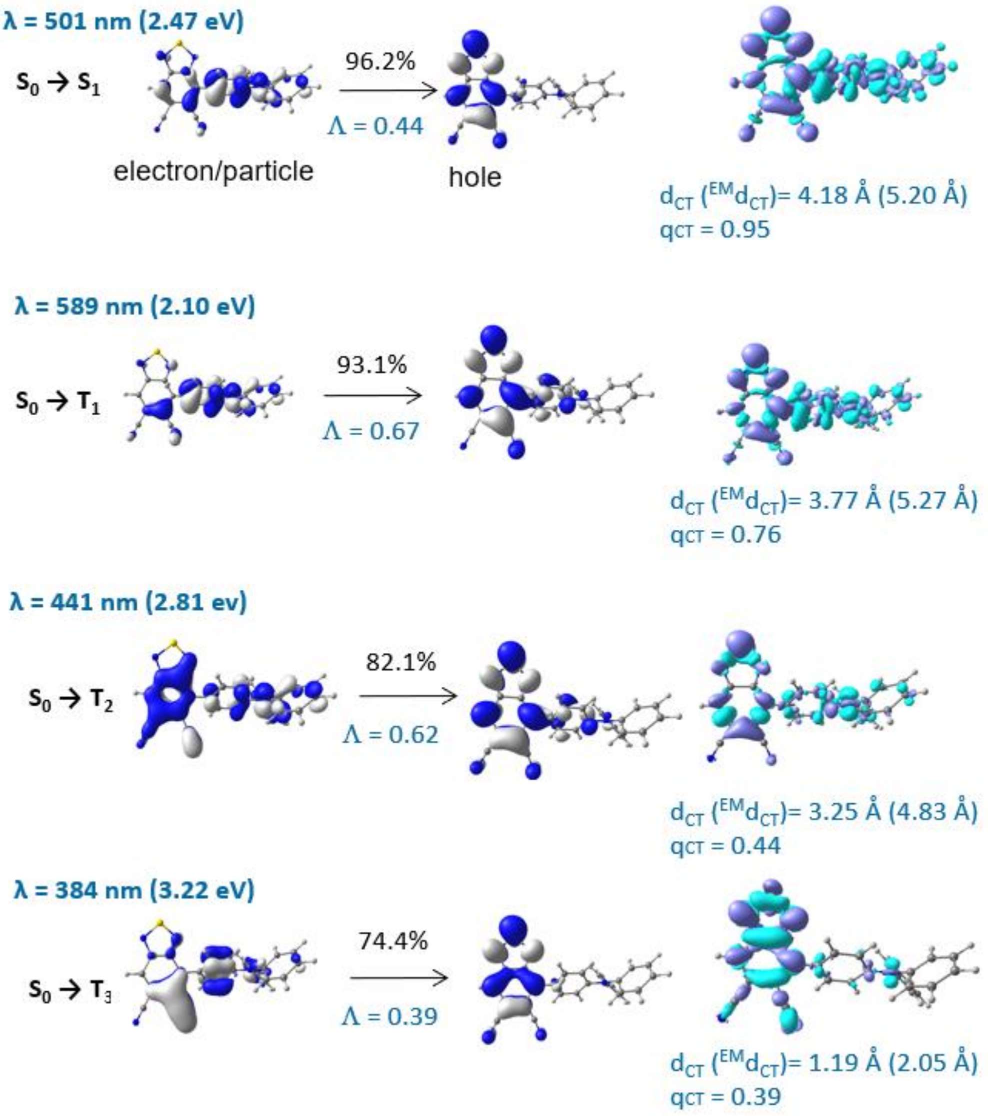

Figure S2: NTO analysis for the key electronic transitions of **1TPA-CNBT** (their excitation energies/wavelengths are reproduced for completeness). Each transition is described by the dominant particle/hole pair, its contribution percentage, as well as the overlap (Λ) between the electron and hole. For comparison, the corresponding differences of electron density upon excitation are provided together with their characteristics (d_{CT}, $^{EM}d_{CT}$, and q_{CT}). All calculations were performed at the IEFPCM [cyclohexane, non-equilibrium]/TDA/LC-BLYP(ω=0.17)/6-311G* level of approximation (isosurface value = 0.03 e/Å^3 for all NTO's while 0.001 e/Å^3 for the densities).

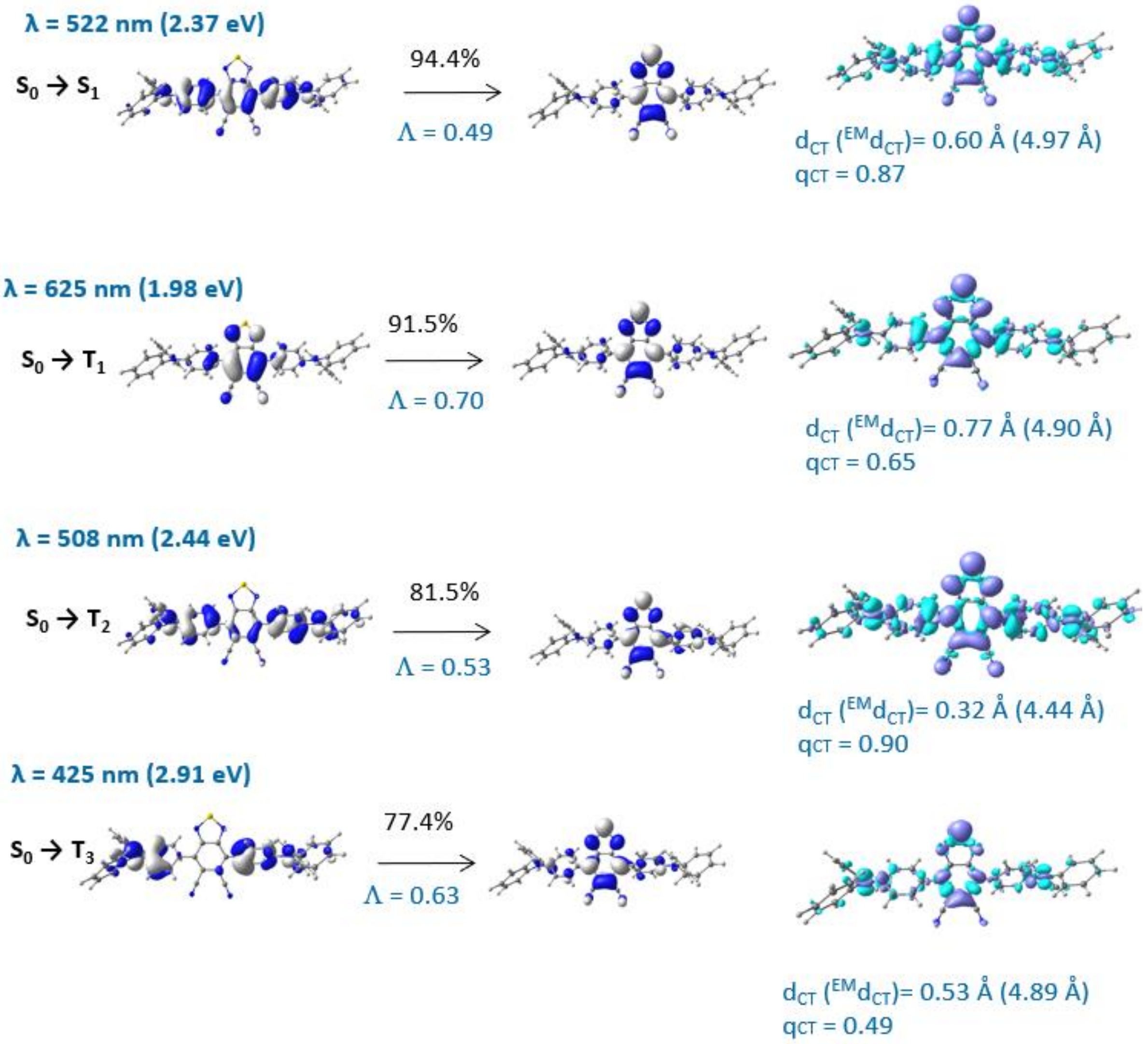

Figure S3: NTO analysis for the key electronic transitions of **2TPA-CNBT** (their excitation energies/wavelengths are reproduced for completeness). Each transition is described by the dominant particle/hole pair, its contribution percentage, as well as the overlap (Λ) between the electron and hole. For comparison, the corresponding differences of electron density upon excitation are provided together with their characteristics (d_{CT}, $^{EM}d_{CT}$, and q_{CT}). All calculations were performed at the IEFPCM [cyclohexane, non-equilibrium]/TDA/LC-BLYP(ω=0.17)/6-311G* level of approximation (isosurface value = 0.03 e/Å^3 for all NTO's while 0.001 e/Å^3 for the densities).

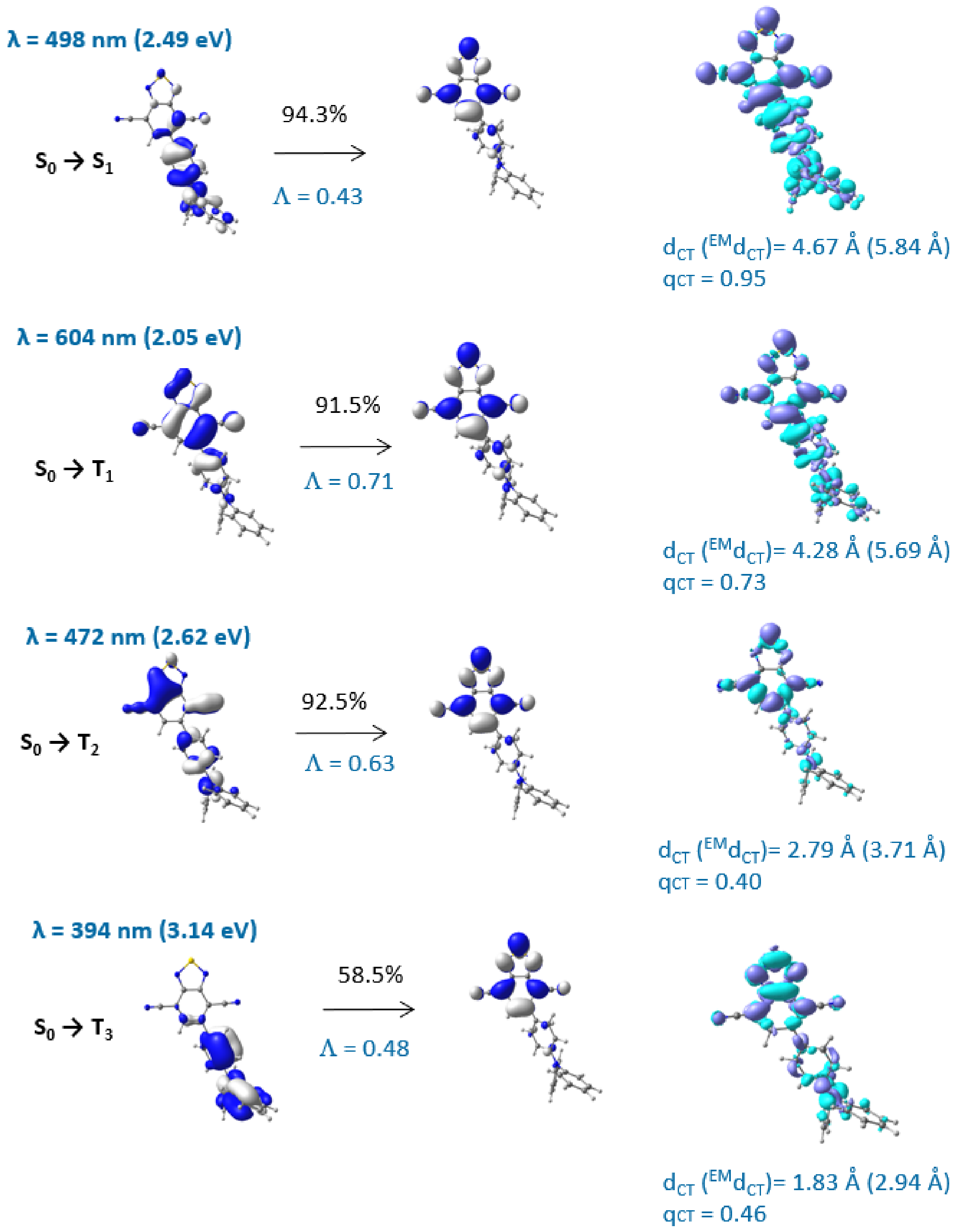

Figure S4: NTO analysis for the key electronic transitions of **1TPA-iCNBT** (their excitation energies/wavelengths are reproduced for completeness). Each transition is described by the dominant particle/hole pair, its contribution percentage, as well as the overlap (Λ) between the electron and hole. For comparison, the corresponding differences of electron density upon excitation are provided together with their characteristics (d_{CT}, ${}^{EM}d_{CT}$, and q_{CT}). All calculations were performed at the IEFPCM [cyclohexane, non-equilibrium]/TDA/LC-BLYP(ω=0.17)/6-311G* level of approximation (isosurface value = 0.03 e/Å^3 for all NTO's while 0.001 e/Å^3 for the densities).

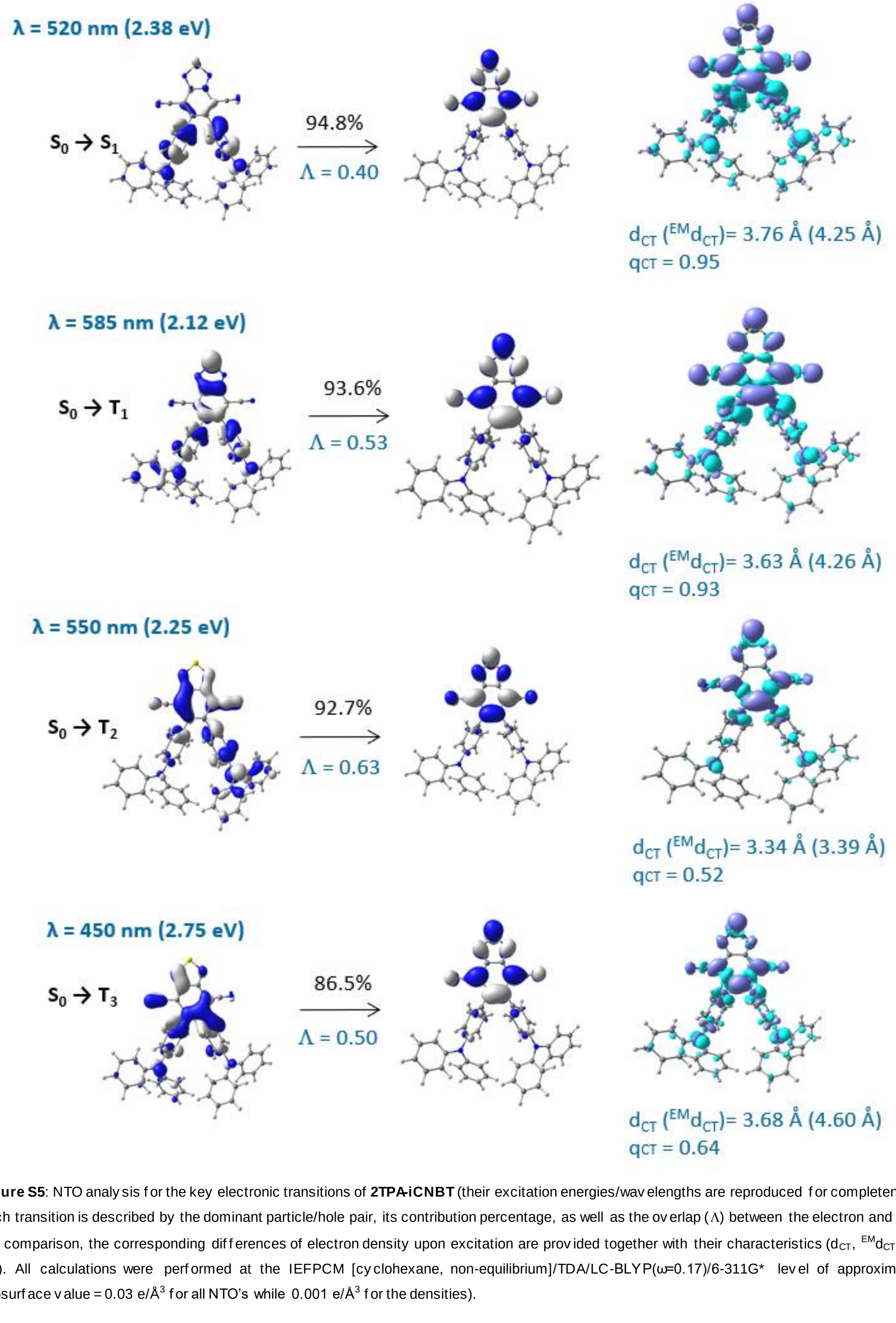

Figure S5: NTO analysis for the key electronic transitions of **2TPA-iCNBT** (their excitation energies/wavelengths are reproduced for completeness). Each transition is described by the dominant particle/hole pair, its contribution percentage, as well as the overlap (Λ) between the electron and hole. For comparison, the corresponding differences of electron density upon excitation are provided together with their characteristics (d_{CT}, ${}^{EM}d_{CT}$, and q_{CT}). All calculations were performed at the IEFPCM [cyclohexane, non-equilibrium]/TDA/LC-BLYP(ω=0.17)/6-311G* level of approximation (isosurface value = 0.03 e/Å^3 for all NTO's while 0.001 e/Å^3 for the densities).

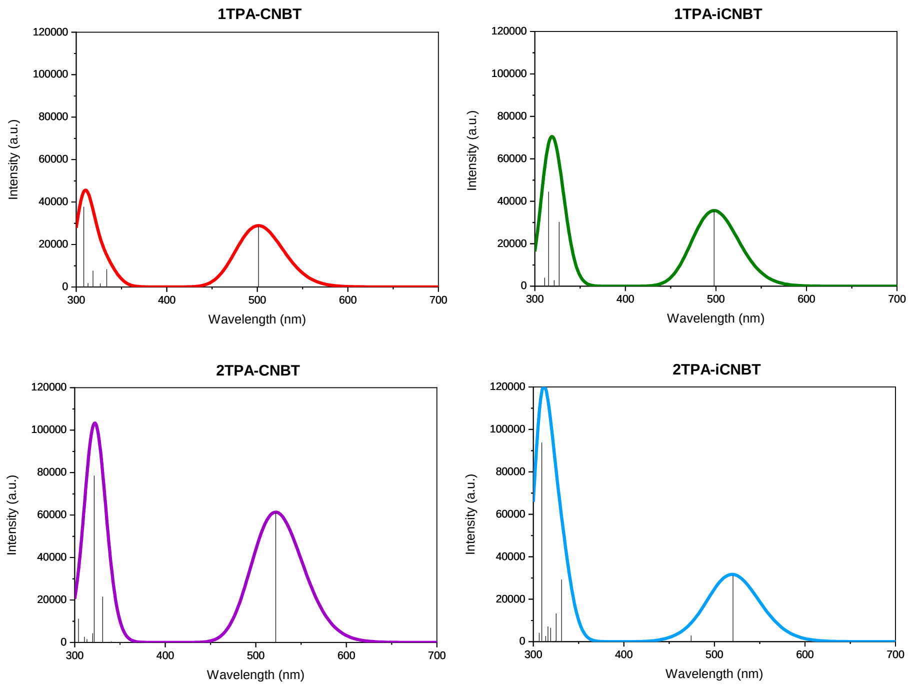

Figure S6: Simulated UV-Vis absorption spectra derived from the TDDFT results for the optimized geometries by fitting the vertical excitation energies with a Gaussian fit and employing a full-width-at-half-maximum of 0.3 eV for each excitation. The vertical lines indicate the specific excitation energies and their relative size is indicative of the oscillator strength accompanying the transition.

4. Absolute absorption spectra and singlet oxygen quantum yields in toluene

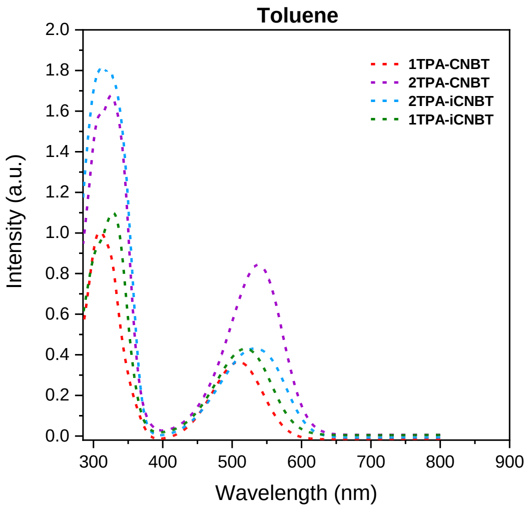

Figure S7: Steady-state absorption spectra for all four emitters in 10^{-5} M toluene solution.

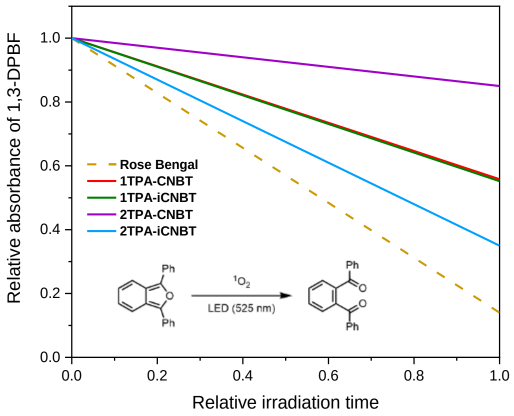

Figure S8: Relative decrease in absorbance of 1,3-diphenylisobenzofuran at 414 nm under continuous irradiation using a single 525 nm LED in the presence of the respective emitter in toluene. Rose Bengal was used as a standard (Φ_Δ = 0.86 in ethanol).

5. Solution state absorption and emission spectra for solvatochromism determination

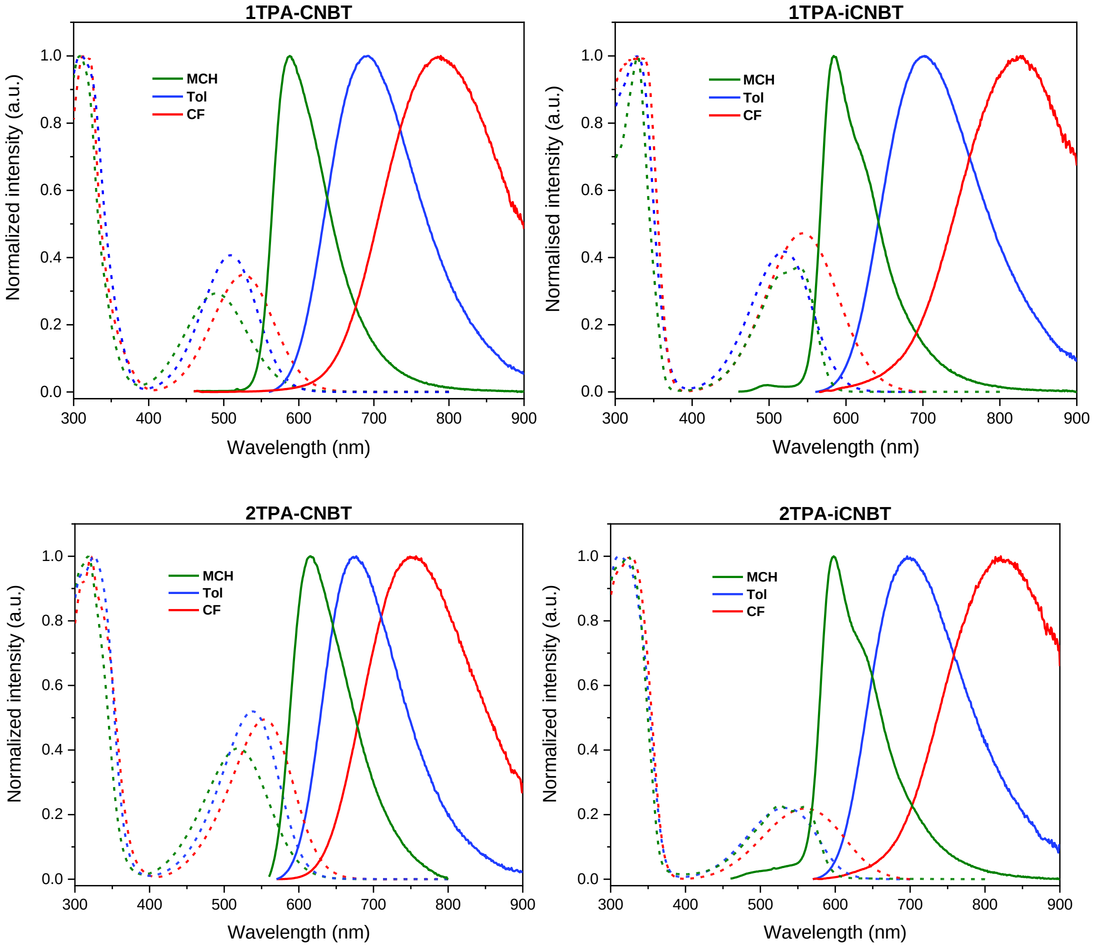

Figure S9: Normalized steady-state absorption spectra (dashed lines) and emission spectra (solid lines) for the four emitters in methylcyclohexane (MCH, blue), toluene (TOL, green), and chloroform (CF, red).

Table S3. Absorption and emission peak maxima for the four emitters in solutions of varying polarity.

Compound	$\lambda_{CT\ abs}$ (nm)[a]			λ_{em} (nm)[b]		
	MCH	TOL	CF	MCH	TOL	CF
**1TPA-CNBT**	490	509	526	587	690	786
**1TPA-iCNBT**	511, 540	518	544	585	700	824
**2TPA-CNBT**	519	538	554	615	674	752
**2TPA-iCNBT**	525, 557	533	559	598	698	822

[a] Absorption maxima for the CT band in methylcyclohexane (MCH), toluene (TOL), and chloroform (CF) solution. [b] Fluorescence emission maxima in MCH, TOL, and CF solution (10^{-5} M).

6. Cyclic voltammetry and HOMO/LUMO determination

Cyclic voltammetry experiments were performed to estimate the HOMO and LUMO energies of the four materials.

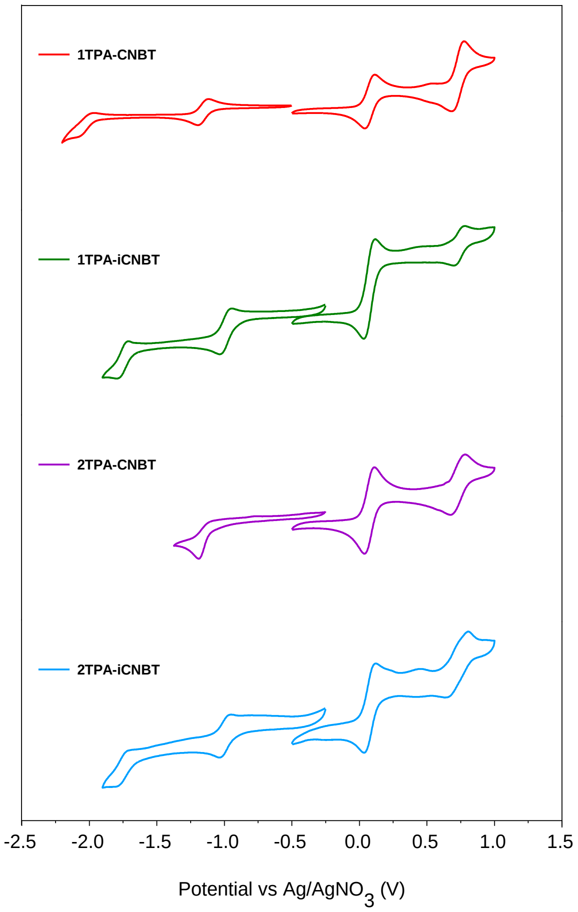

Figure S10: Cyclic voltammograms for the four materials determined in acetonitrile at room temperature with $TBAPF_6$ as the supporting electrolyte. Combined oxidation and reduction voltammograms are shown.

7. Energy gap determination from steady-state fluorescence and phosphorescence spectra

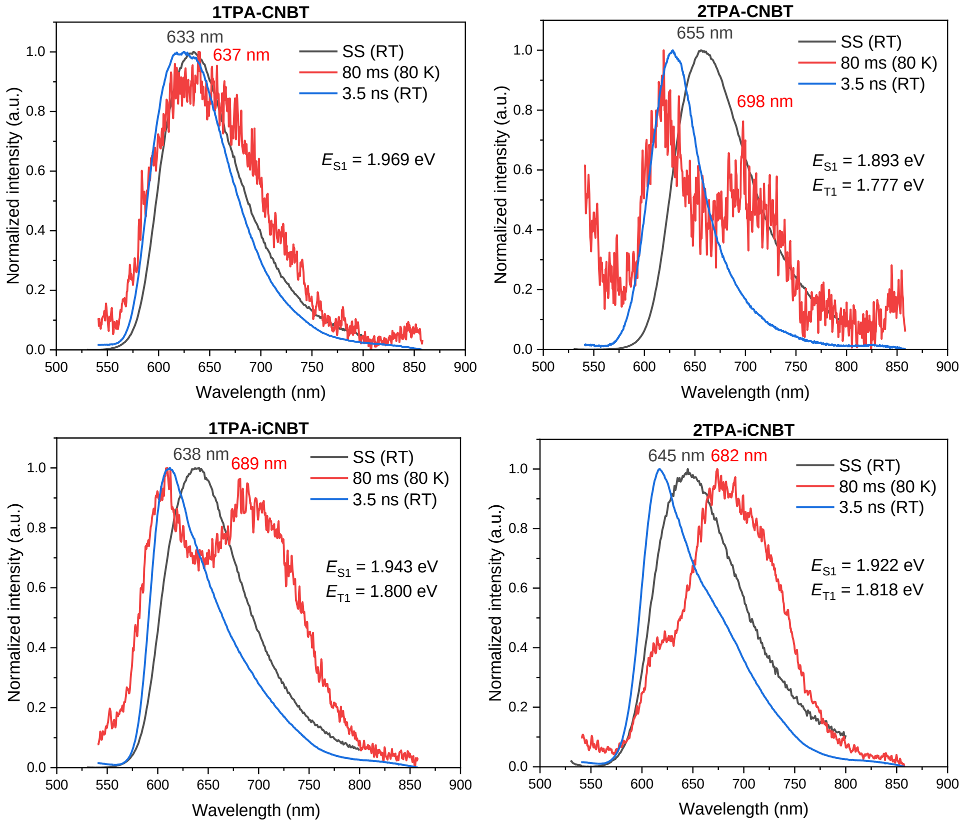

Figure S11: Experimental singlet-triplet energy gap determination via the comparison of steady-state fluorescence spectra at room temperature and 80-milliseconds delay-time spectra at 80 K (nominally phosphorescence) for the four emitters in 1% zeonex films. The blue-shifted 80-millisecond delay-time spectrum of **1TPA-CNBT** may arise from triplet-triplet annihilation, and can therefore not be unequivocally attributed to phosphorescence. The 80-milliseconds delay-time spectra of **2TPA-CNBT**, **1TPA-iCNBT,** and **2TPA-iCNBT** contain multiple overlapping emission bands, imposing a severe numerical uncertainty on the phosphorescence band onset. Therefore, ΔE_{ST} was estimated from the emission peak maxima instead of the onset.

8. Normalized time-resolved emission spectra

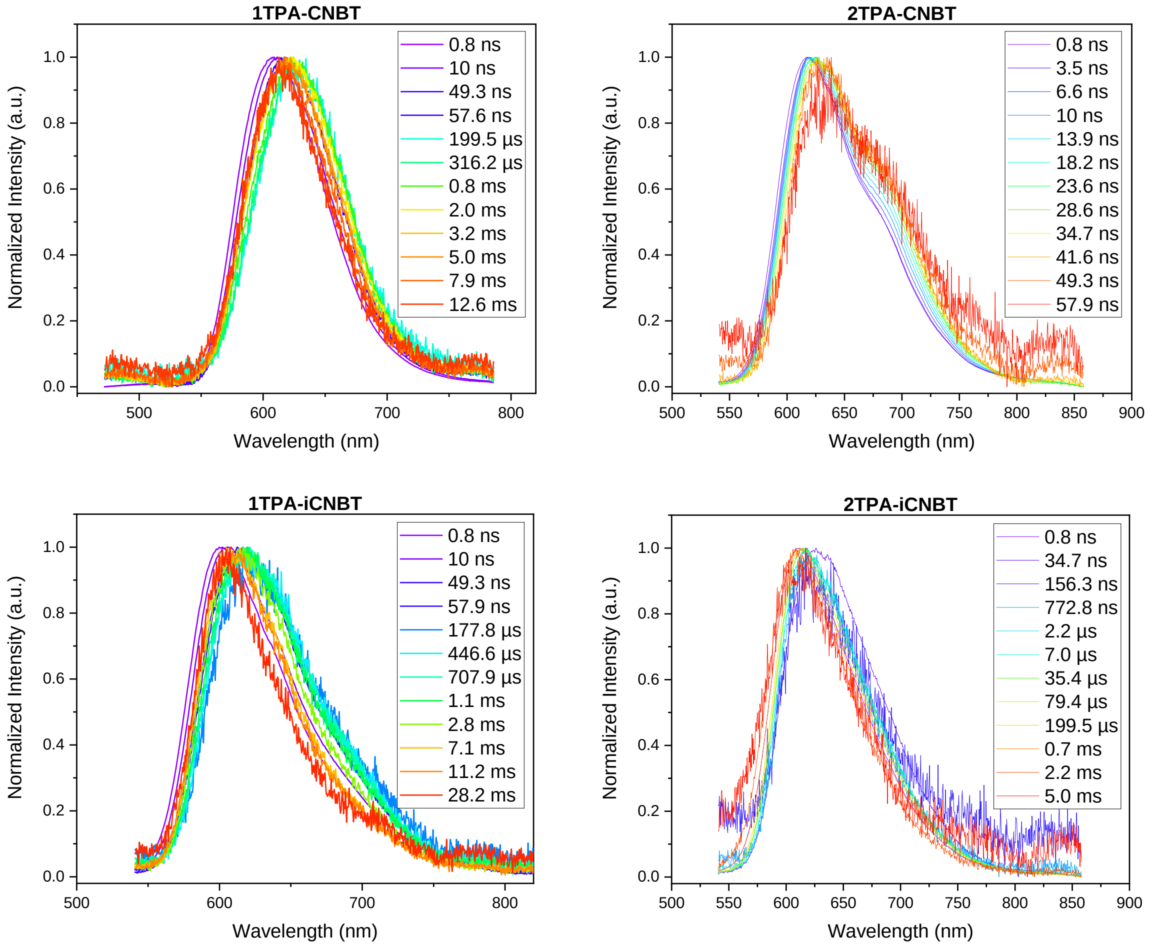

Figure S12: Snapshots of the emission at various time scales for the four emitters in 1% zeonex films at room temperature.

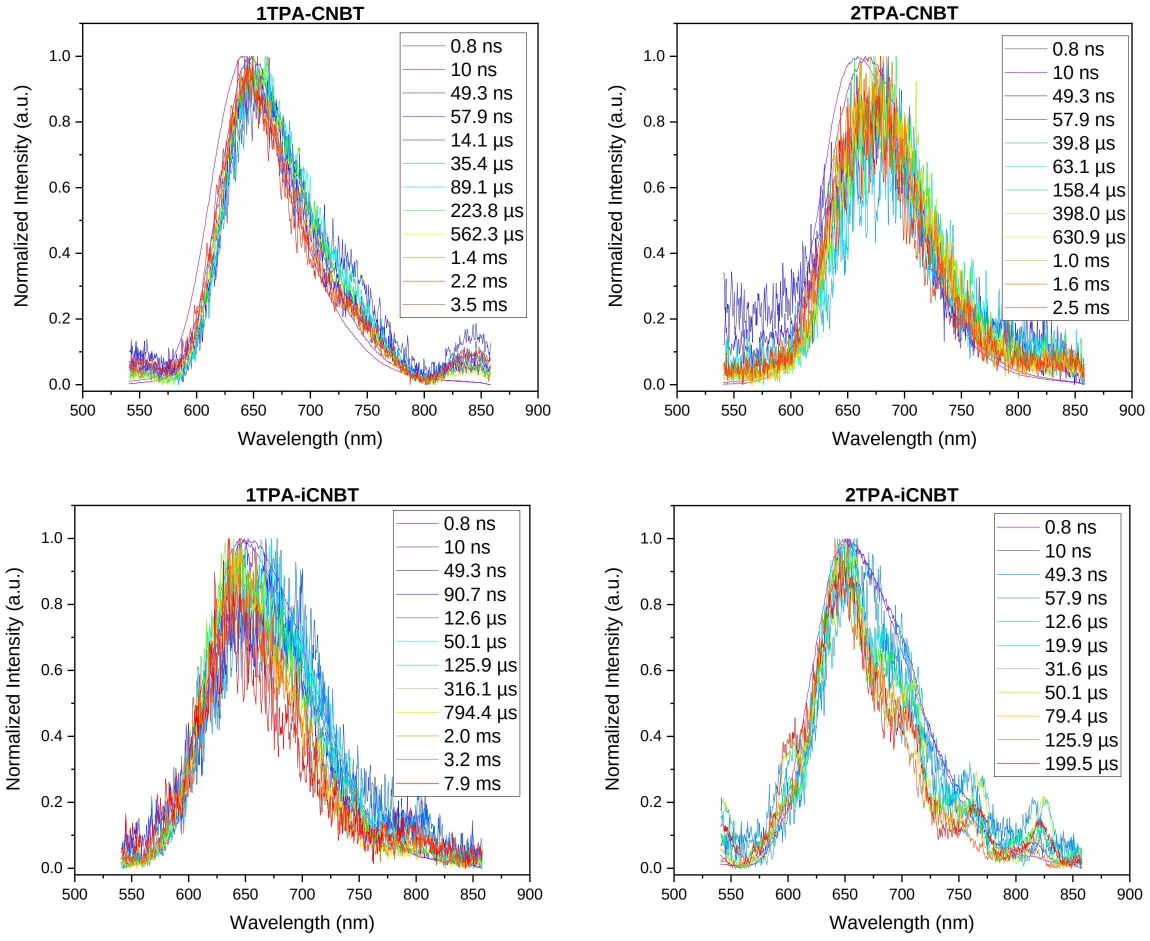

Figure S13: Snapshots of the emission at various time scales for the four emitters in 10% CBP films at room temperature.

9. Normalized time-resolved emission contour plots and kinetic fitting of the emission decay

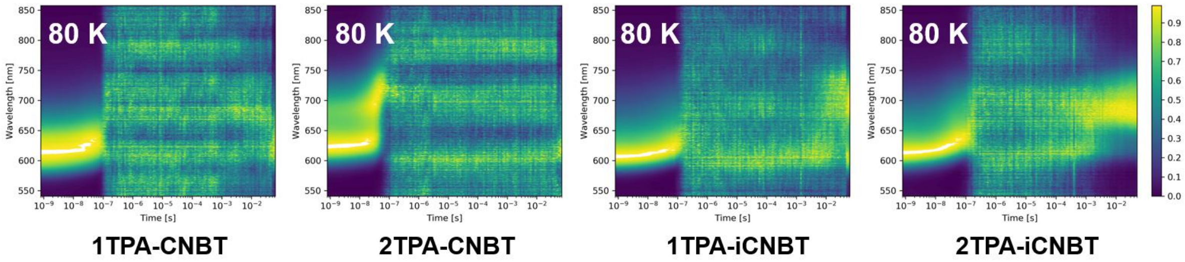

Figure S14: Normalized time-resolved emission spectra (contour plots) for all 4 materials in 1 w/w% zeonex films at 80 K.

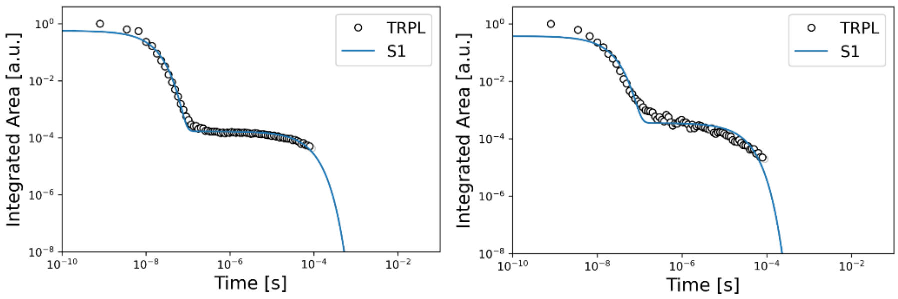

Figure S15: Kinetic fitting [7] of the emission decay curves of **2TPA-iCNBT** in 1 w/w% Zeonex (left) and 10 w/w% CBP films (right).

Table S4. Prompt and delayed emission lifetimes.

Compound	τ_p (ns)		τ_d (µs)	
	zeonex	CBP	zeonex	CBP
**1TPA-CNBT**	6.48/12.4	9.40/16.7	/[a]	58.6
**1TPA-iCNBT**	10.2/15.9	4.26/25.3	/[a]	58.8
**2TPA-CNBT**	4.08/10.6	7.13/11.8	/[a]	52.5
**2TPA-iCNBT**	12.0	7.99/23.0	62.4	40.9

[a] Delayed lifetimes could not be accurately determined due to poor fitting of the emission decay as a result of weak emission contribution.

10. Device optimization

10 w/w% films were prepared in OLED-relevant hosts such as CBP, guided by its triplet energy alignment and common usage in the characterization of NIR (TADF) materials. The 10 w/w% concentration was chosen based on prior experience as a reliable starting point for device fabrication and was also necessary to obtain sufficient emission intensity for the CCD-based time-resolved measurements. Additionally, devices with 15% and 20% doping were fabricated and tested (*vide infra*) but no consistent performance improvement was observed, supporting the decision to use 10 w/w% uniformly across the series.

The initial OLED architecture, which incorporates DPEPO and TPBi as the hole-blocking and electron-transport layers, respectively, follows a standard device stack which is routinely employed during OLED fabrication. An alternative electron transport material, TmPyPB, was subsequently introduced based on internal studies where it consistently improved device efficiency in related systems.

Table S5. Device parameters for OLEDs based on **2TPA-iCNBT**, **1TPA-iCNBT**, **2TPA-CNBT**, and **1TPA-CNBT.**

Emitter	Host (w/w% dopant)	Solvent	Pixel	V_{on} (V)[a]	$\lambda_{EL,max}$ (nm)[b]	EQE_{max} (%)[c]	L_{max} ($cd.m^{-2}$)[d]
Stack: ITO | PEDOT:PSS | EML | 'DPEPO' (10 nm) | TPBi (40 nm) | LiF (1 nm) | Al (100 nm)							
**2TPA-iCNBT**	CBP (10)	toluene	2x4	5.5	688	**0.54 (6.5 V)**	109 (10.5 V)
	CBP (10)	toluene	2x2	5.5	688	0.42 (6.5 V)	121 (10.5 V)
	CBP (10)	toluene	2x4	4	683*[e]	0.30 (4.5 V)	233 (8.5 V)
	CBP (15)	toluene	4x4	4.5	691	0.29 (5.5 V)	46 (12 V)
	CBP (20)	toluene	2x4	4	699	0.13 (5 V)	119 (10 V)
	CBP (10)	CF	4x4	5	678	0.22 (7.5 V)	82 (11.5 V)
	CBP (15)	CF	4x2	5.5	684	0.3 (7.5 V)	133 (13 V)
	CBP (20)	CF	4x4	4.5	695	0.092 (6.5 V)	51 (10 V)
	Neat[f]	toluene	4x4	6.5	770	0.0056 (9.5 V)	3.6 (13 V)
	Neat[f]	toluene	4x2	4	775*[e]	0.0114 (6 V)	8 (9 V)
	Neat[f]	CF	4x2	6.5	762	0.0042 (10 V)	3.8 (9.5 V)
**2TPA-CNBT**	CBP (10)	toluene	4x2	4.5	674	**0.73 (4.5 V)**	180 (10 V)
	CBP (10)	toluene	2x4	4.5	674	0.63 (4.5 V)	183 (10.5 V)
**1TPA-CNBT**	CBP (10)	toluene	2x4	4.5	658	0.45 (4.5 V)	23 (11 V)
	CBP (10)	toluene	2x4	4.5-5	658	**0.5 (5 V)**	128 (9.5 V)
**1TPA-iCNBT**	CBP (10)	toluene	2x4	5	688	0.34 (5 V)	157 (10 V)
	CBP (10)	toluene	4x2	4.5	688	**0.68 (4.5 V)**	349 (10.5 V)
Stack: ITO | PEDOT:PSS | EML | TmPyPB (60 nm) | LiF (1 nm) | Al (100 nm)							
**2TPA-iCNBT**	CBP (10)	toluene	4x2	4.5	684	2.21 (5 V)	208 (9.5 V)
	CBP (10)	toluene	2x2	4.5	684	**2.49 (5 V)**	216 (9 V)
**2TPA-CNBT**	CBP (10)	toluene	4x4	4.5	671	**1.16 (4.5 V)**	361 (9 V)
	CBP (10)	toluene	4x4	4.5	671	1.05 (5.5 V)	364 (9 V)
**1TPA-CNBT**	CBP (10)	toluene	2x4	4.5	664	2.56 (4.5 V)	657 (9.5 V)
	CBP (10)	toluene	2x4	4.5-5	664	**2.91 (5 V)**	531 (10.5 V)
**1TPA-iCNBT**	CBP (10)	toluene	2x4	4.5	679	**2.76 (4.5 V)**	265 (9.5 V)
	CBP (10)	toluene	4x2	4.5	679	2.15 (5 V)	255 (10.5 V)

[a] Turn-on voltage. [b] Maximum electroluminescence peak. [c] Maximum external quantum efficiency at a specified voltage (given in parentheses). The highest EQE_{max} value for each material within each stack is shown in bold. [d] Maximum luminance obtained at a specified voltage (given in parentheses). [e] No DPEPO used. 2nd emission peak observed around 370 nm (attributed to TPBi emission, **Figure S12**). [f] Deposited from 5 mg/mL toluene solution.

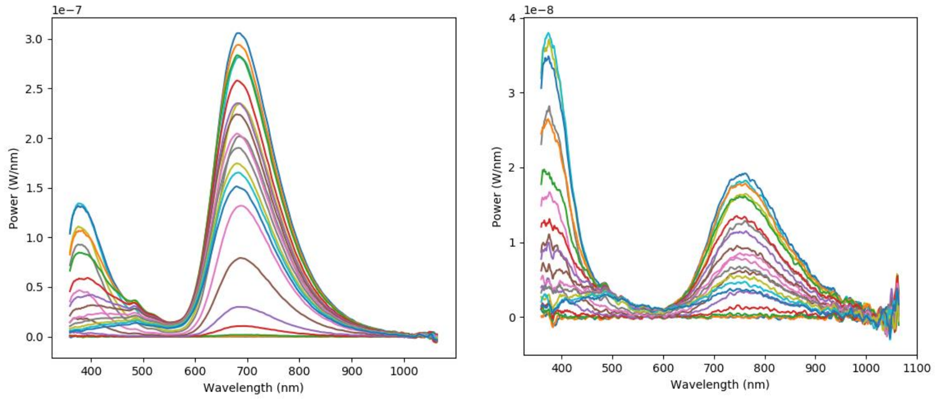

Figure S16: EL spectra of the DPEPO-free, 10 w/w% doped (left), and neat (right) **2TPA-iCNBT** OLED stacks. The emission peak below 400 nm is attributed to TPBi.

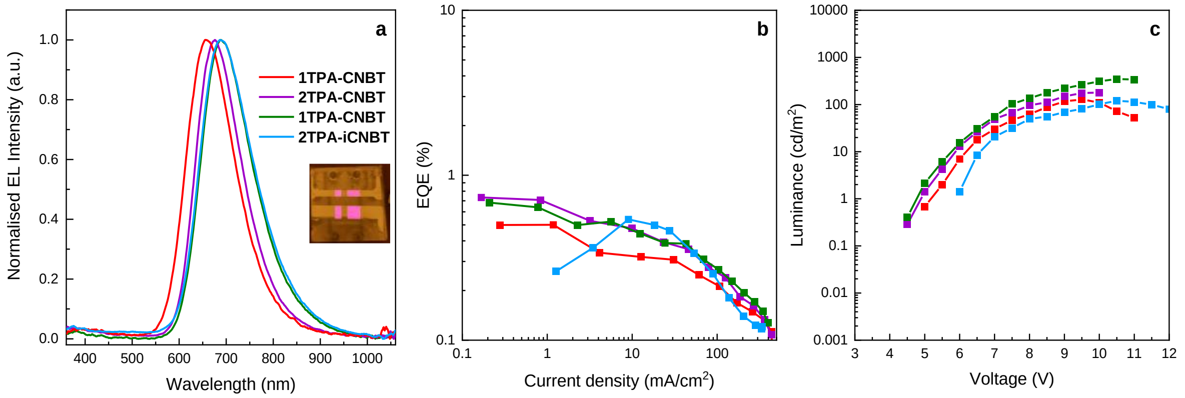

Figure S17: (a) EL spectra (with photograph of the **2TPA-iCNBT** OLED device), (b) EQE-current density curves, and (c) luminance versus voltage plots of the four doped (10 w/w% in CBP) OLED devices based on the original DPEPO/TPBi stack.

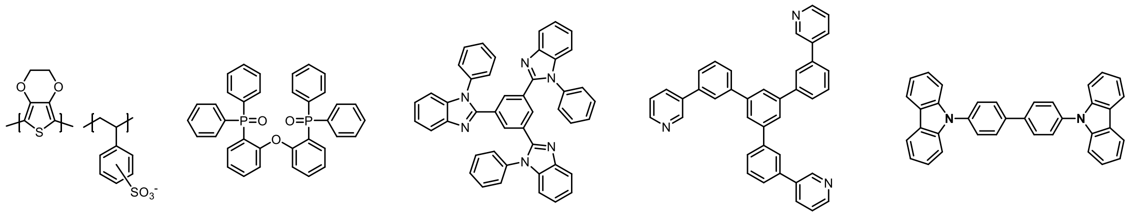

PEDOT:PSS **DPEPO** **TPBI** **TmPyPB** **CBP**

Figure S18: Molecular structures of additional materials used in the device stacks.

11. Comparative electroluminescence performance

Table S6. EL performance of representative red to near-infrared TADF OLEDs fabricated by solution processing (spin coating)

Emitter (w/w% in host)	Host	EL_{max} (nm)[a]	EQE (%)[b]	V_{on} (V)[c]	OLED stack (layer thickness)	Ref.
CNPP-TPA (50)	CBP	700	2.02	5.8	ITO/PEDOT:PSS (20 nm)/CBP:**CNPP-TPA** (100 nm)/DPEPO (5 nm)/TPBi (40 nm)/LiF/Al	[8]
TPA-BF2 (6)	F8BT	715	2.2	8.4	ITO (100 nm)/PEDOT:PSS (45nm)/F8BT:**TPA-BF2** (80 nm)/DPEPO (10 nm)/TPBi (55 nm)/LiF (1nm)/Al (100nm)	[9]
TPA-BF2 (6)	CBP	721	9.69	/	ITO/PEDOT:PSS (45 nm)/CBP:**TPA-BF2** (80 nm)/DPEPO (10 nm)/TPBi (55 nm)/LiF (1 nm)/Al (100 nm)	[9]
4DMAC-TPPQ (10)	CBP	640	3.9	4.2	ITO/PEDOT:PSS (40 nm)/F8-TFB (25 nm)/CBP:**4DMAC-TPPQ** (25 nm)/ TmPyPB (70 nm)/LiF (1 nm)/Al (100 nm)	[10]
4DMAC-TPPQ (50)	CBP	670	0.8	4.4	ITO/PEDOT:PSS (40 nm)/F8-TFB (25 nm)/CBP:**4DMAC-TPPQ** (25 nm)/ TmPyPB (70 nm)/LiF (1 nm)/Al (100 nm)	[10]
4PXZ-TPPQ (10)	CBP	686	1.7	5.2	ITO/PEDOT:PSS (40 nm)/F8-TFB (25 nm)/CBP:**4PXZ-TPPQ** (25 nm)/ TmPyPB (70 nm)/LiF (1 nm)/Al (100 nm)	[10]
4PXZ-TPPQ (50)	CBP	735	0.3	4.4	ITO/PEDOT:PSS (40 nm)/F8-TFB (25 nm)/CBP:**4PXZ-TPPQ** (25 nm)/ TmPyPB (70 nm)/LiF (1 nm)/Al (100 nm)	[10]
2PN (10)	CBP	687	0.05	>6	ITO/PEDOT:PSS (40 nm)/CBP:**2PN** (20 nm)/TPBi (60 nm)/LiF/Al	[11]
1TPA-CNBT (10%)	CBP	664	2.91	4.5	ITO/PEDOT:PSS/CBP:**1TPA-CNBT**/TmPyPB (60 nm)/LiF (1 nm)/Al (100 nm)	This work
2TPA-CNBT (10%)	CBP	671	1.16	4.5	ITO/PEDOT:PSS/CBP:**2TPA-CNBT**/TmPyPB (60 nm)/LiF (1 nm)/Al (100 nm)	This work
1TPA-iCNBT (10%)	CBP	679	2.76	4.5	ITO/PEDOT:PSS/CBP:**1TPA-iCNBT**/TmPyPB (60 nm)/LiF (1 nm)/Al (100 nm)	This work
2TPA-iCNBT (10%)	CBP	684	2.49	4.5	ITO/PEDOT:PSS/CBP:**2TPA-iCNBT**/TmPyPB (60 nm)/LiF (1 nm)/Al (100 nm)	This work

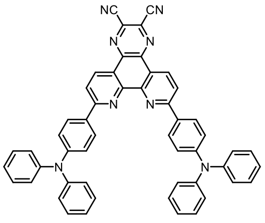

CNPP-TPA

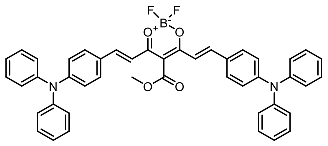

TPA-BF2

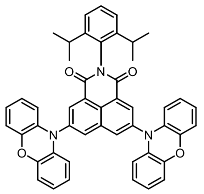

2PN

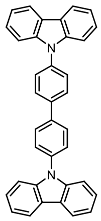

CBP

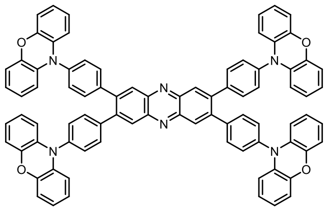

4PXZ-TPPQ

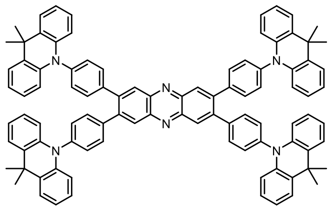

4DMAC-TPPQ

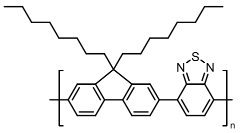

F8BT

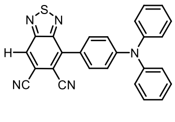

1TPA-CNBT

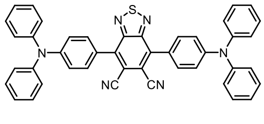

2TPA-CNBT

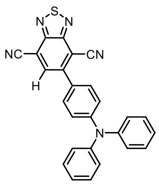

1TPA-iCNBT

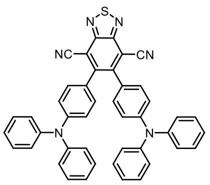

2TPA-iCNBT

[a] Maximum electroluminescence peak (nm). [b] External quantum efficiency. [c] Turn-on voltage.

12. NMR & MALDI-ToF mass spectra

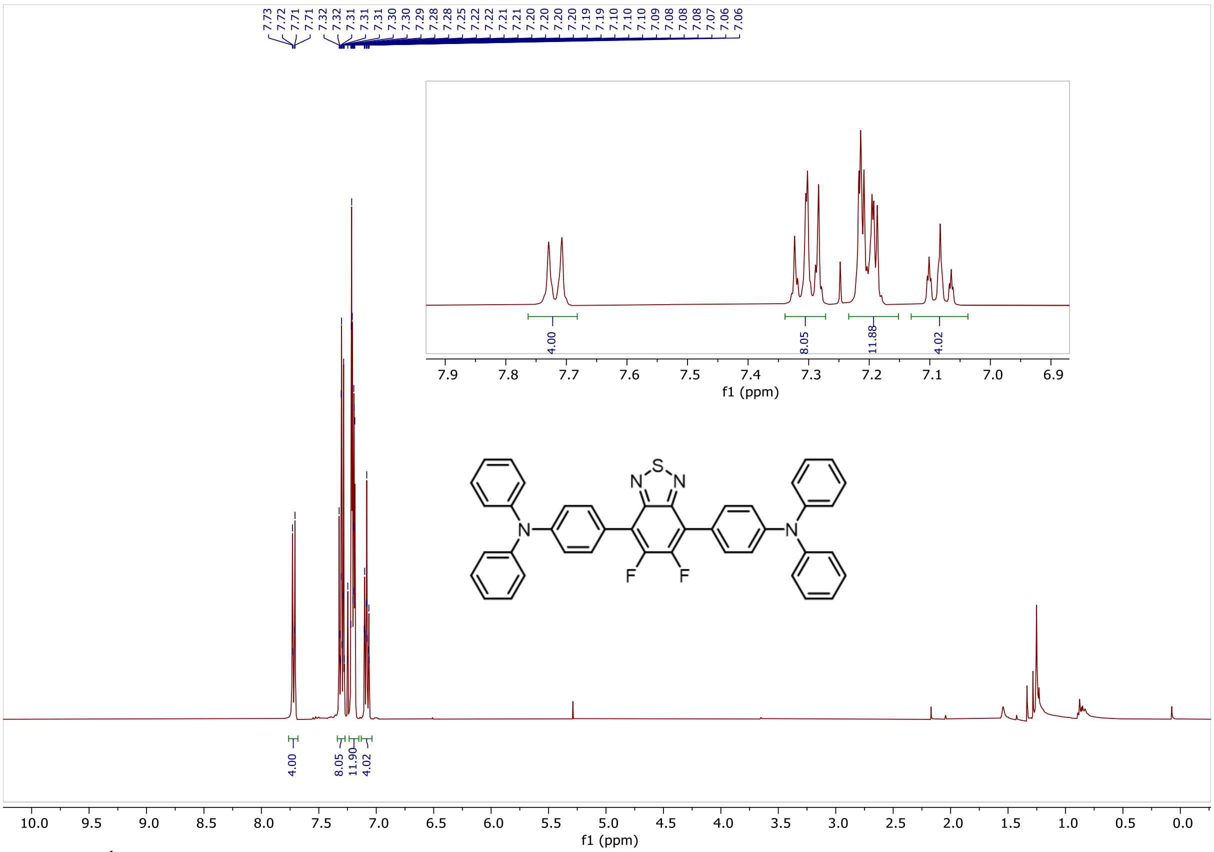

Figure S19: ^{1}H NMR spectrum of 4,4'-(5,6-difluorobenzo[*c*][1,2,5]thiadiazole-4,7-diyl)bis(*N*,*N*-diphenylaniline) (**2**) in $CDCl_3$.

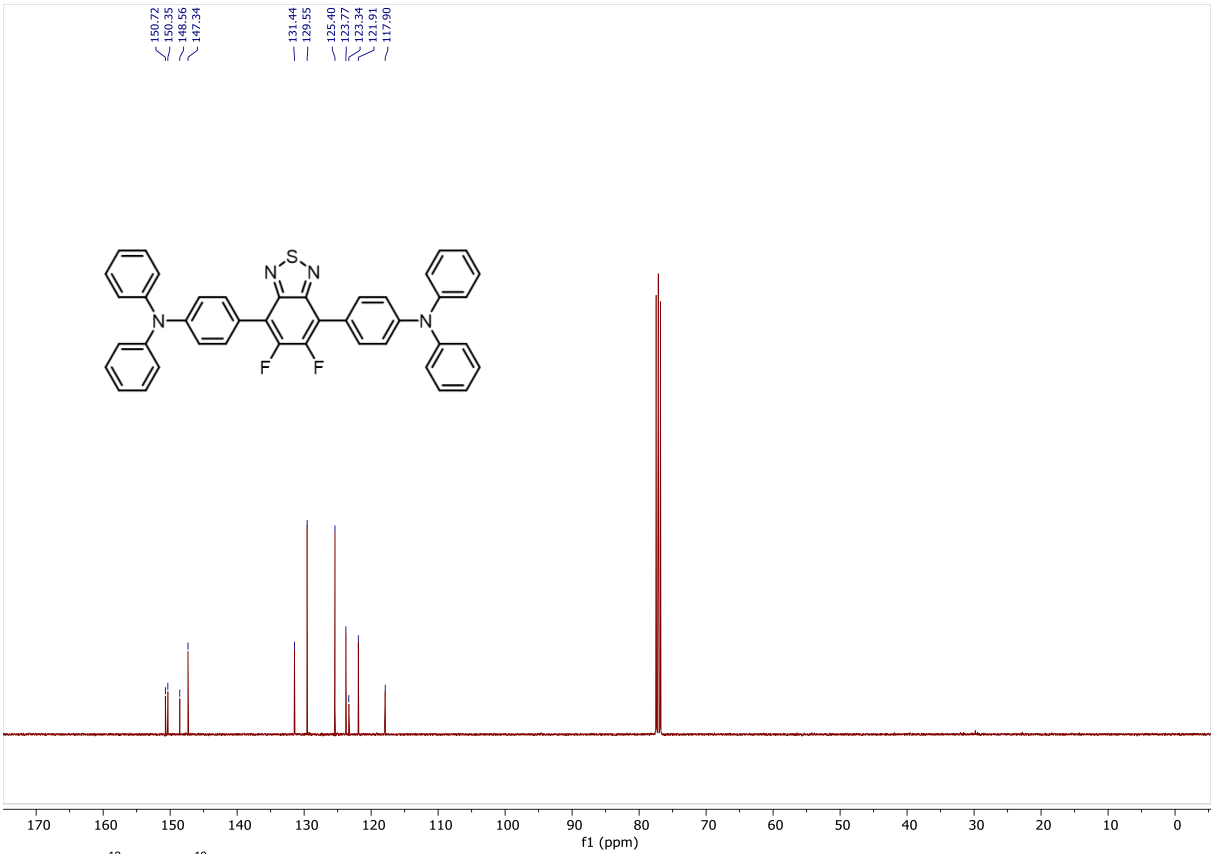

Figure S20: ^{13}C NMR (^{19}F-decoupled) spectrum of 4,4'-(5,6-difluorobenzo[*c*][1,2,5]thiadiazole-4,7-diyl)bis(*N*,*N*-diphenylaniline) (**2**) in $CDCl_3$.

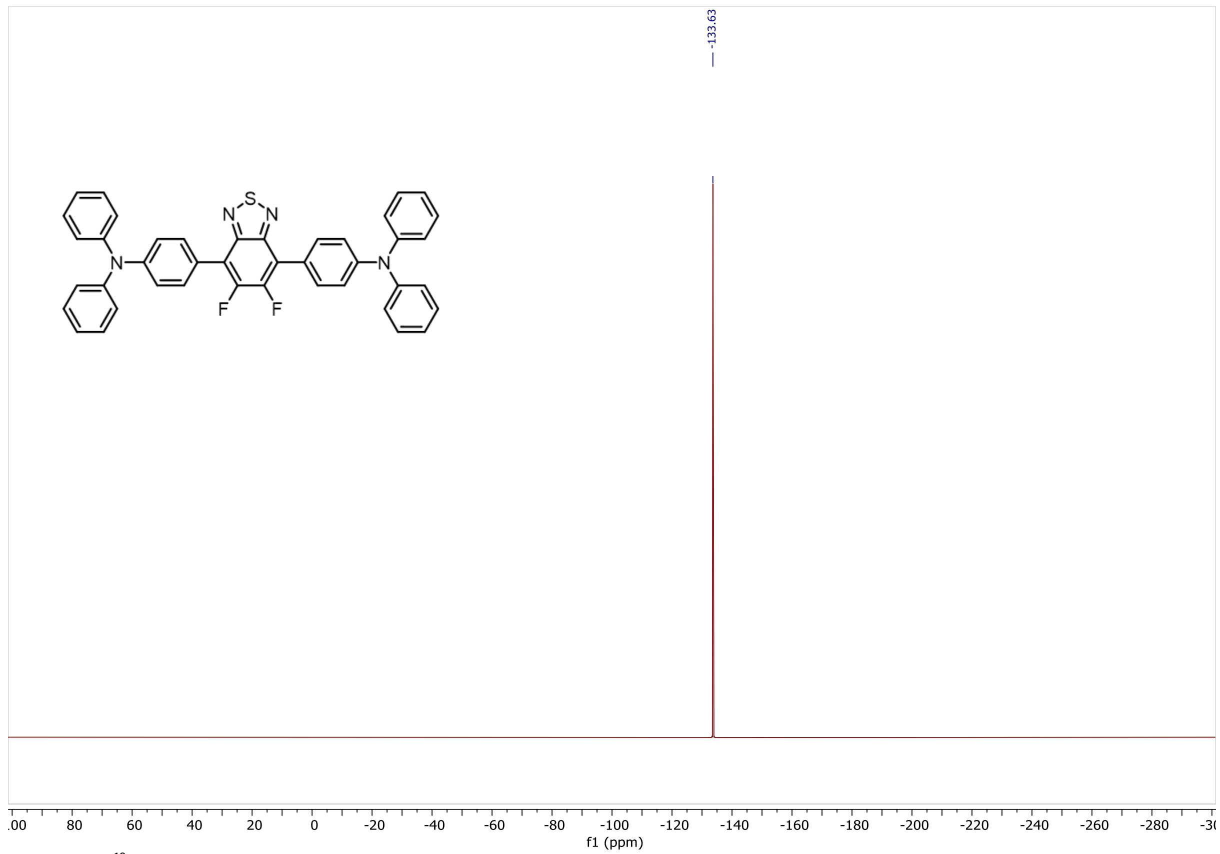

Figure S21: ^{19}F NMR spectrum of 4,4'-(5,6-difluorobenzo[*c*][1,2,5]thiadiazole-4,7-diyl)bis(*N*,*N*-diphenylaniline) (**2**) in $CDCl_3$.

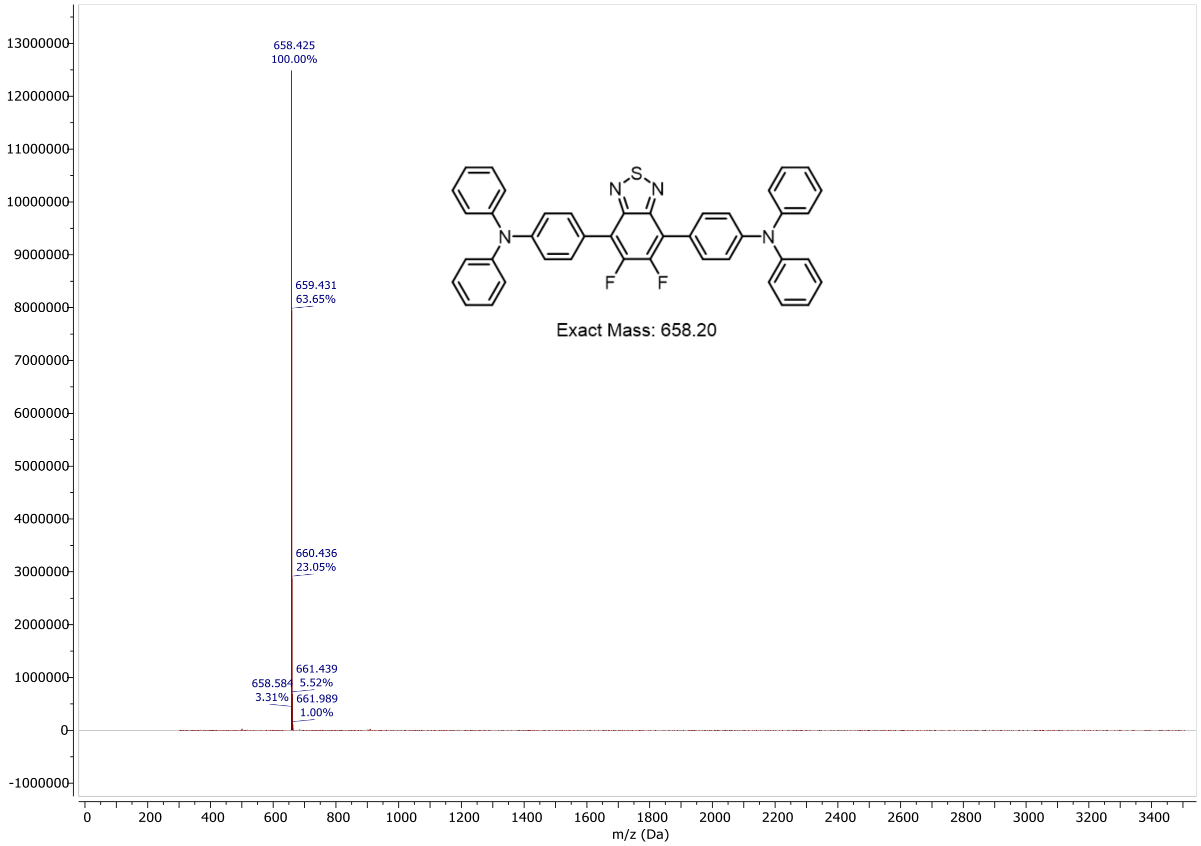

Figure S22: MALDI-ToF mass spectrum of 4,4'-(5,6-difluorobenzo[*c*][1,2,5]thiadiazole-4,7-diyl)bis(*N*,*N*-diphenylaniline) (**2**).

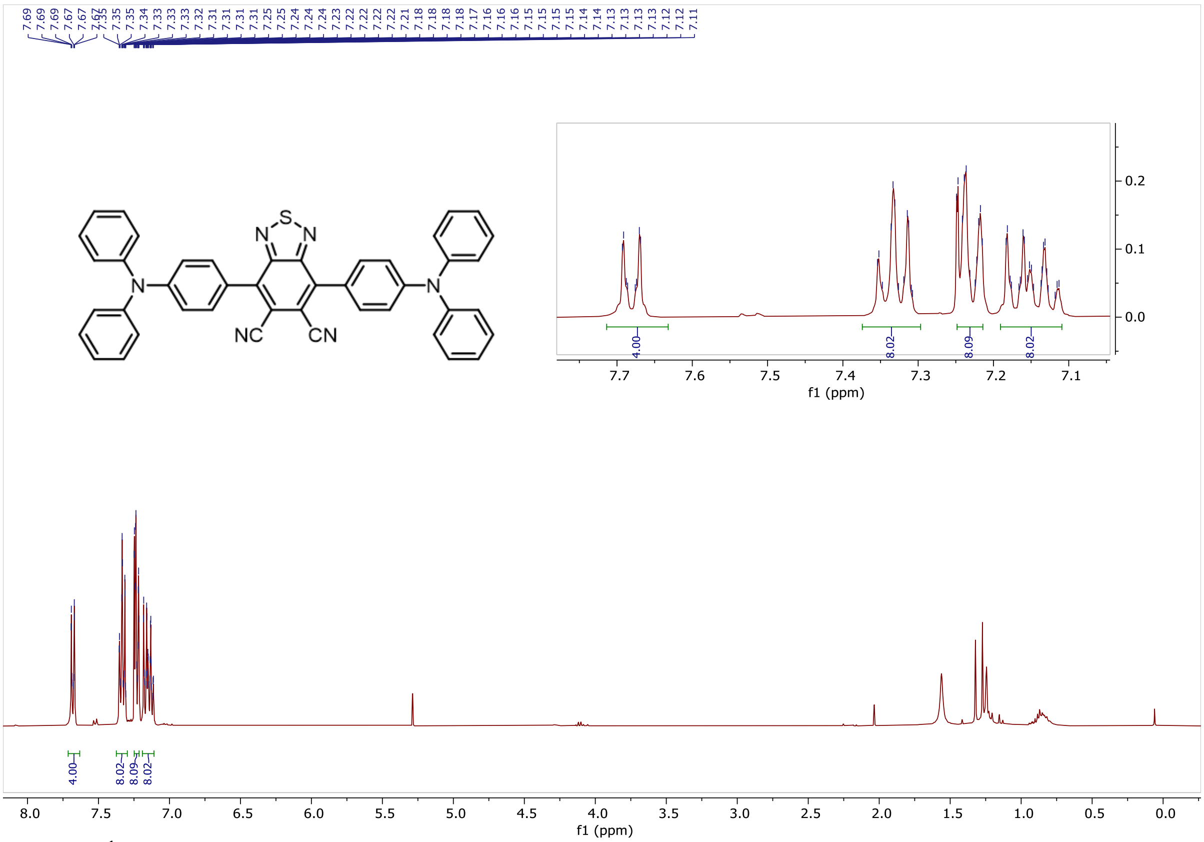

Figure S23: ¹H NMR spectrum of 4,7-bis(4-(diphenylamino)phenyl)benzo[*c*][1,2,5]thiadiazole-5,6-dicarbonitrile (**3**) in $CDCl_3$.

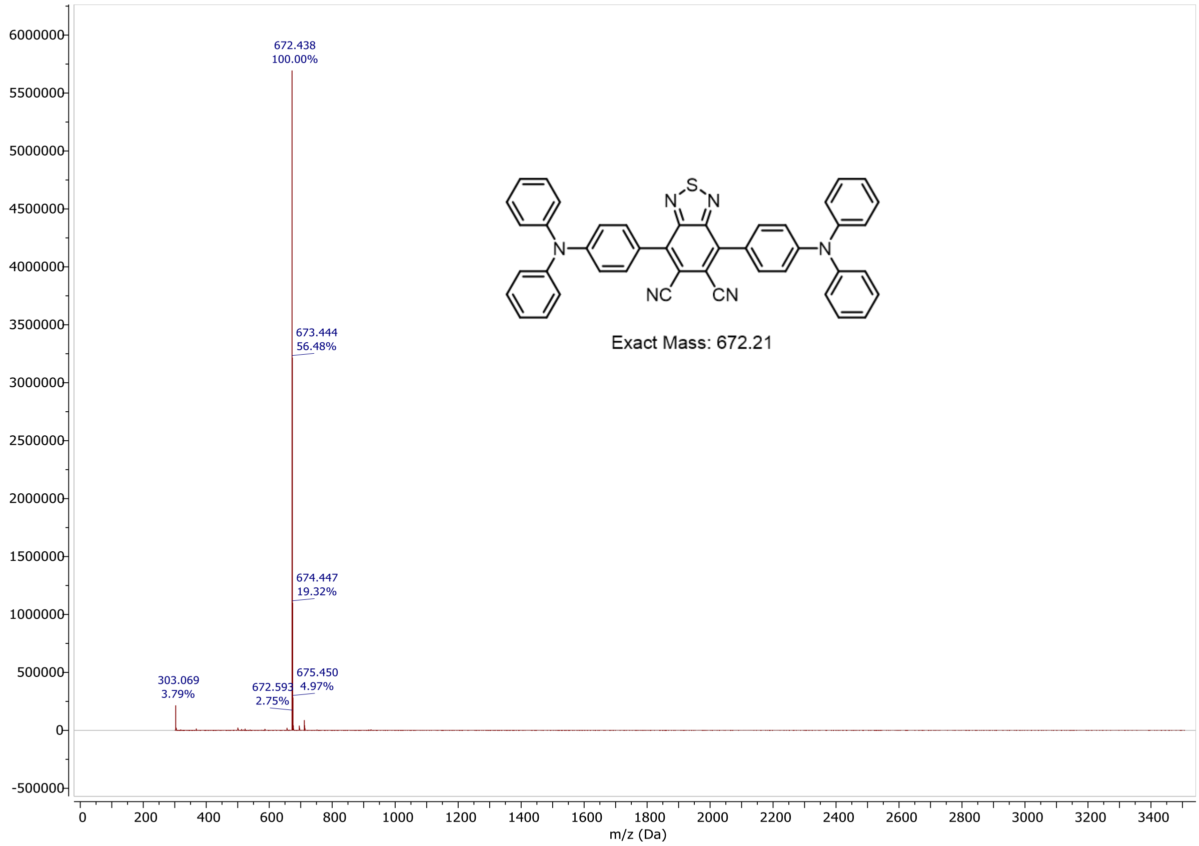

Figure S24: MALDI-ToF mass spectrum of 4,7-bis(4-(diphenylamino)phenyl)benzo[*c*][1,2,5]thiadiazole-5,6-dicarbonitrile (**3**).

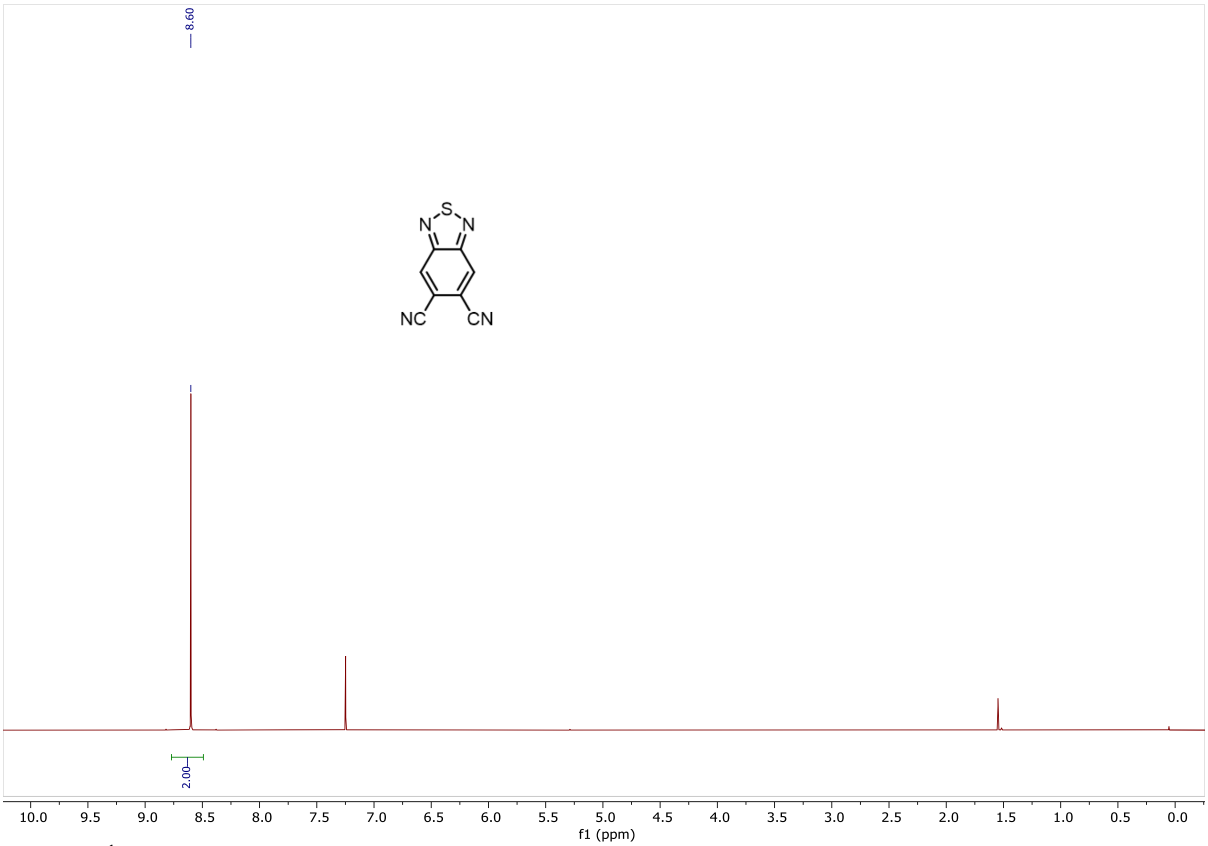

Figure S25: ^{1}H NMR spectrum of benzo[*c*][1,2,5]thiadiazole-5,6-dicarbonitrile (**5**) in $CDCl_3$.

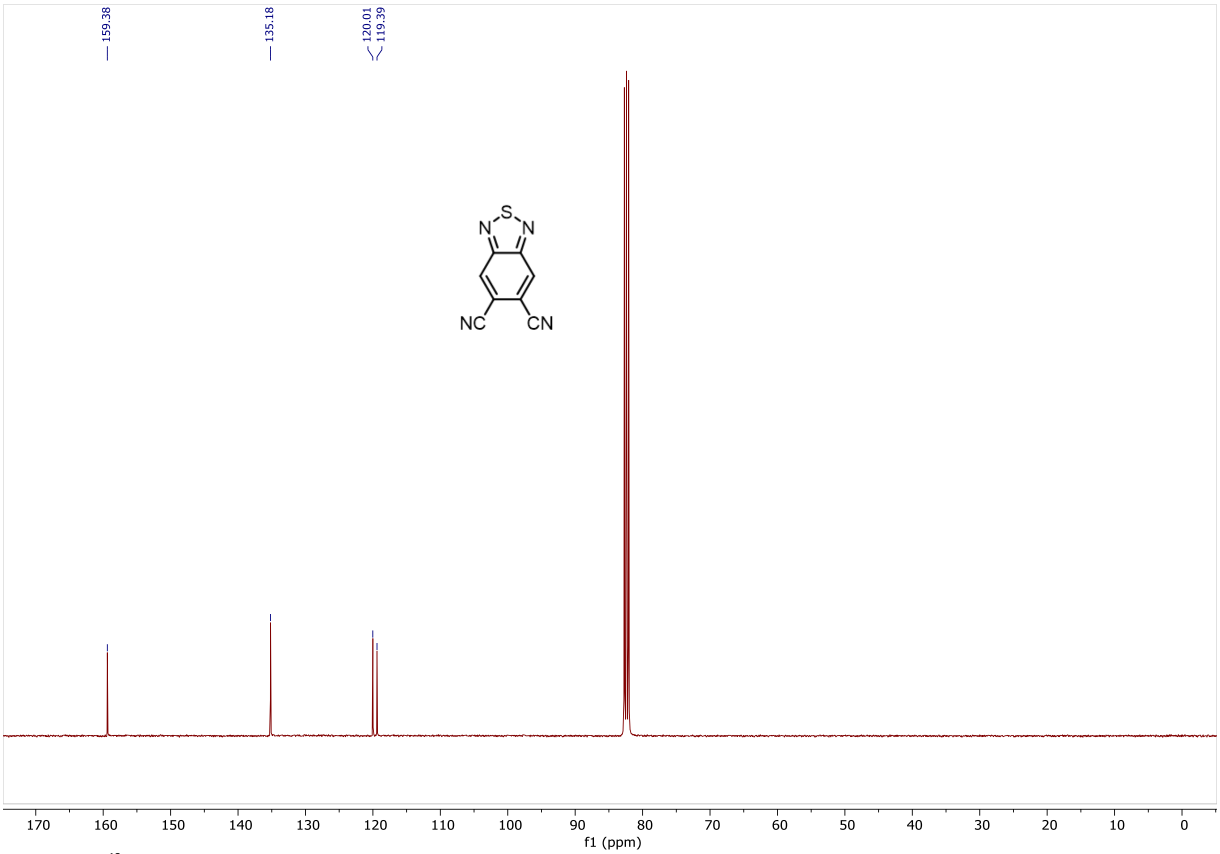

Figure S26: ^{13}C NMR spectrum of benzo[*c*][1,2,5]thiadiazole-5,6-dicarbonitrile (**5**) in $CDCl_3$.

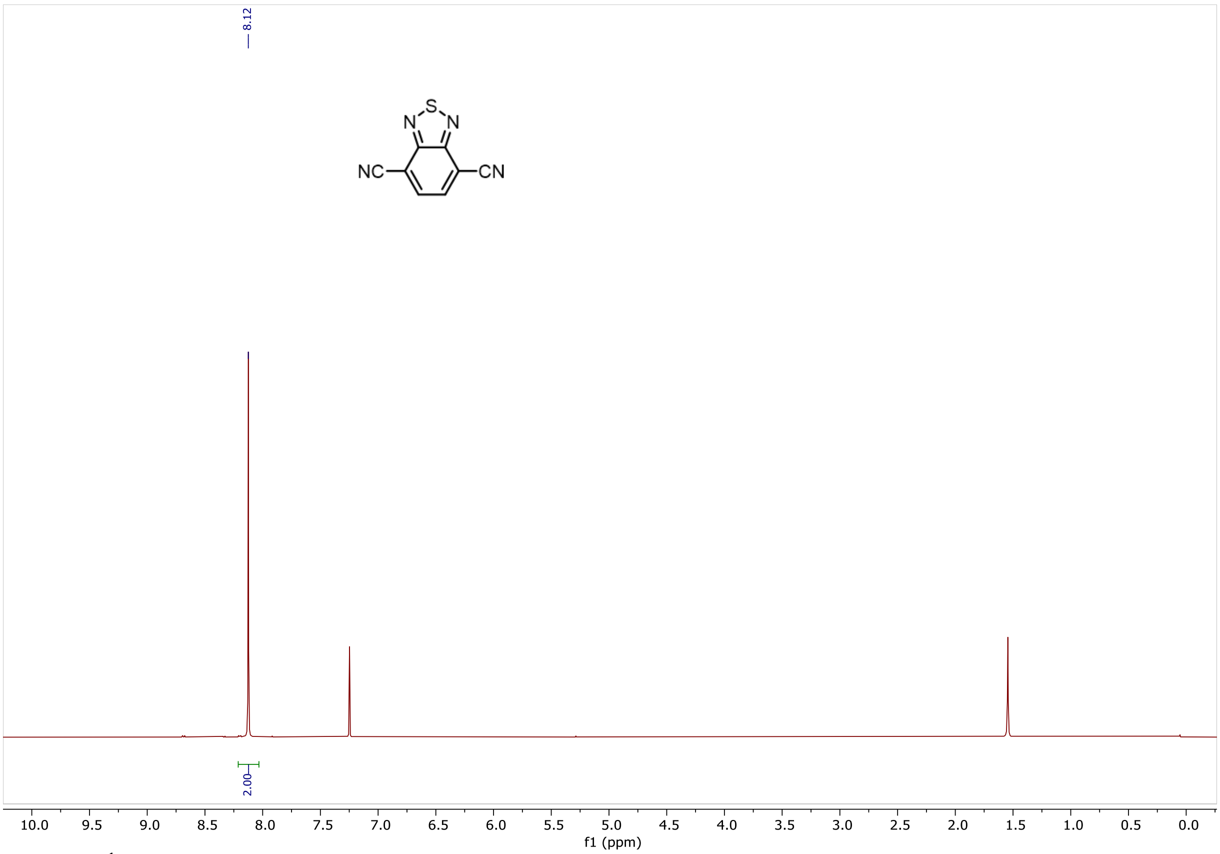

Figure S27: ^{1}H NMR spectrum of benzo[*c*][1,2,5]thiadiazole-4,7-dicarbonitrile (**9**) in $CDCl_3$.

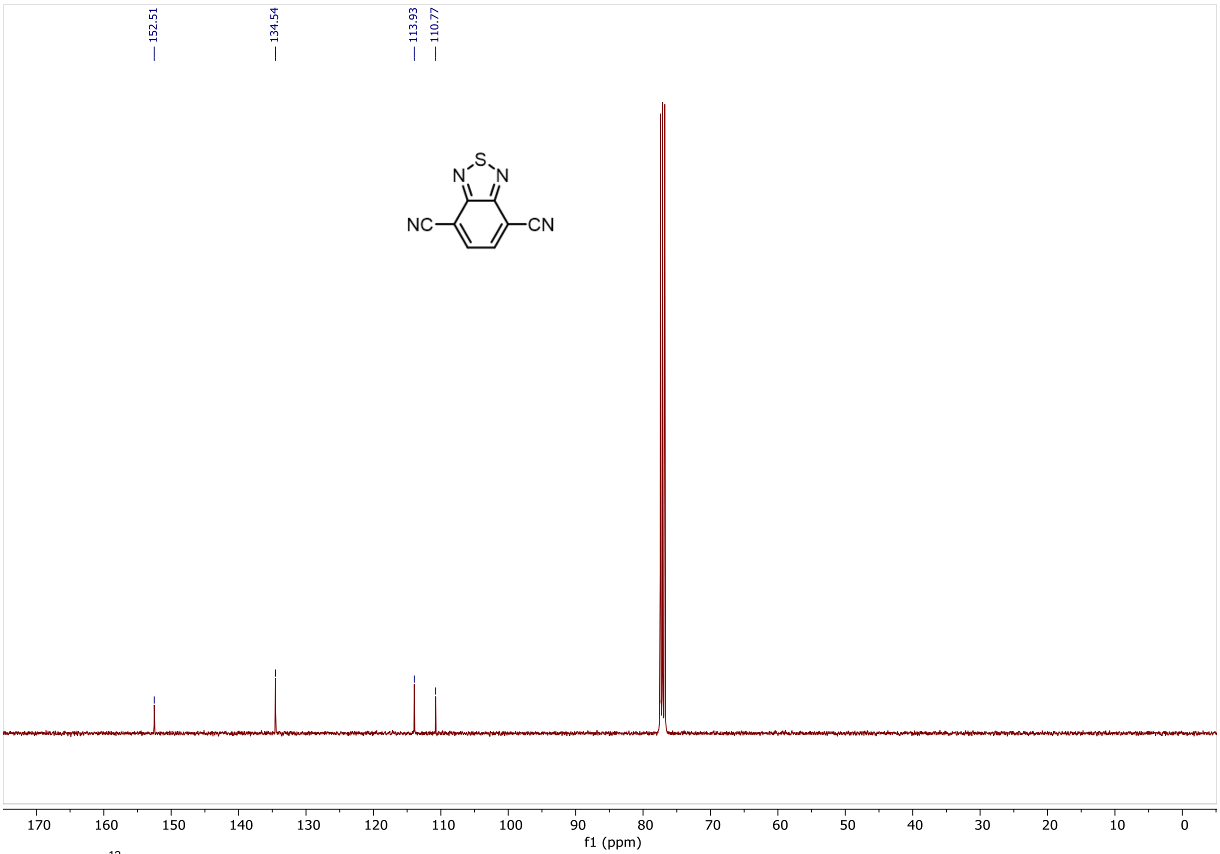

Figure S28: ^{13}C NMR spectrum of benzo[*c*][1,2,5]thiadiazole-4,7-dicarbonitrile (**9**) in $CDCl_3$.

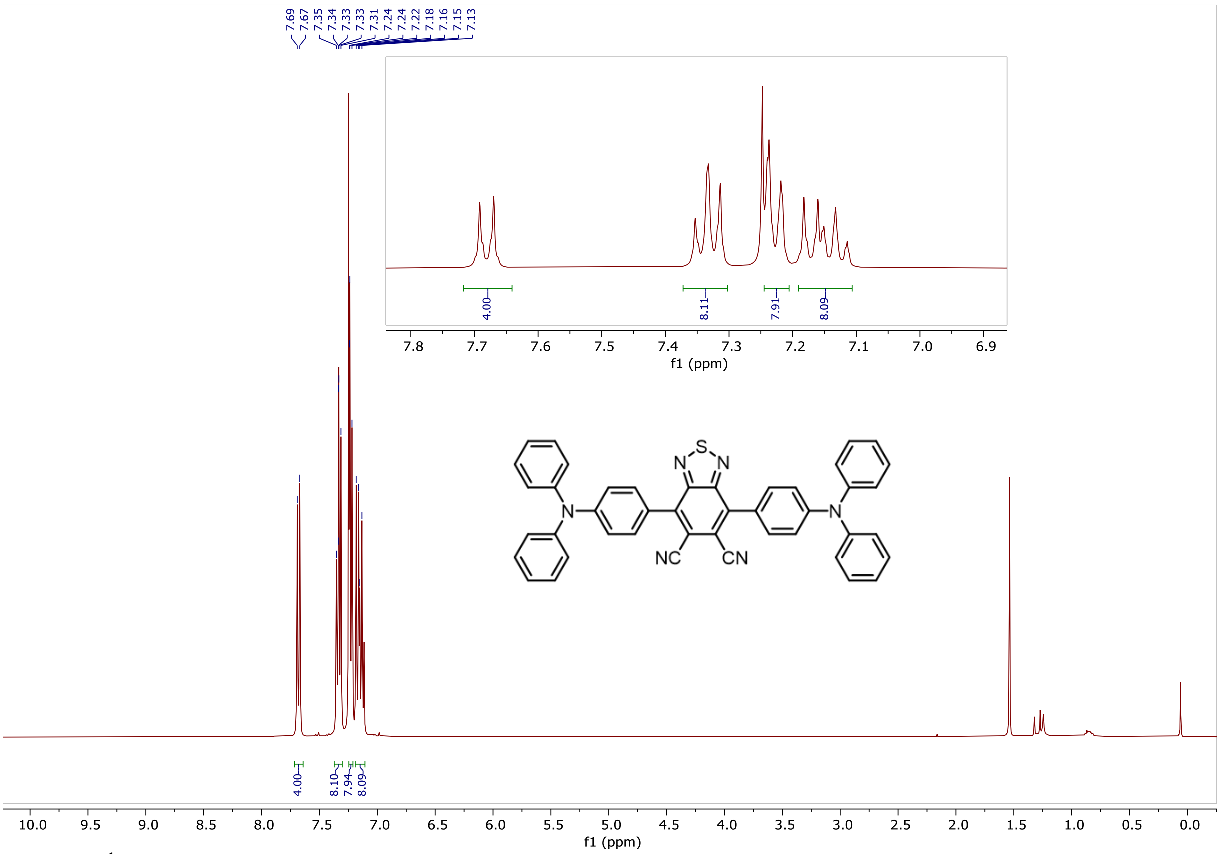

Figure S29: ^{1}H NMR spectrum of 4,7-bis(4-(diphenylamino)phenyl)benzo[*c*][1,2,5]thiadiazole-5,6-dicarbonitrile (**2TPA-iCNBT**, **6**) in $CDCl_3$.

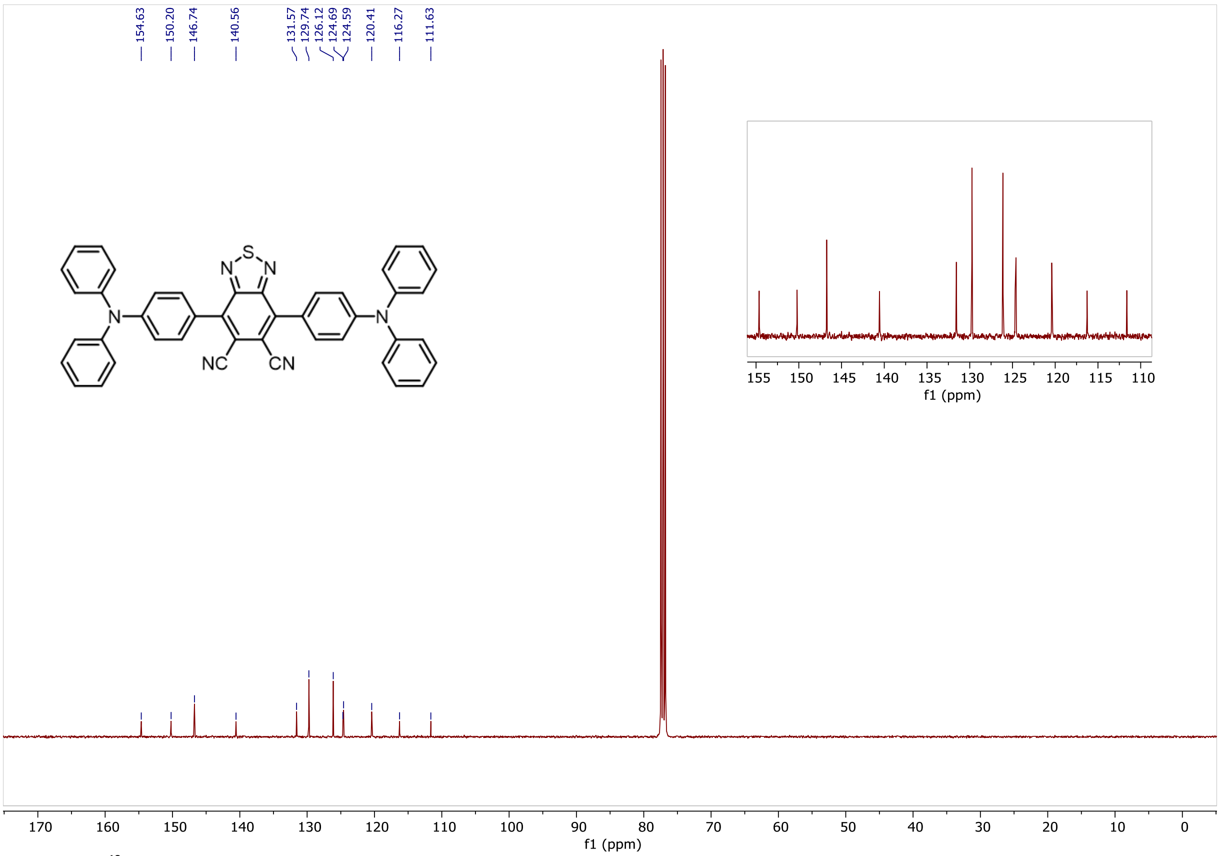

Figure S30: ^{13}C NMR spectrum of 4,7-bis(4-(diphenylamino)phenyl)benzo[*c*][1,2,5]thiadiazole-5,6-dicarbonitrile (**2TPA-iCNBT**, **6**) in $CDCl_3$.

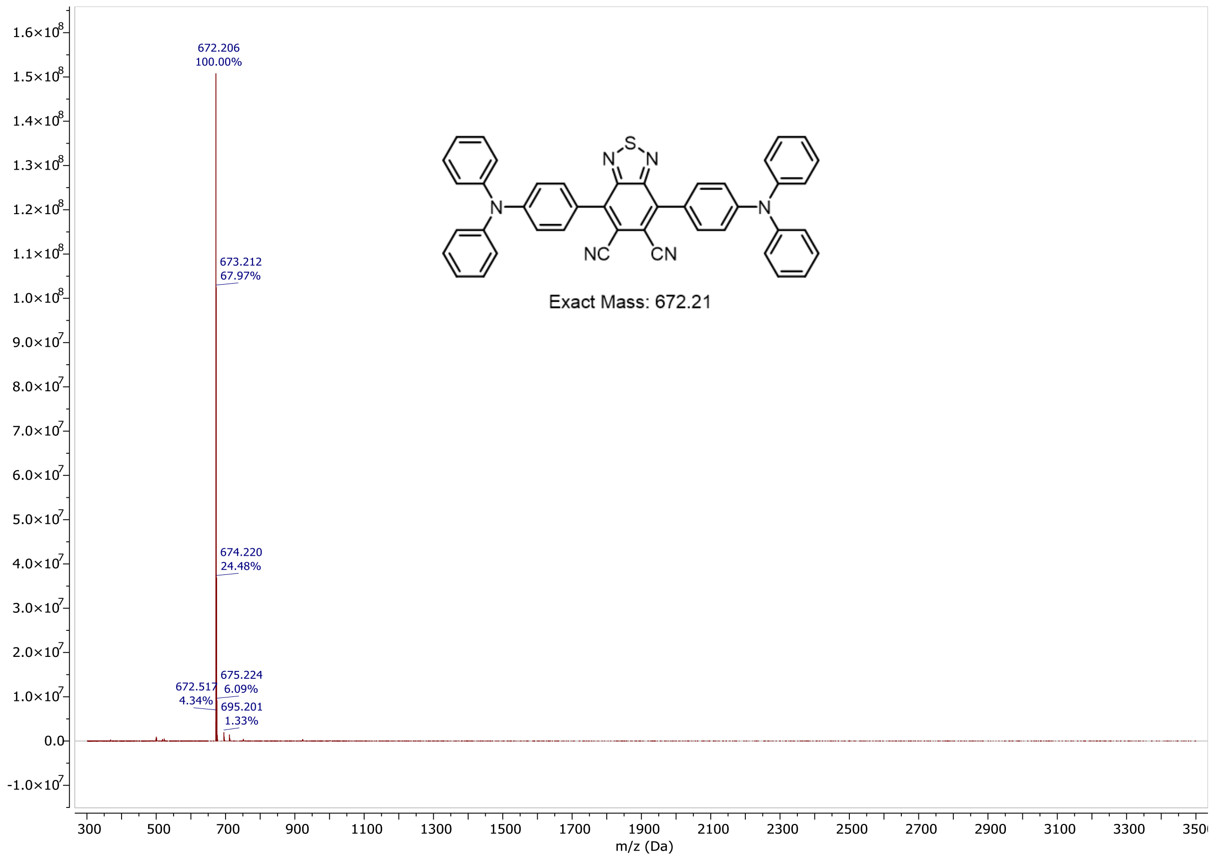

Figure S31: MALDI-ToF mass spectrum of 4,7-bis(4-(diphenylamino)phenyl)benzo[*c*][1,2,5]thiadiazole-5,6-dicarbonitrile (**2TPA-iCNBT**, **6**).

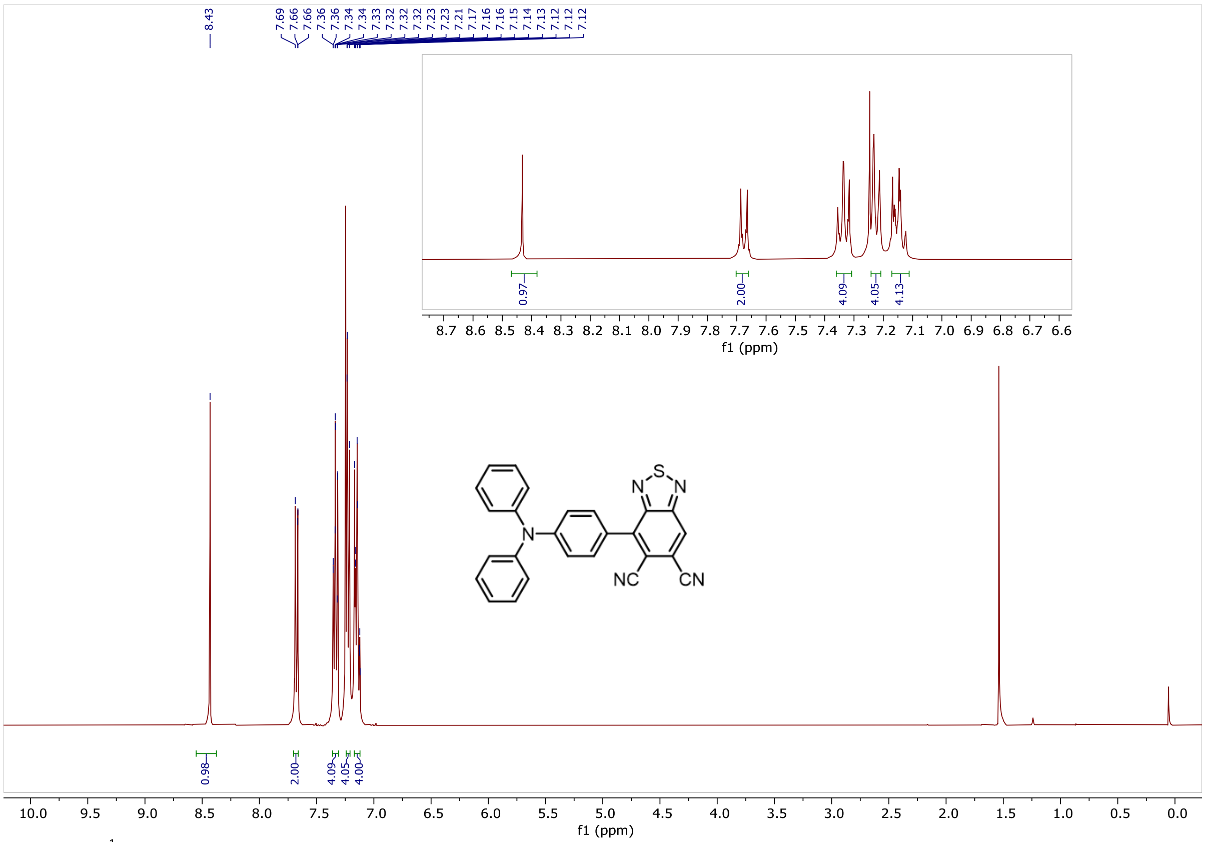

Figure S32: ^{1}H NMR spectrum of 4-(4-(diphenylamino)phenyl)benzo[*c*][1,2,5]thiadiazole-5,6-dicarbonitrile (**1TPA-iCNBT**, **7**) in $CDCl_3$.

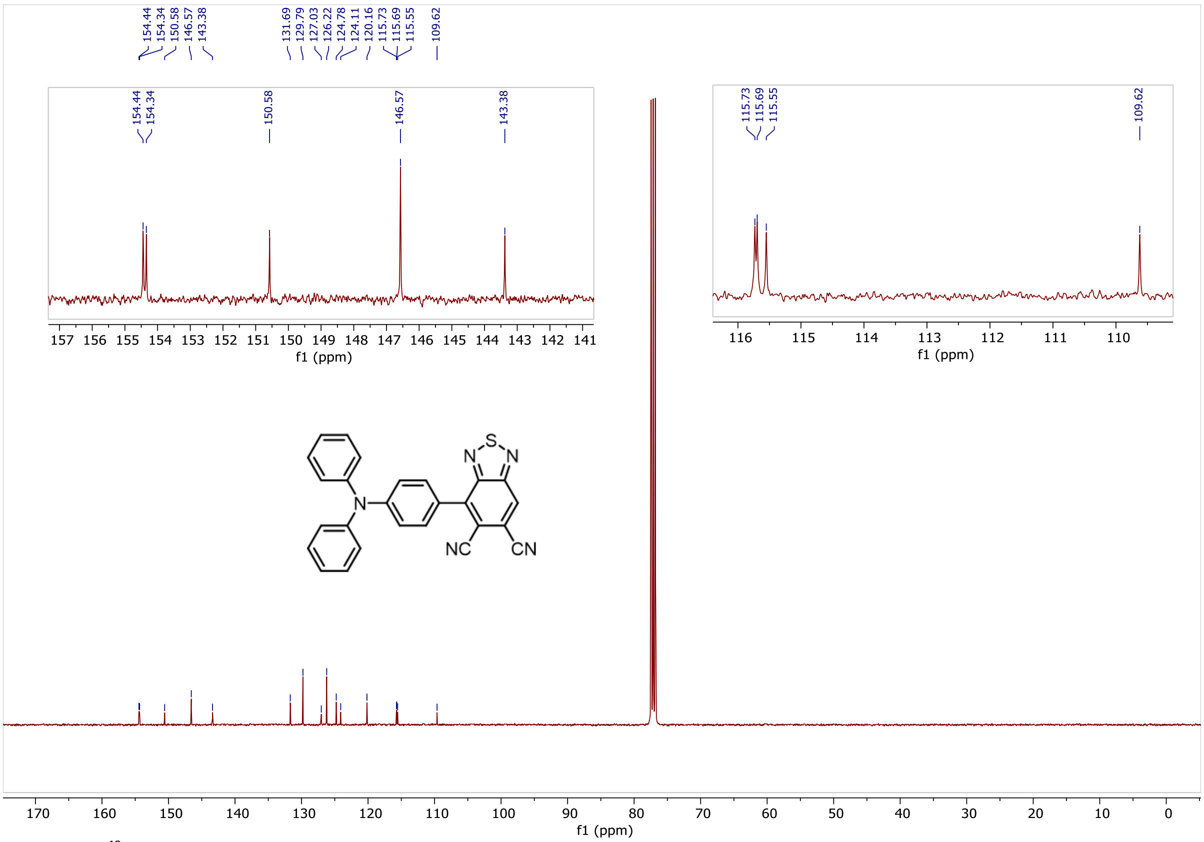

Figure S33: ^{13}C NMR spectrum of 4-(4-(diphenylamino)phenyl)benzo[*c*][1,2,5]thiadiazole-5,6-dicarbonitrile (**1TPA-iCNBT**, **7**) in $CDCl_3$.

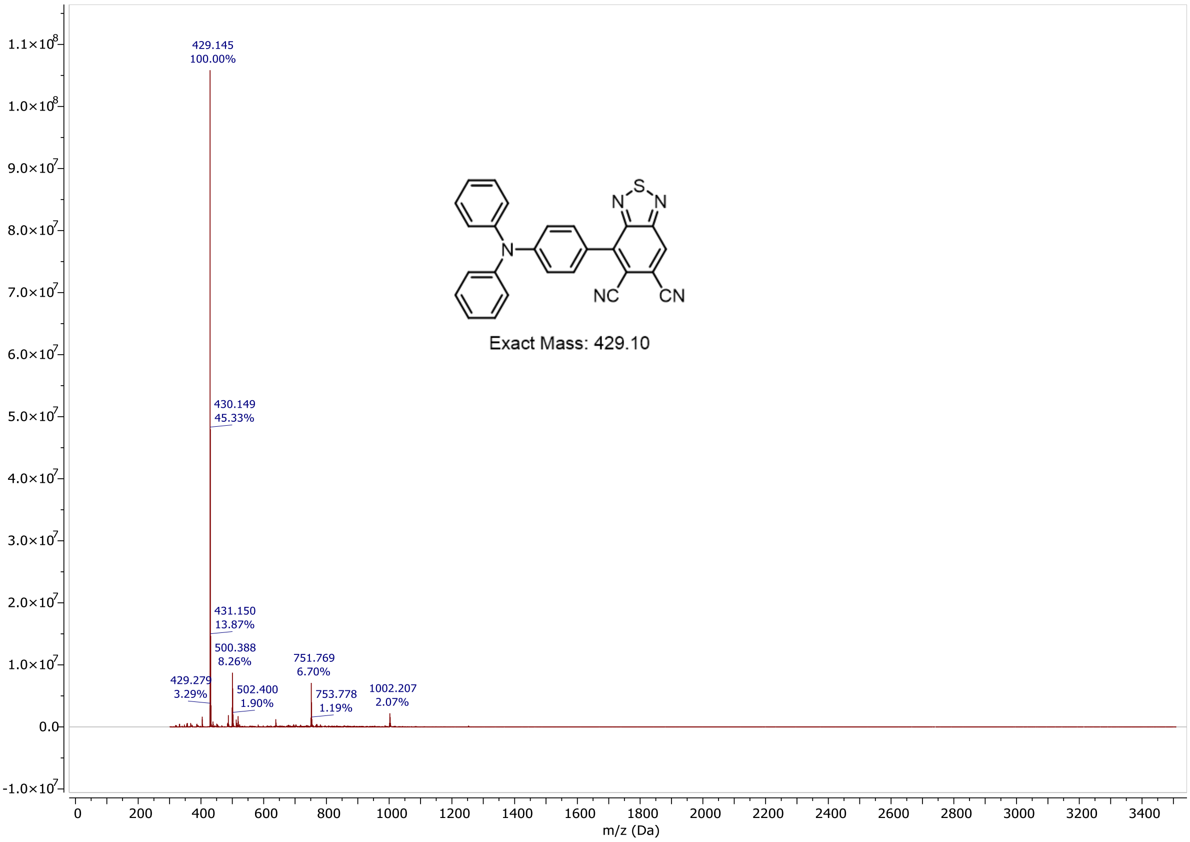

Figure S34: MALDI-ToF mass spectrum of 4-(4-(diphenylamino)phenyl)benzo[*c*][1,2,5]thiadiazole-5,6-dicarbonitrile (**1TPA-iCNBT**, **7**).

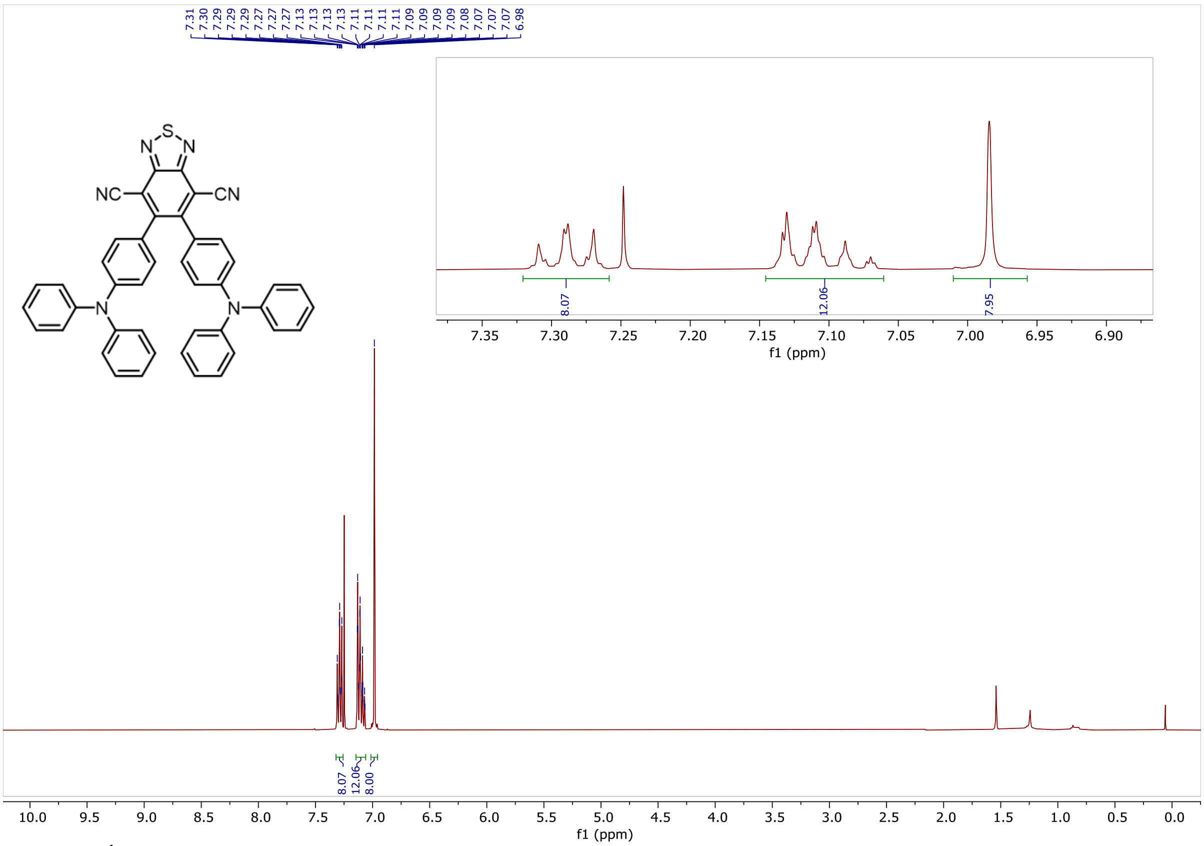

Figure S35: ^{1}H NMR spectrum of 5,6-bis(4-(diphenylamino)phenyl)benzo[*c*][1,2,5]thiadiazole-4,7-dicarbonitrile (**2TPA-iCNBT**, **10**) in $CDCl_3$.

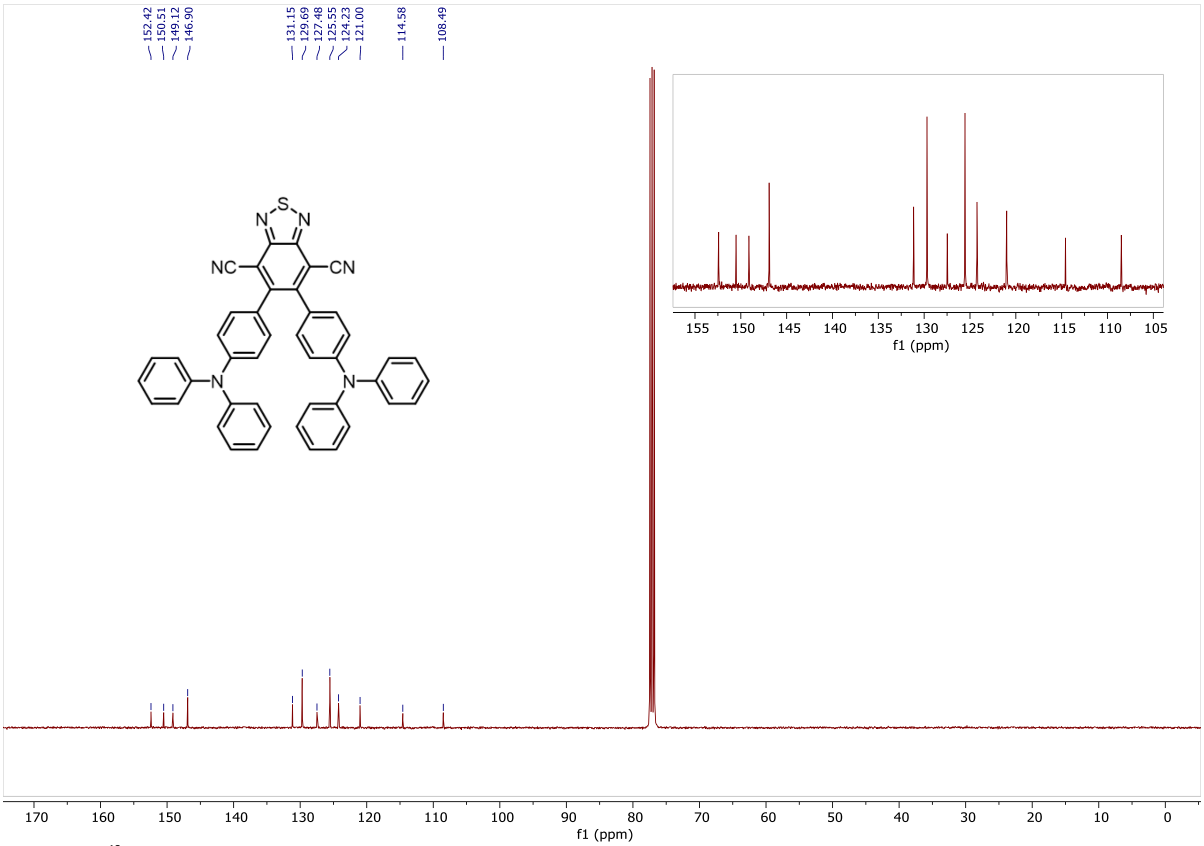

Figure S36: ^{13}C NMR spectrum of 5,6-bis(4-(diphenylamino)phenyl)benzo[*c*][1,2,5]thiadiazole-4,7-dicarbonitrile (**2TPA-iCNBT**, **10**) in $CDCl_3$.

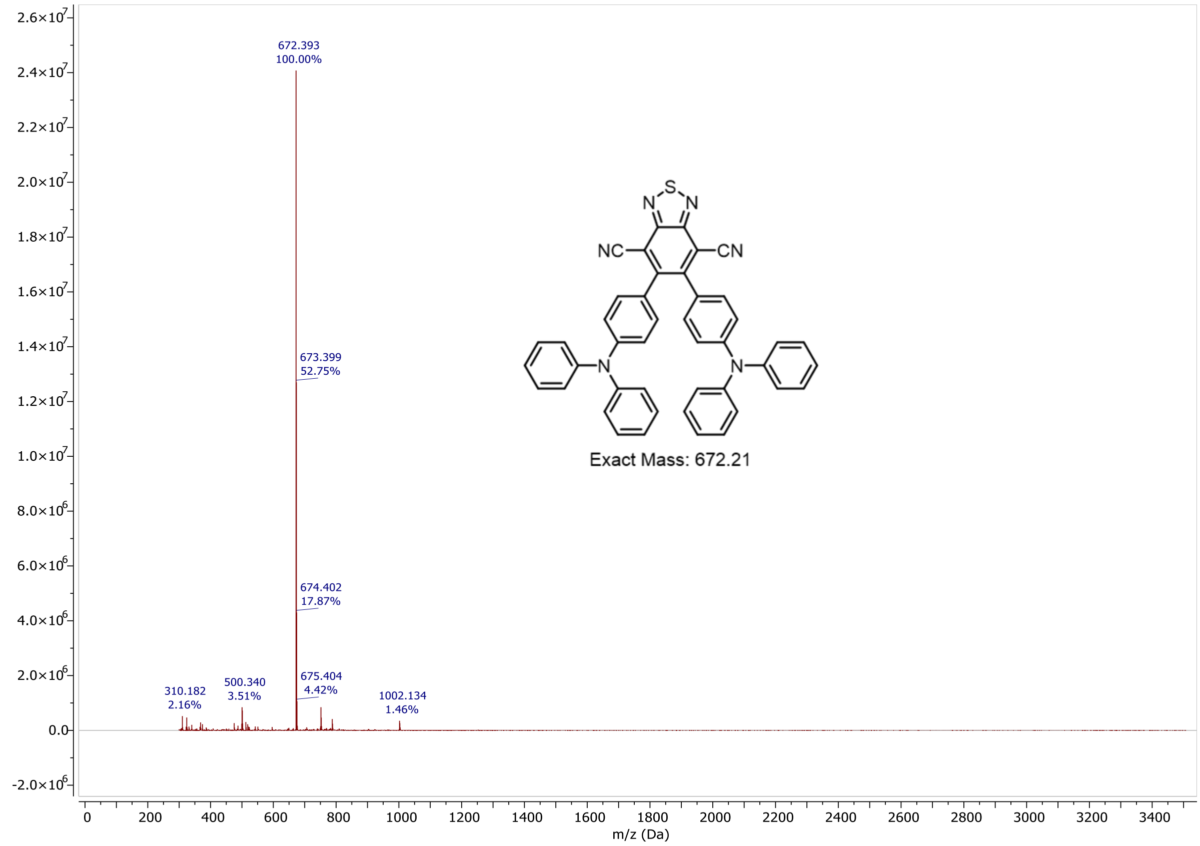

Figure S37: MALDI-ToF mass spectrum of 5,6-bis(4-(diphenylamino)phenyl)benzo[*c*][1,2,5]thiadiazole-4,7-dicarbonitrile (**2TPA-iCNBT**, **10**).

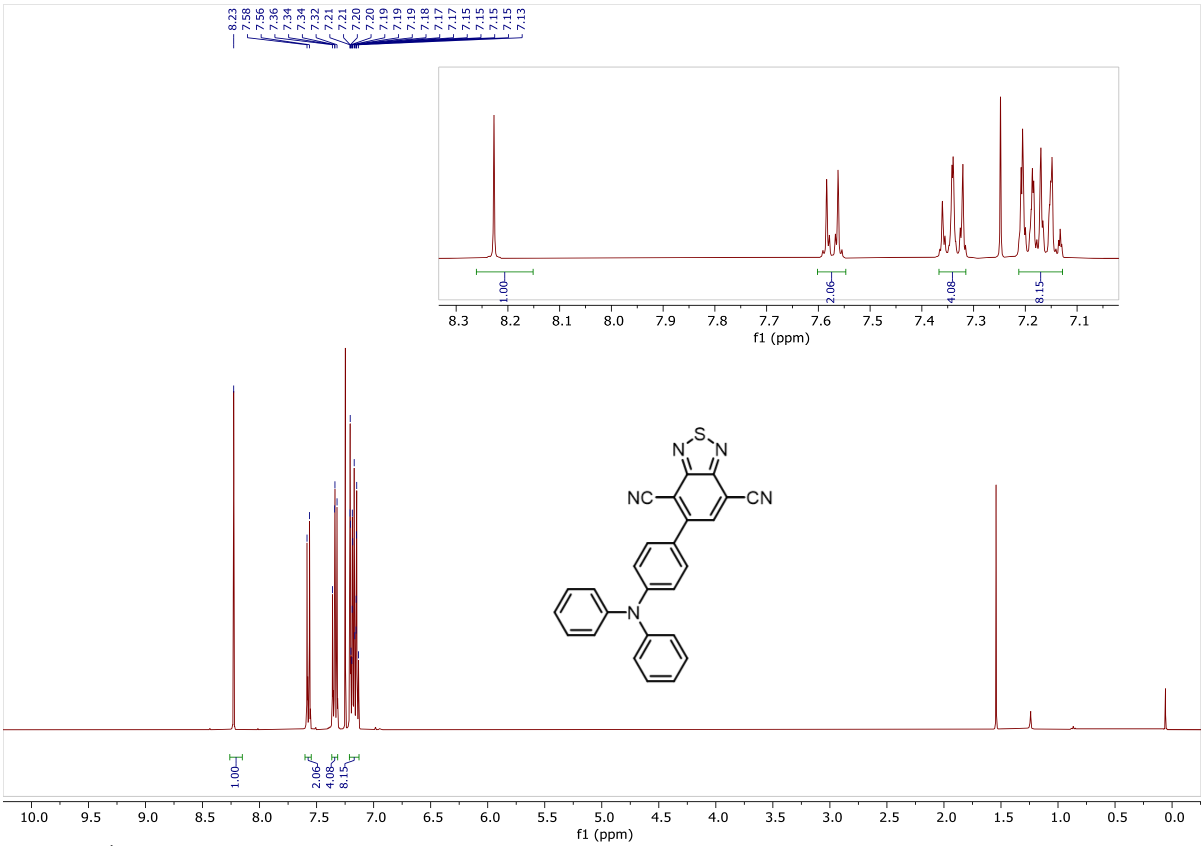

Figure S38: ^{1}H NMR spectrum of 5-(4-(diphenylamino)phenyl)benzo[*c*][1,2,5]thiadiazole-4,7-dicarbonitrile (**1TPA-iCNBT**, **11**) in $CDCl_3$.

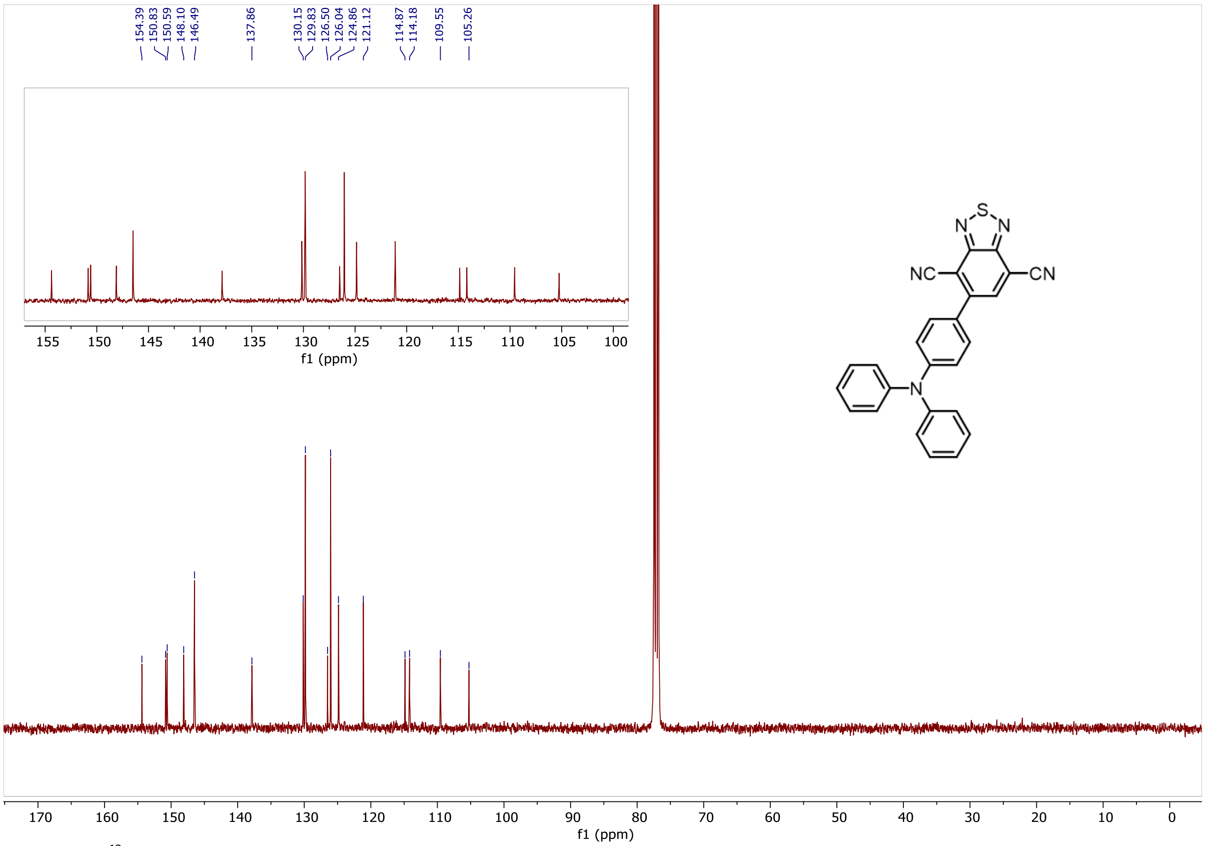

Figure S39: ^{13}C NMR spectrum of 5-(4-(diphenylamino)phenyl)benzo[*c*][1,2,5]thiadiazole-4,7-dicarbonitrile (**1TPA-iCNBT**, **11**) in $CDCl_3$.

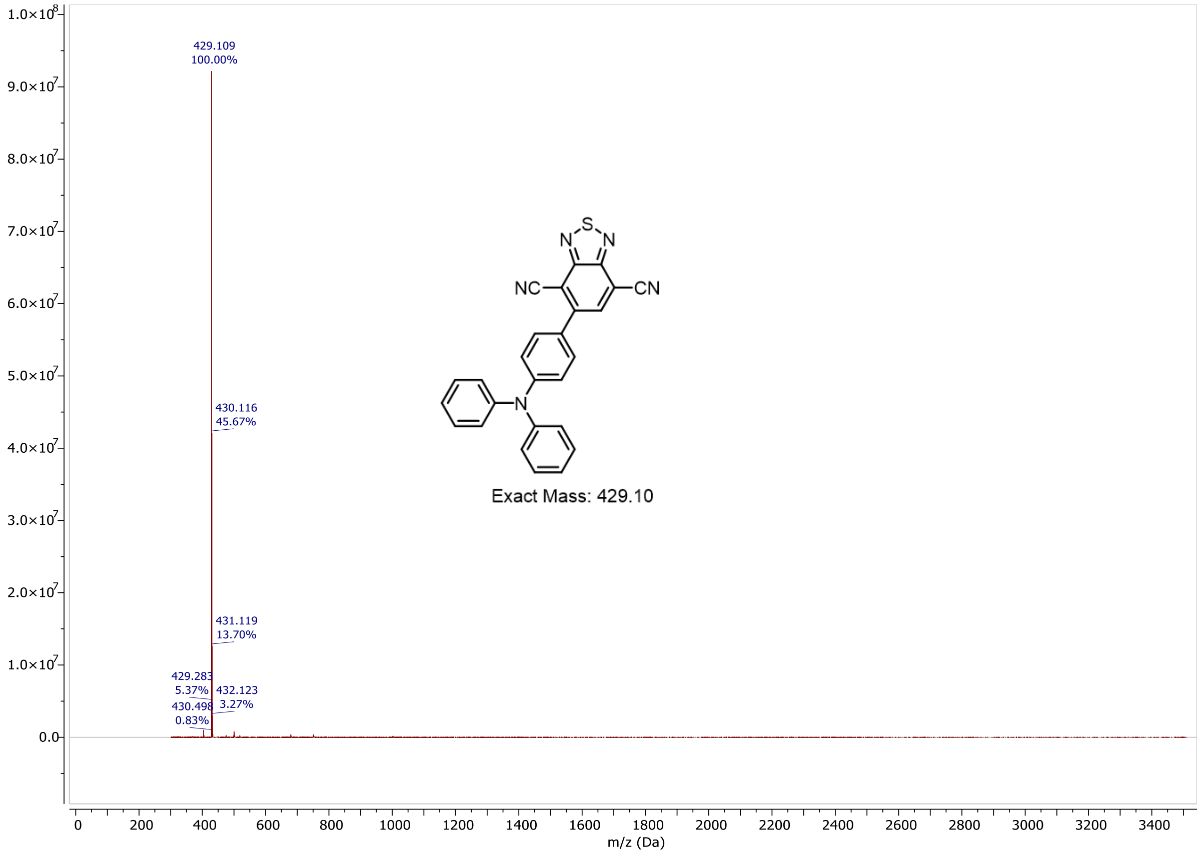

Figure S40: MALDI-ToF mass spectrum of 5-(4-(diphenylamino)phenyl)benzo[*c*][1,2,5]thiadiazole-4,7-dicarbonitrile (**1TPA-iCNBT**, **11**).

13. Coordinates of optimized geometries

1TPA-CNBT

C	3.43829	0.99481	0.82776
C	2.70626	0.08909	0.0799
C	3.46919	-0.84633	-0.69235
C	4.90686	-0.82365	-0.6923
C	5.61194	0.1277	0.07789
C	4.88156	1.01493	0.81553
N	2.96435	-1.78457	-1.49036
S	4.22463	-2.5616	-2.15617
N	5.45614	-1.74749	-1.47733
N	-2.97304	0.00078	-0.00507
C	-1.57539	0.02394	0.00975
C	-0.8408	-1.1579	0.16836
C	0.53772	-1.13405	0.17598
C	1.24146	0.06827	0.04818
C	0.50333	1.24431	-0.11295
C	-0.87593	1.22671	-0.13869
C	-3.70373	0.93854	-0.77973
C	-4.78442	1.61742	-0.22092
C	-5.50928	2.51866	-0.98492
C	-5.15826	2.7622	-2.30608
C	-4.07914	2.08805	-2.86281
C	-3.35983	1.17383	-2.1098
C	-3.68949	-0.94968	0.76666
C	-4.75599	-1.64684	0.20261
C	-5.46995	-2.55968	0.96303
C	-5.12191	-2.79708	2.28612
C	-4.05717	-2.10464	2.84808
C	-3.34896	-1.17869	2.09884
C	5.57327	1.98192	1.60606
N	6.1478	2.75768	2.23692
C	2.78451	1.91707	1.69754
N	2.29289	2.66729	2.42368
H	6.69694	0.14542	0.07418
H	-1.36476	-2.1042	0.27489
H	1.08206	-2.06735	0.29021
H	1.01667	2.19527	-0.23593
H	-1.4244	2.15633	-0.26468
H	-5.05079	1.43233	0.81762
H	-6.35	3.04439	-0.53826
H	-5.72491	3.47421	-2.90086
H	-3.80175	2.26439	-3.89953
H	-2.52347	0.63319	-2.54807
H	-5.02124	-1.46513	-0.83687
H	-6.30052	-3.09821	0.51253
H	-5.68057	-3.5172	2.87871
H	-3.78322	-2.27442	3.88679
H	-2.52593	-0.62259	2.54279

1TPA-iCNBT

C	3.04624	0.87148	1.15291
C	4.40525	0.96631	1.27555
C	5.22124	0.25274	0.35065
C	4.59646	-0.53201	-0.67237
C	3.17483	-0.59725	-0.77329
C	2.39755	0.09111	0.14596
N	6.54582	0.22675	0.31461
S	6.94855	-0.72865	-0.94055
N	5.46575	-1.13735	-1.4732
N	-3.28748	0.04088	0.10574
C	-1.8923	0.03961	0.12745
C	-1.18212	1.22688	0.35093
C	0.19635	1.22944	0.36147
C	0.93008	0.05903	0.13908
C	0.21589	-1.12432	-0.07106
C	-1.16372	-1.13911	-0.0743
C	-4.03061	-1.11624	0.45716
C	-5.07319	-1.54754	-0.36002
C	-5.81719	-2.66265	-0.00803
C	-5.52266	-3.36591	1.15225

C	-4.48136	-2.93845	1.96586
C	-3.74355	-1.81471	1.62862
C	-3.99969	1.18691	-0.33983
C	-3.65489	1.81026	-1.53728
C	-4.36139	2.92133	-1.96998
C	-5.42688	3.40922	-1.22473
C	-5.77902	2.77983	-0.03817
C	-5.06721	1.67712	0.40815
C	4.99893	1.7483	2.3063
N	5.46793	2.38683	3.14428
C	2.62233	-1.32764	-1.86196
N	2.20206	-1.91689	-2.76084
H	2.42145	1.39172	1.87443
H	-1.72481	2.15317	0.51942
H	0.71593	2.17265	0.52136
H	0.74676	-2.06085	-0.22351
H	-1.68948	-2.07431	-0.24608
H	-5.29611	-1.00046	-1.27361
H	-6.62839	-2.99082	-0.65368
H	-6.10402	-4.24352	1.4235
H	-4.2488	-3.47635	2.88203
H	-2.93835	-1.4685	2.27334
H	-2.83014	1.41512	-2.12685
H	-4.08568	3.40058	-2.90645
H	-5.98398	4.27658	-1.56995
H	-6.61108	3.15581	0.55259
H	-5.33484	1.1835	1.34027

2TPA-CNBT

C	-0.72232	-0.01467	1.23864
C	-1.47365	-0.00729	0.07995
C	-0.71974	-0.00339	-1.13652
C	0.71974	0.00343	-1.13652
C	1.47364	0.00729	0.07995
C	0.72232	0.01463	1.23864
C	2.93988	-0.00413	0.05909
C	-2.93988	0.00413	0.05909
C	-3.65422	-0.84732	-0.79043
C	-5.03324	-0.85312	-0.80291
C	-5.75987	0.00773	0.02872
C	-5.05076	0.87314	0.86884
C	-3.6708	0.86591	0.88158
C	3.6708	-0.86594	0.88155
C	5.05075	-0.87317	0.86881
C	5.75987	-0.00773	0.02872
C	5.03324	0.85314	-0.80288
C	3.65422	0.84734	-0.7904
N	7.15832	-0.00489	0.01512
N	-7.15832	0.00488	0.01512
C	-7.89058	1.18634	0.30001
C	-7.87905	-1.18283	-0.27218
C	7.87905	1.18284	-0.27213
C	7.89058	-1.18635	0.29996
C	-8.942	-1.15172	-1.17254
C	-9.66309	-2.30524	-1.43838
C	-9.32538	-3.50252	-0.82161
C	-8.264	-3.53523	0.07355
C	-7.54871	-2.3823	0.35644
C	-8.95956	1.14559	1.19259
C	-9.69069	2.29342	1.4556
C	-9.35726	3.49437	0.84373
C	-8.2896	3.53681	-0.04346
C	-7.56421	2.38951	-0.3234
C	8.942	1.15176	-1.1725
C	9.66309	2.30529	-1.4383
C	9.32539	3.50255	-0.82149
C	8.26401	3.53522	0.07367
C	7.54872	2.38228	0.35652
C	8.95955	-1.14564	1.19256
C	9.69069	-2.29347	1.45552
C	9.35726	-3.4944	0.8436
C	8.2896	-3.53681	-0.04358
C	7.56421	-2.3895	-0.32349
N	1.2434	-0.00534	-2.35944
S	0.	0.00007	-3.40333
N	-1.2434	0.00543	-2.35944

C	1.36675	0.10066	2.50953
N	1.85856	0.18835	3.54981
C	-1.36675	-0.10076	2.50953
N	-1.85856	-0.18848	3.5498
H	-3.11772	-1.52137	-1.45217
H	-5.56298	-1.52638	-1.47181
H	-5.59105	1.55171	1.52342
H	-3.15128	1.55772	1.54057
H	3.15128	-1.55777	1.54051
H	5.59105	-1.55176	1.52336
H	5.56298	1.52643	-1.47176
H	3.11772	1.52142	-1.45212
H	-9.19888	-0.21367	-1.66048
H	-10.49089	-2.26903	-2.1429
H	-9.88935	-4.40704	-1.03542
H	-7.99798	-4.46534	0.57057
H	-6.72799	-2.4028	1.07037
H	-9.21204	0.20519	1.67817
H	-10.52224	2.25008	2.1552
H	-9.9285	4.39456	1.0564
H	-8.02579	4.47042	-0.53511
H	-6.73697	2.41751	-1.0296
H	9.19887	0.21372	-1.66047
H	10.49089	2.2691	-2.14282
H	9.88935	4.40707	-1.03527
H	7.99799	4.46531	0.57072
H	6.72799	2.40276	1.07045
H	9.21203	-0.20525	1.67816
H	10.52224	-2.25015	2.15512
H	9.9285	-4.3946	1.05625
H	8.02579	-4.47041	-0.53527
H	6.73697	-2.41747	-1.02969

2TPA-CNBT

C	1.50074	1.71577	0.07132
C	1.21296	0.8059	1.0906
C	1.86261	-0.40862	1.16772
C	2.81019	-0.77458	0.20257
C	3.07436	0.12101	-0.83942
C	2.44356	1.34871	-0.88834
C	-1.40128	1.87309	-0.11496
C	-2.3586	1.57573	0.85394
C	-3.09072	0.40515	0.80046
C	-2.91357	-0.49702	-0.25353
C	-1.96279	-0.19287	-1.23645
C	-1.21305	0.9618	-1.15593
N	3.48545	-1.99698	0.28131
C	4.80875	-2.12918	-0.2181
C	2.91571	-3.10043	0.96591
N	-3.67223	-1.67042	-0.32784
C	-3.16765	-2.81986	-0.9865
C	-4.9869	-1.72172	0.20576
C	3.66138	-3.79616	1.91504
C	3.10637	-4.87788	2.58135
C	1.79986	-5.26835	2.32176
C	1.05447	-4.57565	1.37621
C	1.61068	-3.50619	0.69136
C	5.14885	-3.24138	-0.98479
C	6.44478	-3.39488	-1.45187
C	7.40955	-2.43651	-1.17146
C	7.07032	-1.32554	-0.41025
C	5.78051	-1.17387	0.07407
C	-3.96088	-3.50433	-1.90516
C	-3.47208	-4.63335	-2.54391
C	-2.18446	-5.08414	-2.28757
C	-1.39146	-4.40233	-1.37342
C	-1.88135	-3.28457	-0.71552
C	-5.37566	-2.80978	0.98439
C	-6.6652	-2.8807	1.48744
C	-7.57469	-1.86316	1.23237
C	-7.18667	-0.77601	0.46
C	-5.90393	-0.70662	-0.06054
C	-0.60456	3.11242	-0.03892
C	0.83807	3.03266	0.01639
C	-1.2387	4.34016	-0.04434

C	-0.46498	5.53606	-0.00133
C	0.96251	5.45771	0.02388
C	1.60156	4.18454	0.04538
N	-0.93035	6.77839	-0.004
N	1.56083	6.64162	0.04814
S	0.37242	7.75546	0.03112
C	3.02051	4.14769	0.16487
N	4.16947	4.14604	0.27063
C	-2.65382	4.45775	-0.15746
N	-3.79673	4.58104	-0.25624
H	0.48825	1.06748	1.85915
H	1.65054	-1.07852	1.99661
H	3.78735	-0.1492	-1.61368
H	2.6685	2.0272	-1.70968
H	-2.5106	2.25806	1.68875
H	-3.81086	0.18341	1.58352
H	-1.82298	-0.86704	-2.07719
H	-0.48275	1.17448	-1.93425
H	4.68066	-3.48064	2.12761
H	3.69857	-5.41188	3.32084
H	1.36342	-6.11067	2.85242
H	0.03105	-4.87873	1.16227
H	1.03837	-2.9682	-0.0637
H	4.38727	-3.98568	-1.20868
H	6.69951	-4.267	-2.04964
H	8.42388	-2.55533	-1.54419
H	7.82021	-0.57301	-0.17805
H	5.51809	-0.30953	0.68034
H	-4.96514	-3.14237	-2.11517
H	-4.10124	-5.15765	-3.25943
H	-1.79963	-5.9641	-2.79665
H	-0.38214	-4.75252	-1.16353
H	-1.26955	-2.75498	0.01429
H	-4.65737	-3.60096	1.18965
H	-6.95775	-3.73477	2.09391
H	-8.58379	-1.91728	1.6332
H	-7.89344	0.02267	0.24741
H	-5.604	0.13889	-0.67595

14. References

[1] A.M. Brouwer, Standards for photoluminescence quantum yield measurements in solution (IUPAC Technical Report), Pure and Applied Chemistry 83(12) (2011) 2213-2228. https://doi.org/doi:10.1351/PAC-REP-10-09-31.
[2] F. Wilkinson, W.P. Helman, A.B. Ross, Quantum Yields for the Photosensitized Formation of the Lowest Electronically Excited Singlet State of Molecular Oxygen in Solution, Journal of Physical and Chemical Reference Data 22(1) (1993) 113-262. https://doi.org/10.1063/1.555934.
[3] S. Trasatti, The absolute electrode potential: an explanatory note (Recommendations 1986), Pure and Applied Chemistry 58(7) (1986) 955-966. https://doi.org/doi:10.1351/pac198658070955.
[4] A.J. Bard, L.R. Faulkner, H.S. White, Electrochemical Methods: Fundamentals and Applications, 3rd Edition, 2022.
[5] J. Kumsampao, C. Chaiwai, P. Chasing, T. Chawanpunyawat, S. Namuangruk, T. Sudyoadsuk, V. Promarak, A Simple and Strong Electron-Deficient 5,6-Dicyano[2,1,3]benzothiadiazole-Cored Donor-Acceptor-Donor Compound for Efficient Near Infrared Thermally Activated Delayed Fluorescence, Chemistry – An Asian Journal 15(19) (2020) 3029-3036. https://doi.org/https://doi.org/10.1002/asia.202000727.
[6] M. Fraiponts, W. Maes, B. Champagne, Earth Mover's Charge Transfer Distance: A General and Robust Approach for Describing Excited State Locality, Journal of Chemical Theory and Computation 20(7) (2024) 2751-2760. https://doi.org/10.1021/acs.jctc.3c01148.
[7] N. Haase, A. Danos, C. Pflumm, A. Morherr, P. Stachelek, A. Mekic, W. Brütting, A.P. Monkman, Kinetic Modeling of Transient Photoluminescence from Thermally Activated Delayed Fluorescence, The Journal of Physical Chemistry C 122(51) (2018) 29173-29179. https://doi.org/10.1021/acs.jpcc.8b11020.
[8] H. Wang, B. Zhao, C. Qu, C. Duan, Z. Li, P. Ma, P. Chang, C. Han, H. Xu, 2,3-Dicyanopyrazino phenanthroline enhanced charge transfer for efficient near-infrared thermally activated delayed fluorescent diodes, Chemical Engineering Journal 436 (2022) 135080. https://doi.org/https://doi.org/10.1016/j.cej.2022.135080.
[9] D.-H. Kim, A. D'Aléo, X.-K. Chen, A.D.S. Sandanayaka, D. Yao, L. Zhao, T. Komino, E. Zaborova, G. Canard, Y. Tsuchiya, E. Choi, J.W. Wu, F. Fages, J.-L. Brédas, J.-C. Ribierre, C. Adachi, High-efficiency electroluminescence and amplified spontaneous emission from a thermally activated delayed fluorescent near-infrared emitter, Nature Photonics 12(2) (2018) 98-104. https://doi.org/10.1038/s41566-017-0087-y.
[10] A.K. Gupta, D.B. Cordes, J. De, A.M.Z. Slawin, S. Warriner, I.D.W. Samuel, E. Zysman-Colman, Deep-red to NIR solution-processed OLEDs of donor-decorated quinoxaline-based TADF aggregates, Journal of Materials Chemistry C 13(12) (2025) 6123-6135. https://doi.org/10.1039/D4TC05238B.
[11] C. Lin, Z. Wu, J. Liu, W.-T. Deng, Y. Zhuang, T. Xuan, J. Xue, L. Zhang, G. Wei, R.-J. Xie, Extremely low efficiency roll-off in vacuum- and solution-processed deep-red/near-infrared OLEDs based on 1,8-naphthalimide TADF emitters, Journal of Luminescence 243 (2022) 118683. https://doi.org/https://doi.org/10.1016/j.jlumin.2021.118683.
